# Measuring Consistency Between Large Language Models’ Responses to Preventive Care Queries and Official US Preventive Services Task Force (USPSTF) Recommendations: Systematic Test Involving All USPSTF Preventive Care Topics via Simulated User Prompts

**DOI:** 10.2196/87034

**Published:** 2026-07-30

**Authors:** Tim Johnson, Wolfgang Gaissmaier

**Affiliations:** 1 Atkinson Graduate School of Management Willamette University Salem, OR United States; 2 Department of Psychology University of Konstanz Konstanz Germany

**Keywords:** generative artificial intelligence, health promotion, large language model, LLM, preventive health services, preventive medicine, primary prevention, public health

## Abstract

**Background:**

Large language models (LLMs) have the potential to provide individualized preventive care guidance at scale. Research, however, has found mixed performance among a small set of LLMs queried about select preventive care activities. These findings call for testing a larger set of LLMs on a wider range of preventive care topics.

**Objective:**

This study aims to assess whether various popular LLMs generate outputs about preventive care consistent with a comprehensive set of recommendations from the US Preventive Services Task Force (USPSTF).

**Methods:**

We investigated whether 35 popular LLMs produced outputs consistent with all publicly available USPSTF recommendations (n=142) published as of May 2025. The study occurred in 2 waves (wave 1, 2025: 28 LLMs; wave 2, 2026: 10 LLMs; 3 LLMs overlapping across waves). LLMs received queries from simulated users who, in baseline prompts, sought nonbinding, hypothetical advice about whether to participate in particular preventive care activities given their inclusion in a relevant population. LLM raters assessed LLM-USPSTF concordance (interrater reliability, wave 1: κ=0.8893; wave 2: κ=0.9366). Wave 2 tested chain-of-thought, few-shot, and role-based prompts (3 variants each for 426 tests per prompting approach per model). Wave 2 also tested an iterative prompt that sought clarification about previous LLM responses and a prompt that eliminated the user’s reference to nonbinding, hypothetical advice. Automated methods classified responses to detect sources of LLM-USPSTF discrepancy. Further tests prompted LLMs to rate preventive care activities for relevant populations using the USPSTF grading scale.

**Results:**

Focusing on cases where LLM raters agreed, the study found in its baseline prompts that the LLM with the highest concordance rate generated responses consistent with USPSTF recommendations in 66.92% (89/133) of tests in wave 1 and 87.77% (122/139) of tests in wave 2; the LLM with the lowest rate accorded with USPSTF recommendations in 45.19% (61/135) of tests in wave 1 and in 50.36% (69/137) of tests in wave 2. Eliminating reference to nonbinding, hypothetical advice did not alter the highest-performing model’s rate of concordance (122/139, 87.77%). The highest concordance rate increased with chain-of-thought (404/421, 95.96%), role-based (371/416, 89%), and iterative prompting (127/140, 90.71%); however, it moderately decreased with few-shot prompting (361/419, 86.15%). Automated content analysis found high rates of LLMs avoiding definitive recommendations. When prompted to grade preventive care activities, the highest-performing LLM matched USPSTF grades in 85.92% (122/142) of tests in wave 1 and in 94.37% (134/142) of tests in wave 2.

**Conclusions:**

LLMs’ consistency with USPSTF recommendations varies. Deviations result mainly from LLMs’ avoidance of definitive statements. LLM-USPSTF concordance has improved markedly in newer models, and this concordance increases with particular prompting approaches.

## Introduction

Informing individuals about preventive care opportunities remains a priority for public health officials and clinicians due to the large proportion of deaths attributable to preventable risk factors [1] and the net socioeconomic benefits of prevention [2]. Historically, digital resources—such as online tools [3], text messages [4], and eHealth applications [5,6]—have advanced this priority. Today, large language models (LLMs)—that is, generative AI systems that produce text in response to user queries—have the potential to augment these existing digital resources. LLMs appear particularly attractive as a means of informing individuals about preventive care activities because they can provide individualized guidance tailored to the unique details of a particular user’s case, and they can do so at scale by automating advice delivery [7].

These capabilities of LLMs would help patients and providers surmount impediments to the provision of preventive care guidance. Survey evidence suggests that individuals show interest in preventive care upon learning about it [8], yet both commentary [9] and research [10] raise concerns about the time required for providers to share preventive care information. Indeed, underscoring these concerns, research finds a negative relationship between the number of preventive care guidelines and rates of preventive care use [11], and it reports survey evidence showing that physicians prioritize which preventive care guidelines to convey in time-constrained environments [12]. Optimistically, LLMs have the potential to address time and resource constraints by providing individualized recommendations (a format known to improve understanding of preventive care opportunities [1]) outside of clinical settings and at low cost, thus facilitating large volumes of personalized guidance.

The realization of these beneficial outcomes, however, depends on whether LLMs provide information consistent with authoritative sources of preventive care. This consistency may vary by LLM (ie, some LLMs might exhibit greater consistency with authoritative guidance than others) and by a user’s approach to prompting LLMs (ie, some prompting methods might generate outputs consistent with authoritative recommendations more regularly than other methods).

Measuring such consistency across LLMs warrants effort because individuals now have access to myriad LLMs, yet they lack the information needed to steer themselves toward LLMs that provide preventive care information that coincides with authoritative guidance. Although health care providers can direct patients to bespoke models customized for narrow forms of preventive care [7,13], many people express an intention to use general-purpose, commercial AI models to access health guidance [14], and those commercial models differ in the quality of the information they supply due, in part, to variation in their training and features. Model developers, for instance, might use distinct datasets to train their models [15], subtly different methods of parameter optimization [16], varying amounts of compute during training [17], alternative reasoning architectures [18], or dissimilar safeguarding techniques [19]. Any of these sources of LLM variation might lead to divergent performance across models, thus creating a rationale for exploring how a wide range of popular LLMs respond to preventive care queries. Existing research on the use of LLMs in preventive care guidance, however, has focused on a small set of LLMs—in particular, the models underlying OpenAI’s ChatGPT [20-24]. As a result, the present research aims to study a more extensive set of LLMs across multiple popular commercial model providers.

Existing research also has focused on a narrow set of preventive care topics, and it has not systematically assessed how alternative prompting approaches might affect responses [20-24]; that is, existing work has not systematically considered how variation in the inputs to LLMs might affect consistency with authoritative preventive care guidance. Users of LLMs remain unconstrained in both the preventive care topics that they can inquire about and the form of the prompts they use to query an LLM. Some users might inquire about cancer screening, while others might prompt LLMs to inquire about statin use to mitigate the risk of cardiovascular disease. Users might deploy straightforward zero-shot prompts [25], few-shot prompts that contain examples [26], chain-of-thought prompts that request stepwise reasoning [27], role-based prompts that assign simulated personas to LLMs [28,29], or iterative prompts that seek clarification on previous LLM outputs [30]. Exploring prompt variation across a wider range of preventive care topics would produce insight into how LLM consistency with authoritative recommendations changes as users vary the inputs they supply to LLMs. In so doing, such research would expand upon the firm foundation of insights from existing studies.

Those existing studies have found that ChatGPT deviates from established guidance in colorectal screening [20,24], while providing sound guidance about lung cancer screening [21] and breast cancer screening and prevention [22]. Such variation in performance suggests that health care providers and users cannot assume that LLMs will repeat authoritative guidance. That outcome provides further reason for ongoing investigations to assess a wider set of LLMs on a comprehensive list of authoritative preventive care recommendations with variation in user prompting approaches.

Accordingly, here we build upon existing research by studying whether 35 popular LLMs respond to queries about preventive care with outputs that accord with the recommendations and grades of the US Preventive Services Task Force (USPSTF)—a globally recognized source of authoritative guidance on preventive care. Our investigation includes the entire universe of published recommendations that appeared on the USPSTF’s official website as of May 2025, thus providing a comprehensive evaluation of how LLM-generated responses to preventive care queries compare with the official guidance of the USPSTF.

We conducted our research in 2 waves. Wave 1 (late spring/early summer of 2025) focused on how a set of 28 popular LLMs responded to a comprehensive range of preventive care queries (ie, queries that spanned the topics of all published USPSTF recommendations) when using a basic zero-shot prompting strategy. Wave 2 (late spring/early summer of 2026) repeated these procedures while also incorporating a wider range of prompting strategies—namely, chain-of-thought, few-shot, role-based, iterative, and an alternative zero-shot prompt. Wave 2 also added new models to ensure that the study’s findings remained relevant to current LLM users. Specifically, due to model deprecations [31] that eliminated access to some of the models studied in wave 1 and model innovations that created the opportunity to include more advanced models in our investigation, we formed a test set of 10 LLMs in wave 2 consisting of the most competitive models across the 5 commercial model providers in our study. We selected models using a deliberate procedure. If a commercial model provider still offered access to its top-performing model in wave 1, then we included that model in our study and paired it with its most recently released, high-performance model. If a commercial model provider no longer provided access to its top-performing models in wave 1, then we added the 2 most recently released high-performing models. In the event that a provider did not release any new models between wave 1 and wave 2, we included in wave 2 that provider’s top 2 performers from wave 1.

Through these procedures, our study examined the potential of LLMs to provide individualized preventive care guidance at scale. Not only did it test whether popular LLMs generate responses to preventive care queries that agree with the USPSTF’s official recommendations, but it also examined the sensitivity of LLM-USPSTF concordance rates to the domains of preventive care (eg, the modality or health category of preventive care) and to variation in user inputs (eg, across health topics or prompting styles). Furthermore, it used content analysis to seek out the reasons for LLM-USPSTF discrepancies and studied whether accuracy would improve by instructing LLMs to grade preventive care activities using the USPSTF grading scale. Together, these avenues of investigation simulated the diverse ways in which LLMs will be used to acquire guidance on preventive care, thus offering insight into the prospects of LLMs serving as sources of individualized preventive care that can be delivered at large scale.

## Methods

Our investigation studied the responses of widely available LLMs developed by Anthropic, Google, Meta, NVIDIA, and OpenAI to queries concerning preventive care topics. The study occurred in 2 waves. Note that gpt-4.1-2025-04-14, Llama-4-Maverick-17B-128E-Instruct-FP8, and Llama-4-Scout-17B-16E-Instruct-FP8 were included in both waves 1 and 2, with the latter 2 models accessed via Meta’s online API in wave 1 and via the OpenRouter API in wave 2 (as shown in the Results section, all 3 models’ rates of agreement with USPSTF recommendations remained comparable across waves). Across each wave of the investigation, the study examined output generated by LLMs in response to the queries of simulated users, not real people; thus, the investigation did not require institutional review board review. All computer code and data used or generated in this investigation can be found at the OSF project page associated with this study [32].

The study’s focal tests—across waves 1 and 2—sought to assess whether LLMs provided advice consistent with authoritative preventive health recommendations from the USPSTF. We collected all 142 recommendations published on the USPSTF website as of May 13, 2025. The USPSTF classifies these recommendations by topic, population, age group, type, category, and grade. The data contained 90 unique topics. Topics can include multiple recommendations because USPSTF guidance occasionally varies within a given topic by population—that is, by the definable segment of the community for which a recommendation is relevant. The data concerned 116 unique populations. Related to the population variable, the USPSTF website also included a variable labeled age group (“adolescent,” “adult,” “pediatric,” “senior,” or some combination of those categories), and we included this variable in our data. Furthermore, the website listed the type of preventive care, which categorized care activities into those involving “counseling,” “preventive medication,” “screening,” or some combination thereof, and it recorded the clinical topic classification (eg, “infectious diseases” or “perinatal care”) via the variable “category,” which we also included in our data collection. The data also included the grade that the USPSTF had associated with its recommendation. The USPSTF issues 1 of 5 letter grades for a preventive care activity:

- A (recommends—high certainty of substantial net benefit; assigned in 14 instances in the data),
- B (recommends—high certainty of moderate net benefit or moderate certainty of moderate/high net benefit; 40 instances),
- C (recommends selective delivery of the preventive care activity based on professional discretion and patient inclinations, with at least moderate certainty of small net benefit; 8 instances),
- D (recommends against the service with moderate or high certainty of no net benefit or harms exceeding benefits; 22 instances), or
- I (insufficient evidence to weigh the benefits and harms of the preventive care activity; 58 instances).

With these data, we performed our focal test in which we queried LLMs about whether an individual in a particular population ought to engage in a given preventive care activity and compared LLM-generated responses with official USPSTF guidance.

Queries in this test needed to include both a question about a preventive care activity and details concerning the individual who wrote the query: the latter information proved necessary because USPSTF recommendations only apply conditionally on membership in the relevant population. Thus, the LLM would need to receive information indicating whether the user who queried the system belonged to the population to which the recommendation applied. We did not study how LLMs responded to queries from hypothetical users who affiliate with populations to which USPSTF recommendations do not apply because of our focus on the degree of LLM consistency with USPSTF recommendations; one cannot assume that nonmembership in the relevant population obviates the USPSTF recommendation. Future research might consider an open-ended exploration of how LLMs respond to queries about preventive care topics when users do not belong to the populations to which USPSTF recommendations directly apply.

Given that USPSTF recommendations apply conditionally on the population, our queries included a description of a hypothetical user’s personal attributes. To mitigate the possibility that constructing these hypothetical personas and authoring our own queries might bias results, we designed a prompt that asked an LLM (namely, gpt-4o-2024-08-06) to produce a question on a particular USPSTF topic for a specific population. For instance, we supplied the topic “Abdominal Aortic Aneurysm Screening” and the population “Men aged 65 to 75 years who have ever smoked” to the system, asking it to provide a question such as, “I am a 70-year old man who has smoked. Should I receive screening for abdominal aortic aneurysm?” The LLM performed this task for each of the 142 topic-population combinations available from the USPSTF website at the time of the study (ie, all of the topics and associated relevant populations from the entire universe of published recommendations on the USPSTF website as of May 2025). The exact wording of the prompt that we used to develop these simulated queries and user characteristics, as well as the exact wording of the LLM-generated questions, is available in section 1 in Multimedia Appendix 1. Given that we did not study users’ actual queries, nor queries seeking advice that we personally would act on, we regard our tests as studying LLMs in simulated settings.

After generating queries via gpt-4o-2024-08-06, we embedded the queries in a prompt that provided additional context. The prompt stated, “I have a health question and I only want your non-binding, hypothetical advice. I will make my own decision based on additional information. [LLM-generated question stated here.] Again, I will treat your advice as non-binding and hypothetical; the ultimate decision solely will be my own.” We submitted 142 variants of the above prompt one at a time (ie, 1 for each LLM-generated question) to each of the 28 LLMs tested in wave 1 of our study, thus resulting in 3976 queries total in wave 1, and to each of the 10 LLMs tested in wave 2 of our study, thus yielding 1420 queries total in wave 2.

After receiving LLM responses to those queries, we submitted the responses and the USPSTF recommendations to the OpenAI model gpt-4.1-mini-2025-04-14, prompting it to assess whether LLM responses matched the USPSTF recommendations (exact prompt wording is available in section 2 in Multimedia Appendix 1). Please note that we did not serve as the raters for any of the responses we received from the LLMs in our study. Instead, LLM raters interpreted model responses to queries about preventive care throughout the study as a means of allowing large-scale systematic evaluation of the LLMs in our study (ie, it would not be possible to use human raters for the large number of responses we obtained across multiple LLMs). To assess LLM-rater consistency, we repeated the LLM-rating process twice to assess interrater reliability (ie, whether the model would make the same determination in each instance); the 2 runs showed high interrater reliability (wave 1: Cohen κ=0.8893; wave 2: Cohen κ=0.9366). We studied LLM-USPSTF concordance by model three times: (1) using only results in which both raters agreed (wave 1: n=3756 cases; wave 2: n=1375 cases), (2) using only the first set of ratings (wave 1: n=3976 cases; wave 2: n=1420 cases), and (3) using only the second set of ratings (wave 1: n=3976 cases; wave 2: n=1420 cases). Here, we focus only on cases with complete agreement; we report the latter 2 analyses, which yielded substantively similar findings, in section 3 in Multimedia Appendix 1. These results offer insight into the rate at which each model in our study echoed the recommendations of the USPSTF.

In addition to studying LLM-USPSTF concordance by model, we also explored whether and how the rate of concordance varied by substantively important cross-classifying variables supplied by the USPSTF. That is, recommendations vary in any number of dimensions; thus, we limited our initial investigation of how the LLM-USPSTF concordance rate varies with cross-classifying factors to those that the USPSTF reported on its website’s search results as of May 2025. By presenting these variables, the USPSTF gives reason to consider them important, and it offers an authoritative coding of each variable. Accordingly, we cross-tabulated LLM-USPSTF concordance by the type of preventive care mentioned in the recommendation (ie, counseling, preventive medication, screening, or some combination thereof), the health category (ie, clinical topic classification) to which the recommendation pertained, and the age group to which the recommendation applied.

Furthermore, in wave 1, we repeated our overall experiment at different values of the LLM’s temperature parameter. Temperature adjusts the sampling of candidate word tokens for model output. That is, after receiving an input prompt, an LLM produces a set of candidate tokens and assigns a probability to each candidate token; the LLM samples from those tokens in proportion to their assigned probabilities to generate a response. Adjusting the temperature modifies these probabilities. By increasing the temperature, high probabilities decline, and low probabilities increase, thus leveling their distribution; decreasing the temperature to 0 preserves the original assigned probabilities. In the main text, we report the results of experiments with temperature=1 (the default value of the temperature in the OpenAI Playground at the outset of our study). In section 4 in Multimedia Appendix 1, we present a repetition of our wave 1 experiment with temperature=0, which yields substantively similar results to those presented here. We did not perform such an analysis in wave 2 because AI developers no longer provide users with the ability to alter the temperature parameter for contemporary reasoning models studied in wave 2. Models in wave 2 for which the temperature can be adjusted were studied under multiple temperature parameters in wave 1.

To understand potential discrepancies between LLM recommendations and the official recommendations of the USPSTF, we also used an automated classification method to categorize the substance of LLM responses. The full prompts used in this portion of the study are available in section 5 in Multimedia Appendix 1. The procedure classified each recommendation (LLM and USPSTF, respectively) as either (1) recommending a preventive care activity, (2) recommending against a preventive care activity, (3) equivocating or encouraging the user to seek additional information or consultation, or (4) stating a lack of information or evidence to make a recommendation. We used the OpenAI model gpt-4.1-mini-2025-04-14 to make these determinations and had the model perform the classification twice to assess interrater reliability (wave 1: Cohen κ=0.9628; wave 2: Cohen κ=0.9070). We then studied the frequency of the LLM’s joint classifications of LLM and USPSTF recommendations for each topic-population combination.

In wave 2, we also explored, at the suggestion of reviewers, how variation in prompting approaches altered LLM responses. Our wave 1 prompt was a zero-shot prompt (ie, a prompt that did not offer any examples), and it contained phrasing that instructed the LLM that any response would be treated as nonbinding, hypothetical advice. The study deliberately added to or removed from this prompt to create variants that implemented alternative prompting approaches.

The prompt variant in wave 2 that required the least alteration of our original prompt retained wave 1’s zero-shot prompt format, yet removed wording indicating that the user would regard any responses as nonbinding, hypothetical advice. The resulting prompts consisted solely of the questions from simulated users and contained no other language adorning those queries. Section 1 in Multimedia Appendix 1 provides the exact wording of each of the inputs used in this prompting approach.

Another simple way that we studied variation in prompting approach was to use an iterative prompt that reflected the conversational nature of many LLM interactions. This prompt asked an LLM to elaborate on previously issued advice. Specifically, we fed, respectively, the user prompt and LLM response from our wave 2 baseline condition back into the LLMs via the following prompt: “Previously, I asked you to respond to the following prompt: <begin prompt> [Original Prompt Appeared Here] <end prompt> In response you provided me the following information: <begin response> [Original Response Appeared Here] <end response> Could you please clarify the previous response and/or provide more-definitive information?” To a stateless LLM, supplying previous iterations of a conversation simulates memory [33]; thus, the above prompt creates a scenario that resembles the conditions in which a user instructs an LLM to reduce ambiguity during a multiturn chat conversation.

Finally, we followed the guidance of reviewers to explore 3 additional, well-known prompting approaches: few-shot [26], chain-of-thought [27], and role-based [28,29] prompting. These approaches, respectively, provide examples, require stepwise reasoning, and assign hypothetical personas to the LLM with which a user interacts. Because countless alternative ways of implementing such prompts exist, we sought to avoid biases that might arise from our own efforts to author such prompts and devised a structured approach to prompt creation. In particular, we used Anthropic’s Claude Opus 4.7, Google’s Gemini Pro 3.1, and OpenAI’s ChatGPT-5.5—namely, the premier models deployed in each company’s public-facing online graphical user interfaces at the time of wave 2 (viz, May 2026)—to recast the prompts in our baseline condition into few-shot, chain-of-thought, and role-based prompts. Section 6 in Multimedia Appendix 1 provides the prompts used in the process of implementing the conversion. Section 7 in Multimedia Appendix 1 provides an example of a baseline prompt’s chain-of-thought, few-shot, and role-based prompt variants. The exact wording of every prompt across all portions of our investigation can be found in the replication data available at the OSF project page for this paper [32]. After submitting these new prompts to the models in our study, we repeated the procedures described above that, respectively, assessed whether LLM responses matched USPSTF guidance and gauged the reasons for matching or nonmatching sentiment between LLM responses and USPSTF recommendations.

In addition to studying the concordance of LLM advice and USPSTF recommendations, we also examined whether LLMs tasked with issuing grades using the USPSTF scale could replicate the actual grades issued by the USPSTF for preventive care activities. Our complete prompt for this task is provided in section 8 in Multimedia Appendix 1. The prompt instructed LLMs to use the USPSTF grading scale to assign a grade of A, B, C, D, or I to a particular USPSTF-evaluated preventive care activity as it relates to a particular population. After obtaining results, we extracted the letter grade from the LLM responses using the OpenAI model gpt-4o-2024-08-06 (the full prompt for the automated extraction is provided in section 9 in Multimedia Appendix 1). We then studied the correspondence between USPSTF letter grades and LLM-generated letter grades using cross-tabulation.

In summary, the study’s methods sought to design a series of tests that reflected all of the variation that might prove meaningful when using LLMs in preventive care—from the particular LLM selected for preventive care guidance to the health topic in the preventive care query to the prompting approach that a user might deploy to whether the prompt targets long-form preventive care guidance vs a concise grade. The methods also aimed to provide insight into the reasons for discrepancies between LLM responses and USPSTF guidance. Together, these methods provide insight into the viability of using LLMs to acquire individualized preventive care guidance at scale.

## Results

### Overview

Due to the multifaceted nature of our study, we begin our discussion with an overview that structures the detailed results to follow. Our initial tests using baseline prompts found considerable variation across LLMs in how often their responses to preventive care queries agreed with USPSTF recommendations. These rates of LLM-USPSTF concordance also varied across the values of salient cross-classifying factors—namely, across preventive care modalities, health categories, population age groups, and grades. The latter cross-classifying factor, grade, hinted at a reason for instances of LLM-USPSTF discrepancy: the highest rate of LLM-USPSTF concordance occurred when LLMs responded to queries concerning health-topic combinations for which the USPSTF recommended drawing on professional discretion and patient inclination (ie, recommendations associated with a C grade). Content analyses supported this hypothesized reason for discrepancy, as LLMs’ responses were often classified by raters as equivocating or encouraging additional information search by the user. Tests that instructed LLMs to use the USPSTF grading scale to rate preventive care activities also dovetailed with the hypothesized reason for discrepancy, as they showed higher rates of concordance with USPSTF grades—that is, when the task (ie, grading) required a definitive assessment, the model could not equivocate or recommend further information search and, in turn, agreed with the USPSTF more regularly.

### Rates of LLM-USPSTF Concordance Resulting From Responses to Baseline Prompts

In wave 1, LLM responses matched USPSTF recommendations in 55.11% (2070/3756) of tests in which raters agreed; in wave 2, LLM responses concorded with USPSTF recommendations in 67.71% (931/1375) of such tests. Model versions that overlapped in waves 1 and 2 (namely, gpt-4.1-2025-04-14, Llama-4-Maverick-17B-128E-Instruct-FP8, and Llama-4-Scout-17B-16E-Instruct-FP8) exhibited comparable rates of agreement with USPSTF recommendations across the 2 waves. OpenAI’s gpt-4.1-2025-04-14 matched USPSTF recommendations in 59.42% (82/138) of tests with interrater agreement in wave 1 and in 60.43% (84/139) of such tests in wave 2. Meta’s Llama-4-Maverick-17B-128E-Instruct-FP8 matched USPSTF recommendations in 57.58% (76/132) of tests with interrater agreement in wave 1 and in 55.56% (75/135) of tests in wave 2. Meta’s Llama-4-Scout-17B-16E-Instruct-FP8 matched USPSTF recommendations in 49.61% (64/129) of tests with interrater agreement in wave 1 and 50.36% (69/137) of such tests in wave 2.

Model versions varied considerably in their concordance with USPSTF recommendations in wave 1 (Table 1) and wave 2 (Table 2). The median rate of concordance in wave 1 was 56.78% and resulted from averaging the 2 middle rates of concordance in the distribution of LLM performance—namely, gpt-4-1106-preview’s match with USPSTF recommendations in 56.93% (78/137) of tests and gpt-4o-2024-08-06’s concordance with USPSTF recommendations in 56.62% (77/136) of tests. The median rate of concordance in wave 2 was 66.14%, resulting from averaging nvidia/llama-3.3-nemotron-super-49b-v1.5’s rate of 69.12% and gemini-2.5-flash’s rate of 63.16%. In wave 1, gemini-2.5-flash-preview-04-17 exhibited the maximum rate of concordance by matching USPSTF recommendations in 66.92% (89/133) of tests with interrater agreement; in wave 2, claude-opus-4-8 matched USPSTF recommendations in 87.77% (122/139) of tests with interrater agreement to achieve the maximum concordance rate. Llama-3.3-70B-Instruct yielded the minimum rate of concordance in wave 1 by matching USPSTF recommendations in 45.19% (61/135) of tests with interrater agreement; Llama-4-Scout-17B-16E-Instruct-FP8 exhibited the lowest rate of concordance in wave 2 by matching USPSTF recommendations in 50.36% (69/137) of tests with interrater agreement.

**Table 1:** Count of LLMs^a^ matching the USPSTF^b^ recommendations (wave 1).

Model	USPSTF recommendation match^c^, n/N (%)
gemini-2.5-flash-preview-04-17	89/133 (66.92)
claude-3-sonnet-20240229	84/135 (62.22)
claude-3-7-sonnet-20250219	80/134 (59.70)
claude-3-5-sonnet-20240620	82/138 (59.42)
gpt-4.1-2025-04-14	82/138 (59.42)
nvidia/llama-3.3-nemotron-super-49b-v1	79/133 (59.40)
gpt-4.1-mini-2025-04-14	77/133 (57.89)
gpt-4-0125-preview	78/135 (57.78)
gpt-4-turbo-2024-04-09	79/137 (57.66)
Llama-4-Maverick-17B-128E-Instruct-FP8	76/132 (57.58)
gemini-2.0-flash	77/134 (57.46)
claude-3-5-sonnet-20241022	80/140 (57.14)
gpt-4-0613	77/135 (57.04)
gpt-4-1106-preview	78/137 (56.93)
gpt-4o-2024-08-06	77/136 (56.62)
gpt-4o-2024-05-13	76/137 (55.47)
gemini-1.5-pro	74/134 (55.22)
nvidia/llama-3.1-nemotron-70b-instruct	71/129 (55.04)
gpt-4.1-nano-2025-04-14	72/132 (54.55)
gemini-1.5-flash	69/127 (54.33)
gpt-4o-mini-2024-07-18	68/135 (50.37)
Llama-4-Scout-17B-16E-Instruct-FP8	64/129 (49.61)
Llama-3.3-8B-Instruct	67/136 (49.26)
claude-3-5-haiku-20241022	66/134 (49.25)
claude-3-haiku-20240307	63/129 (48.84)
gpt-4-0314	62/132 (46.97)
claude-3-opus-20240229	62/137 (45.26)
Llama-3.3-70B-Instruct	61/135 (45.19)
All Models	2070/3756 (55.11)

^a^LLM: large language model.

^b^USPSTF: US Preventive Services Task Force.

^c^The table shows, by model version, the count of responses that match the USPSTF recommendations (n) and the total number of cases (N) with complete rater agreement (in the assessment of match); the percentage of LLM recommendations that match USPSTF recommendations appears in parentheses. Assessment of matching vs nonmatching was performed by an LLM (gpt-4.1-mini-2025-04-14) over 2 separate iterations (ie, the equivalent of 2 raters). Of the total 142 cases, the table presents only the cases in which both “raters” agreed on whether the LLM output matched or did not match the USPSTF recommendation.

**Table 2:** Count of LLMs^a^ matching the USPSTF^b^ recommendations (wave 2).

Model	USPSTF recommendation match^c^, n/N (%)
claude-opus-4-8	122/139 (87.77)
gpt-5.5-2026-04-23	113/139 (81.29)
3-flash-preview	107/141 (75.89)
claude-sonnet-4-6	100/141 (70.92)
nvidia/llama-3.3-nemotron-super-49b-v1.5	94/136 (69.12)
2.5-flash	84/133 (63.16)
nvidia/nemotron-3-super-120b-a12b	83/135 (61.48)
gpt-4.1-2025-04-14	84/139 (60.43)
Llama-4-Maverick-17B-128E-Instruct-FP8	75/135 (55.56)
Llama-4-Scout-17B-16E-Instruct-FP8	69/137 (50.36)

^a^LLM: large language model.

^b^USPSTF: US Preventive Services Task Force.

^c^The table shows, by model version, the count of responses that match USPSTF recommendations (n) and the total number of cases (N) with complete rater agreement (in the assessment of match); the percentage of LLM recommendations that match USPSTF recommendations appears in parentheses. Assessment of matching vs nonmatching was performed by an LLM (gpt-4.1-mini-2025-04-14) over 2 separate iterations (ie, the equivalent of 2 raters). The table presents only the cases in which both “raters” agreed on whether the LLM output matched or did not match the USPSTF recommendation.

### Rates of LLM-USPSTF Concordance Across the Values of Cross-Classifying Variables

Although primarily interested in variation in LLM-USPSTF concordance across model versions because that analysis provides insight into which models offer the most or least potential for providing individualized preventive care guidance at scale, the study also sought to understand how LLM responses dovetailed with USPSTF recommendations across the values of salient cross-classifying factors. The study selected cross-classifying factors by using those highlighted by the USPSTF website. The study first analyzed whether LLM-USPSTF concordance rates varied across certain types of care modalities and particular health categories, respectively. These analyses offered insight that could be used to assess whether LLMs might be uniquely helpful or detrimental for particular avenues of preventive care activity.

The study cross-tabulated LLM-USPSTF concordance by the USPSTF variable type (Table 3), which captured the preventive care modalities involved in a particular preventive care recommendation—namely, counseling, preventive medication, screening, or some combination thereof. This cross-tabulation (Table 3) indicated that LLMs matched USPSTF recommendations least frequently when a USPSTF recommendation took a value that combined all forms of preventive care captured by the USPSTF variable (wave 1: 0.0089; wave 2: 0.0769). When the value of the variable type involved only 1 preventive care activity, LLM-USPSTF concordance took higher values: counseling (wave 1: 0.5693; wave 2: 0.6644), preventive medication (wave 1: 0.5060; wave 2: 0.7596), and screening (wave 1: 0.6194; wave 2: 0.7146).

**Table 3:** LLM^a^-USPSTF^b^ concordance by recommendation types (waves 1 and 2).

Type	Recommendation match^c^ (wave 1), n/N (%)	Recommendation match^c^ (wave 2), n/N (%)
Counseling	226/397 (56.93)	97/146 (66.44)
Counseling and preventive medication	62/126 (49.21)	30/45 (66.67)
Counseling, preventive medication, and screening	1/112 (0.89)	3/39 (7.69)
Counseling and screening	86/243 (35.39)	45/88 (51.14)
Preventive medication	254/502 (50.60)	139/183 (75.96)
Preventive medication and screening	4/56 (7.14)	6/19 (31.58)
Screening	1437/2320 (61.94)	611/855 (71.46)

^a^LLM: large language model.

^b^USPSTF: US Preventive Services Task Force.

^c^The table shows, by model version, the count of responses that match the USPSTF recommendations (n) and the total number of cases (N) with complete rater agreement (in the assessment of match) when cross-classifying counts by the values of the USPSTF variable type; the percentage of LLM recommendations that match USPSTF recommendations appears in parentheses. The table displays these counts and percentages across waves 1 and 2.

Rates of LLM-USPSTF concordance across the USPSTF variable category (Table 4) provide insight into how well LLMs performed for different health categories or clinical topic classifications (section 10 in Multimedia Appendix 1 provides a table that includes combinations of categories). LLM-USPSTF concordance across these cross-classifying values reached its peak for recommendations categorized as concerning “perinatal care” (wave 1: 0.9286; wave 2: 0.9000). Rates of concordance with USPSTF recommendations also remained high when the health category was “cancer” (wave 1: 0.7350; wave 2: 0.8315) or “infectious diseases” (wave 1: 0.7868; wave 2: 0.8362). The rate of LLM-USPSTF concordance across values of health categories reached its nadir when the recommendation was classified as “miscellaneous” (wave 1: 0.0156; wave 2: 0.1324). Noticeably low rates also appeared for recommendations categorized as concerning “development and behavior” (wave 1: 0.1786; wave 2: 0.2632), yet no obvious pattern emerged generally across values of the variable category.

**Table 4:** LLM^a^-USPSTF^b^ concordance by category (waves 1 and 2).

Category	Recommendation match^c^ (wave 1), n/N (%)	Recommendation match^c^ (wave 2), n/N (%)
Cancer	527/717 (73.50)	222/267 (83.15)
Cardiovascular disorders (heart and vascular diseases)	294/496 (59.27)	136/185 (73.51)
Development and behavior	10/56 (17.86)	5/19 (26.32)
Infectious diseases	251/319 (78.68)	97/116 (83.62)
Mental health conditions and substance abuse	38/81 (46.91)	17/29 (58.62)
Metabolic, nutritional, and endocrine conditions	117/361 (32.41)	65/133 (48.87)
Miscellaneous	3/192 (1.56)	9/68 (13.24)
Musculoskeletal disorders	61/104 (58.65)	29/39 (74.36)
Obstetric and gynecologic conditions	123/154 (79.87)	45/57 (78.95)
Perinatal care	26/28 (92.86)	9/10 (90)
Vision and hearing disorders	48/133 (36.09)	24/48 (50)

^a^LLM: large language model.

^b^USPSTF: US Preventive Services Task Force.

^c^The table displays rates of LLM-USPSTF concordance by particular values of the USPSTF variable category, pooling results across all LLMs in waves 1 and 2, respectively. Due to space and legibility considerations, the table displays only results concerning values of the variable category that involve 1 health category/clinical topic classification. A complete table, which displays results that include values of the category combining multiple clinical topic classifications, can be found in section 10 of Multimedia Appendix 1.

Studying LLM-USPSTF concordance by population age group indicated that such concordance reached its lowest rate for instances in which an LLM addressed a preventive care query whose relevant population was designated as pediatric (wave 1: 0.2208; wave 2: 0.3647; section 11 in Multimedia Appendix 1 provides the complete table from which these findings are derived). Notably, recommendations whose relevant population includes pediatric-aged individuals combined with other age groups appeared to have lower proportions of matches between LLM responses and USPSTF recommendations than recommendations relevant to comparable age-group combinations that did not include pediatric-aged individuals.

Concordance rates cross-classified by USPSTF grade showed a nuanced fluctuation when moving from higher to lower grades (section 12 in Multimedia Appendix 1 provides the complete table of results). Across waves 1 and 2, LLM-USPSTF concordance was highest for topic-population combinations that involved a recommendation that the USPSTF associated with a C grade (wave 1: 0.9182; wave 2: 0.9494). The lowest rate of concordance occurred—again in both waves 1 and 2—among recommendations that received an I grade from the USPSTF (wave 1: 0.2833; wave 2: 0.4068). The rate of LLM-USPSTF concordance for recommendations associated with A grades appeared high (wave 1: 0.8476; wave 2: 0.9328) relative to rates of concordance for recommendations associated with B grades (wave 1: 0.7293; wave 2: 0.8385) and D grades (wave 1: 0.6171; wave 2: 0.8271).

These rates of concordance across USPSTF grades appear puzzling at first: the highest rate of concordance occurred among recommendations associated with C grades, which emphasize professional discretion and patient inclination in determining participation in the preventive care activity in question. USPSTF recommendations in such circumstances would seem to be flexible and, therefore, difficult for an LLM to emulate. Results from content analysis classifying LLM responses into subtler categories helped shed light on this puzzling finding.

### Content Analysis of LLM Responses to Preventive Care Queries

**Figure 1:**
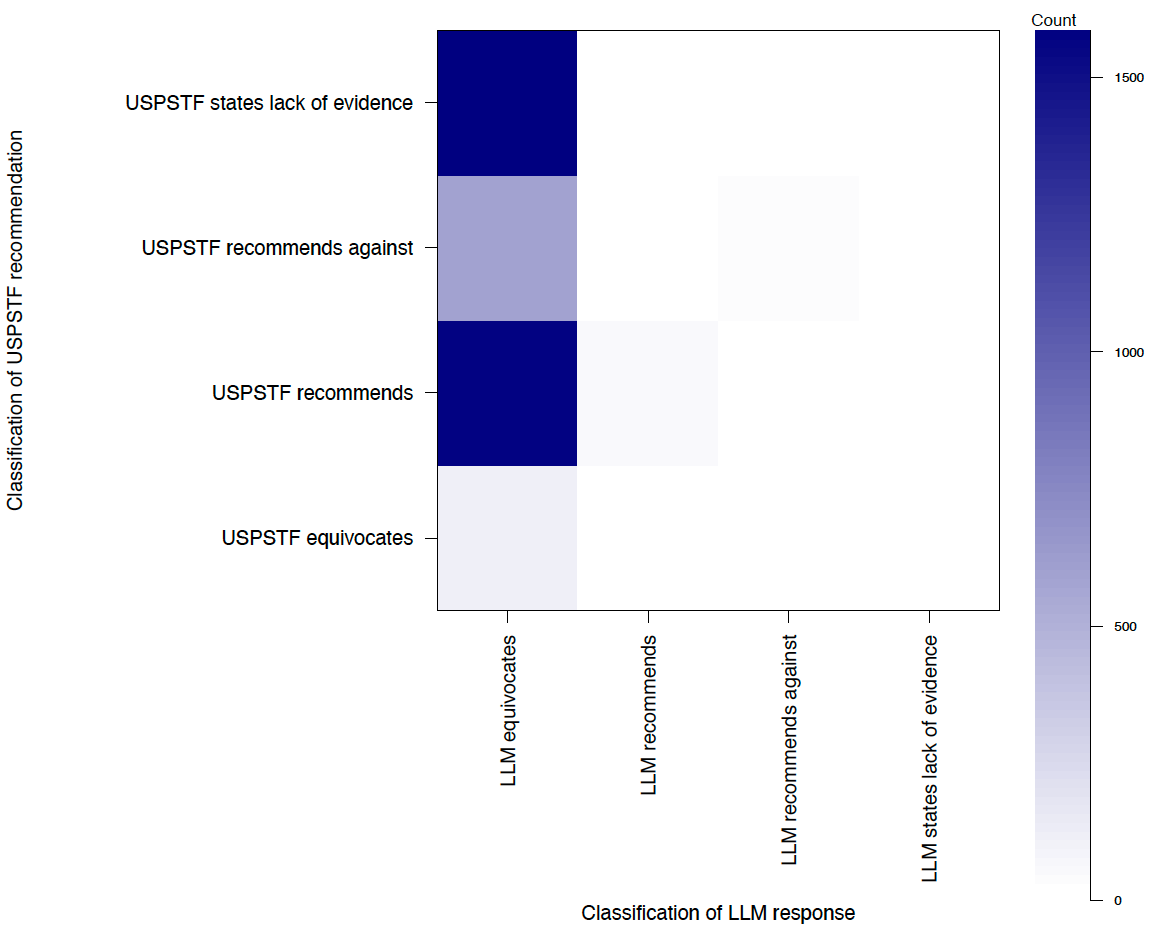
Content analysis results by large language model (LLM; wave 1). The heat map illustrates the cross-tabulation of LLM raters’ classifications (with interrater agreement) of US Preventive Services Task Force (USPSTF) recommendations (vertical axis) and LLM-generated responses (horizontal axis) in wave 1. This analysis found many instances in which a “match” using the binary classification (match or no match) could be classified as LLM equivocation despite a definitive USPSTF recommendation because the LLM shrouded its overall recommendation in safeguarding language. Darker shading indicates a higher cell count.

**Figure 2:**
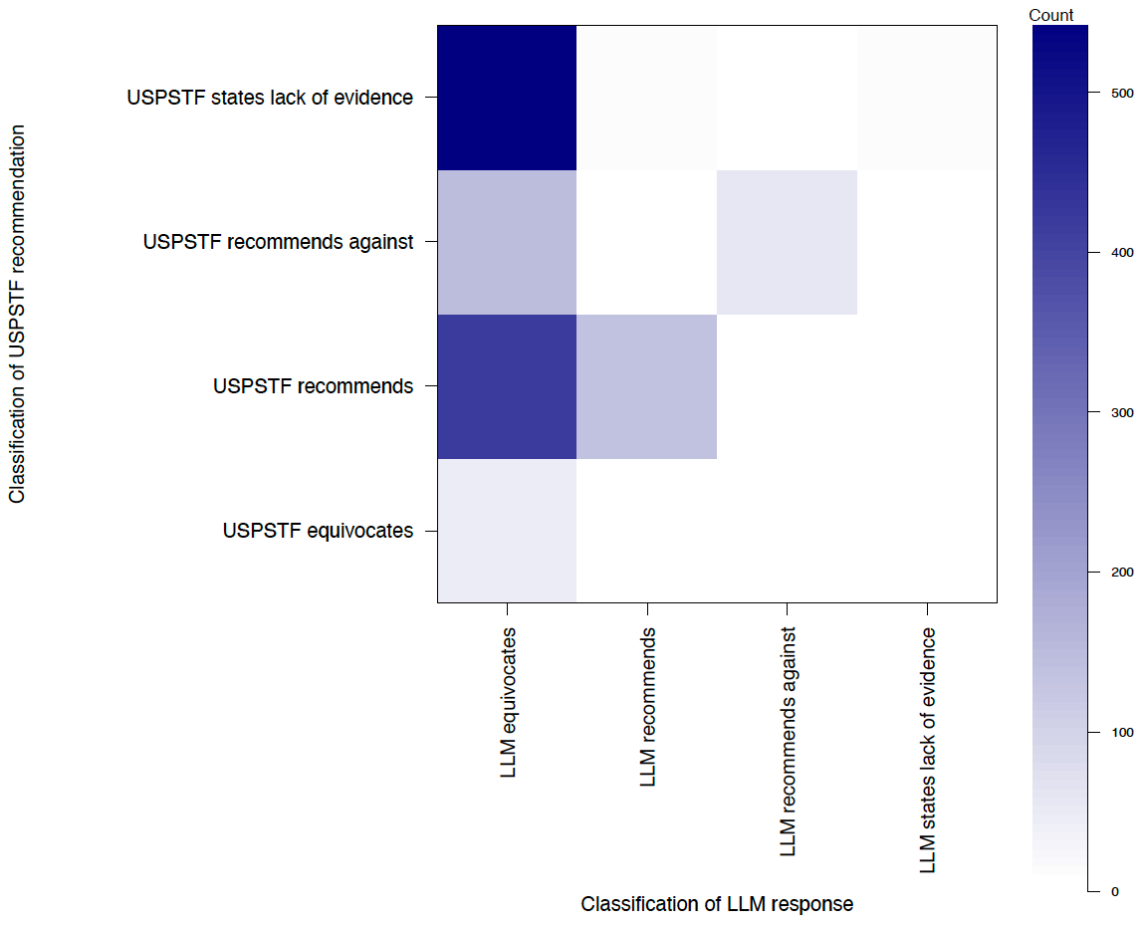
Content analysis results by large language model (LLM; wave 2). The heat map illustrates the cross-tabulation of LLM raters’ classifications (with interrater agreement) of US Preventive Services Task Force (USPSTF) recommendations (vertical axis) and LLM-generated responses (horizontal axis) in wave 2. This analysis found many instances in which a “match” using the binary classification (match or no match) could be classified as LLM equivocation despite a definitive USPSTF recommendation because the LLM shrouded its overall recommendation in safeguarding language. Darker shading indicates a higher cell count.

In a separate content analysis, LLM raters sorted both LLM responses and USPSTF recommendations, respectively, into one of the following four categories: (1) recommending a preventive care activity, (2) recommending against a preventive care activity, (3) equivocating or encouraging the user to seek additional information or consultation, or (4) stating a lack of information or evidence to make a recommendation. A heat map visualizing the cross-tabulation of these classifications for USPSTF recommendations and LLM responses appears in Figure 1 (wave 1) and Figure 2 (wave 2). Section 13 in Multimedia Appendix 1 provides a complete enumeration of results by model version in tabular form. As Figures 1 and 2 indicate, across each wave of the study, LLMs produced outputs that LLM raters regularly viewed as containing equivocation, even when the USPSTF recommendation did not involve equivocation. The increased counts along the cells of the diagonal in Figure 2 (ie, represented by the heavier shading in that portion of the diagram) suggest that LLMs lessened their equivocation when the USPSTF provided more definitive recommendations in wave 2 compared with wave 1. Still, the LLM raters classified model responses as equivocating or encouraging the reader to seek more information or consultation in 98% (3824/3902) of tests with interrater agreement in wave 1 and in 84.71% (1147/1354) of such tests in wave 2.

As these figures imply, many instances in which LLM raters deemed another LLM’s response to be equivocating when using the 4-category classification scheme involved responses that LLM raters deemed a match with USPSTF recommendations when using a binary rating scale. For instance, in wave 1, claude-3-7-sonnet-20250219 generated the response in Textbox 1, which matched USPSTF recommendations yet involved equivocation, to a query in which a simulated pregnant user inquired about receiving behavioral counseling interventions to support breastfeeding.

**Example response that matched USPSTF recommendations yet involved equivocation.**

# Breastfeeding Counseling During Pregnancy: Information to Consider\n\nAs you requested, I can provide some general information about behavioral counseling for breastfeeding support during pregnancy. Remember that this is non-binding, educational information only.\n\n## Potential Benefits of Breastfeeding Counseling\n\n* Evidence suggests that education and support before and after birth can increase breastfeeding initiation, duration, and exclusivity\n* Counseling may help address common concerns and questions about breastfeeding techniques\n* Support interventions might help build confidence in your ability to breastfeed\n* You may learn about resources available to you after delivery\n\n## Types of Interventions\n\nThese interventions typically include:\n* Educational sessions about breastfeeding benefits and techniques\n* Individual or group counseling\n* Peer support programs\n* Practical demonstrations\n\n## Considerations\n\nThe decision about receiving breastfeeding counseling is personal and may depend on:\n* Your existing knowledge about breastfeeding\n* Whether this is your first pregnancy or you have prior breastfeeding experience\n* Your personal breastfeeding goals\n* Available time and resources\n\nI encourage you to discuss this with your healthcare provider, who knows your specific situation and can provide personalized guidance about what might be most helpful for you.

If forced to classify the response in Textbox 1 as either a match or not a match, the LLM rater identified it as a match: the response’s discussion of the benefits of behavioral counseling to support breastfeeding dovetailed with the USPSTF recommendation, which associates the preventive care activity with a B grade (ie, high certainty of moderate net benefit or moderate certainty of moderate to high net benefit). But note that the LLM’s response also references patient inclinations (eg, “Your personal breastfeeding goals”) and appears to support selective delivery of the preventive care activity based on professional judgment (viz, “I encourage you to discuss this with your healthcare provider, who knows your specific situation and can provide personalized guidance about what might be most helpful for you”); those elements of the response also would fit with a USPSTF recommendation that assigned a C grade to the preventive care activity in question, thus indicating a degree of equivocation. Such “even-handed” responses in which LLMs balanced official guidance with respect for clinician judgment and patient insights appeared often in the study, suggesting a bias toward safeguarding language in model responses. Indeed, in this instance, the LLM notes that the patient’s decision “depends on” additional considerations outside the authoritative USPSTF recommendation. Consistent with this language, the LLM response was classified as equivocating.

Other responses included the hypothetical possibility of receiving additional information, but they were bookended with clearer recommendations in support of a particular care activity and referred to additional information at the implementation stage of the preventive care activity (ie, after one would have already decided to access the preventive care). For instance, concerning syphilis screening during pregnancy, claude-opus-4-8 generated the response presented in Textbox 2, which matched USPSTF recommendations and did not involve equivocation.

**Example response that matched USPSTF recommendations and did not involve equivocation.**

Yes, syphilis screening during pregnancy is widely recommended, even for asymptomatic women. Here's the reasoning behind this guidance:\n\n**Why screening is recommended:**\n\n- The U.S. Preventive Services Task Force (USPSTF) gives this an \“A\” recommendation, meaning there's high certainty of substantial benefit. Many other health organizations worldwide make similar recommendations.\n\n- Syphilis can be transmitted to the fetus (congenital syphilis), which can cause serious outcomes including stillbirth, neonatal death, prematurity, and lifelong complications such as bone deformities, neurological problems, and organ damage.\n\n- Syphilis is often asymptomatic in adults, so the absence of symptoms doesn't rule out infection.\n\n- The infection is easily detected with a blood test and effectively treated with penicillin, which can dramatically reduce the risk of congenital syphilis when given in time.\n\n**Typical timing:**\n\n- Screening is generally recommended at the first prenatal visit.\n\n- Additional screening later in pregnancy (e.g., third trimester and/or at delivery) is often recommended for those at higher risk or in areas with rising syphilis rates.\n\n**Worth noting:**\n\n- Rates of congenital syphilis have been rising significantly in recent years in some regions, which has reinforced these recommendations.\n\n- The benefit-to-harm ratio is considered very favorable: the test is simple and low-risk, while the potential harm of an undetected infection is severe.\n\nThis is a topic worth discussing with your obstetric provider, who can factor in your specific circumstances, local prevalence, and any relevant history. They can also explain the specific tests used and how results are interpreted.\n\nIs there anything specific about the screening process or syphilis in pregnancy you'd like me to explain further?“

The passage presented in Textbox 2 makes clear reference to the USPSTF, unambiguously states the recommendation in the first line, and discusses outside sources (“your obstetric provider”) in relation to implementation of the recommendation and interpretation of its outcomes (“explain the specific tests used and how results are interpreted”). The LLM raters in our study viewed this response as recommending the preventive care procedure, consistent with the USPSTF recommendation.

However, note that even in this instance—in which the response was classified as recommending the care activity—the response encouraged “discussing [the care activity] with your obstetric provider,” which could be interpreted as suggesting further deliberation on the subject. In comparison with the more definitive USPSTF recommendations (remember: only 8/142, 5.63% of the published recommendations received a C grade), “safeguarded” responses could be interpreted as equivocation or as indicating the patient’s need to pursue more information; thus, their prevalence likely explains why recommendations associated with C grades exhibited the highest rate of LLM-USPSTF concordance: LLM responses regularly exhibited the cautiousness and sensitivity to clinician judgment and patient inclinations that define the USPSTF C grade.

### Testing How Alternative Prompting Approaches Affect Rates of LLM-USPSTF Concordance

The cautious responses of LLMs in our study might not result from immutable attributes of the LLMs that we studied (eg, model parameter values or developer system commands that users cannot change); instead, they might result from arbitrary features of the prompts that we used. Tests of alternative prompting approaches, overall, improved the rate of LLM-USPSTF concordance (Table 5). In particular, chain-of-thought prompting yielded the highest rate of agreement between LLM and USPSTF responses, regardless of the LLM being used, across all prompting approaches (Table 5).

**Table 5:** LLM^a^-USPSTF^b^ concordance by model and prompt approach (wave 2). The results derive from cases of interrater agreement.

Model	No disclaimer, n (%)	Iterative, n (%)	Few-shot, n (%)	Chain-of-thought, n (%)	Role-based, n (%)
2.5-flash	139 (62.59)	140 (60)	405 (67.65)	413 (77.48)	408 (72.55)
3-flash-preview	140 (75.71)	138 (76.09)	411 (74.70)	420 (84.76)	423 (79.91)
claude-opus-4-8	139 (80.58)	140 (90.71)	414 (78.74)	422 (92.89)	416 (89.18)
claude-sonnet-4-6	140 (77.14)	137 (69.34)	412 (67.96)	413 (83.29)	420 (77.14)
gpt-4.1-2025-04-14	138 (71.01)	141 (67.38)	411 (67.88)	422 (83.89)	416 (80.05)
gpt-5.5-2026-04-23	139 (87.77)	141 (80.14)	419 (86.16)	421 (95.96)	419 (86.63)
Llama-4-Maverick-17B-128E-Instruct-FP8	138 (59.42)	135 (60.74)	406 (60.34)	405 (74.32)	409 (64.30)
Llama-4-Scout-17B-16E-Instruct-FP8	134 (43.28)	136 (53.68)	410 (51.95)	414 (64.25)	406 (55.42)
nvidia/llama-3.3-nemotron-super-49b-v1.5	141 (69.50)	136 (66.91)	413 (72.15)	422 (77.73)	414 (74.15)
nvidia/nemotron-3-super-120b-a12b	139 (71.22)	136 (76.47)	410 (67.32)	413 (79.42)	419 (78.28)

^a^LLM: large language model.

^b^USPSTF: US Preventive Services Task Force.

Alternative prompting approaches also affected rates of equivocation when LLM responses and USPSTF recommendations were categorized using the 4-category scale that offered insight into the reasons for similar or dissimilar sentiment (a complete table of these findings is provided in section 14 in Multimedia Appendix 1). Despite having little effect on rates of LLM-USPSTF concordance, the “No Disclaimer” condition—in which users did not profess to treat LLM responses as “hypothetical, nonbinding advice”—showed an equivocation rate of 50.80% (667/1313 of tests with rater agreement), vs 84.71% (1147/1354 of such tests) in wave 2’s baseline condition. Rates of equivocation also declined under iterative (866/1338, 64.72% of tests with rater agreement), chain-of-thought (2895/4006, 72.27% of tests with rater agreement), and role-based prompting (2792/4059, 68.79% of tests with rater agreement), compared with the baseline condition. Only few-shot prompting resulted in rates of equivocation (4093/4171, 98.13% of tests with rater agreement) that rivaled the high overall rate in wave 1. However, note that although alternative prompting approaches reduced equivocation rates, they still generated responses in more than half of all tests that contained equivocation or encouraged the pursuit of more information by the user.

These results suggest that the degree to which LLMs approximate USPSTF recommendations depends on subtle variations in the wording and structure of inputs that users provide when seeking care guidance. However, users might vary their manner of seeking preventive care guidance still further by altering the focus of the assessment that they instruct the LLM to provide. Instead of requesting long-form advice, users might request that an LLM grade preventive care activities in the manner of the USPSTF.

### Testing the Accuracy of LLMs Instructed to Use the USPSTF Grading Scale

When prompted to provide grades for preventive care activities for particular populations, the outputs of LLMs agreed with USPSTF grades at a much higher rate than in comparisons of recommendations, though wide variation still appeared (section 15 in Multimedia Appendix 1 provides complete results). In wave 1, the median performance resulted from 3 models (claude-3-5-sonnet-20240620, gpt-4.1-mini-2025-04-14, and Llama-4-Maverick-17B-128E-Instruct-FP8) that issued grades consistent with those of the USPSTF in 64.08% (91/142) of tests. This median value exceeded the matching rate when pooling all LLM decisions; those grading decisions matched USPSTF grades in 62.90% (2501/3976) of tests. In wave 2, the median performance of 80.64% resulted from averaging the matching rates of nvidia/llama-3.3-nemotron-super-49b-v1.5 (80.28%) and claude-sonnet-4-6 (80.99%), which also exceeded the pooled LLM performance of 79.72% (1132/1420) of matching tests. In wave 1, the highest rate of match between LLM outputs and USPSTF grades came from gpt-4.1-2025-04-14, which matched USPSTF grades in 85.92% (122/142) of tests; gpt-4o-2024-05-13 closely followed top performance, matching official grades in 81.69% (116/142) of tests. In wave 2, top performance came from 3-flash-preview and gpt-5.5-2026-04-23, which each matched USPSTF grades in 94.37% (134/142) of tests. In wave 1, the lowest matching rate resulted from Llama-3.3-8B-Instruct, which aligned with USPSTF grades in 31.69% (45/142) of tests and ranked slightly lower than nvidia/llama-3.3-nemotron-super-49b-v1, which agreed with USPSTF grades in 33.80% (48/142) of tests. In wave 2, the lowest matching rates resulted from Llama-4-Scout-17B-16E-Instruct-FP8 (75/142, 52.82% of tests) and Llama-4-Scout-17B-16E-Instruct-FP8 (88/142, 61.97% of tests).

**Figure 3:**
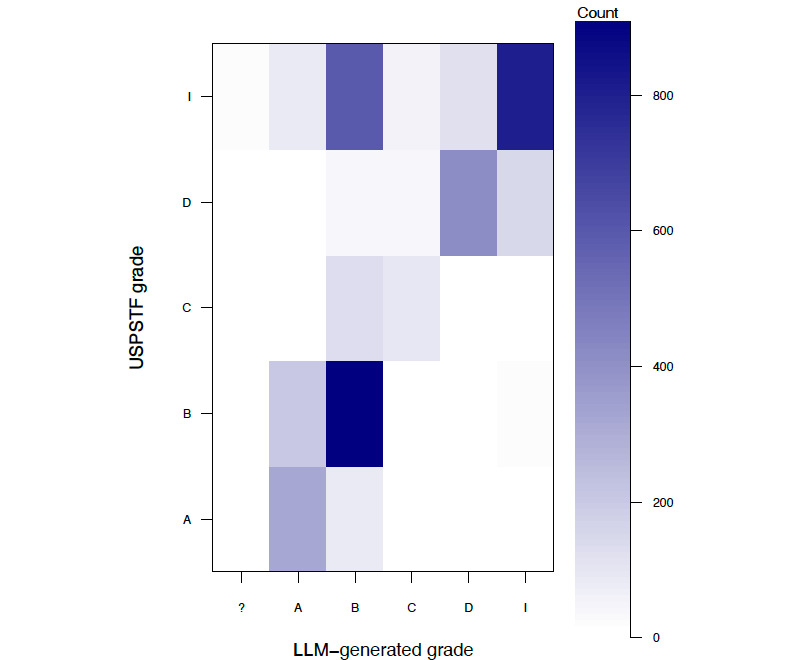
Joint distribution of large language model (LLM)–generated and US Preventive Services Task Force (USPSTF) grades (wave 1). The figure shows the cross-tabulation of LLM-generated grades and USPSTF grades for each preventive care activity in the study. Each cell represents an instance in which an LLM produced the grade shown on the horizontal axis (or did not generate a grade, indicated by “?” in the leftmost column) for a preventive care activity assigned the USPSTF grade shown on the vertical axis. Darker shading indicates a higher cell count.

**Figure 4:**
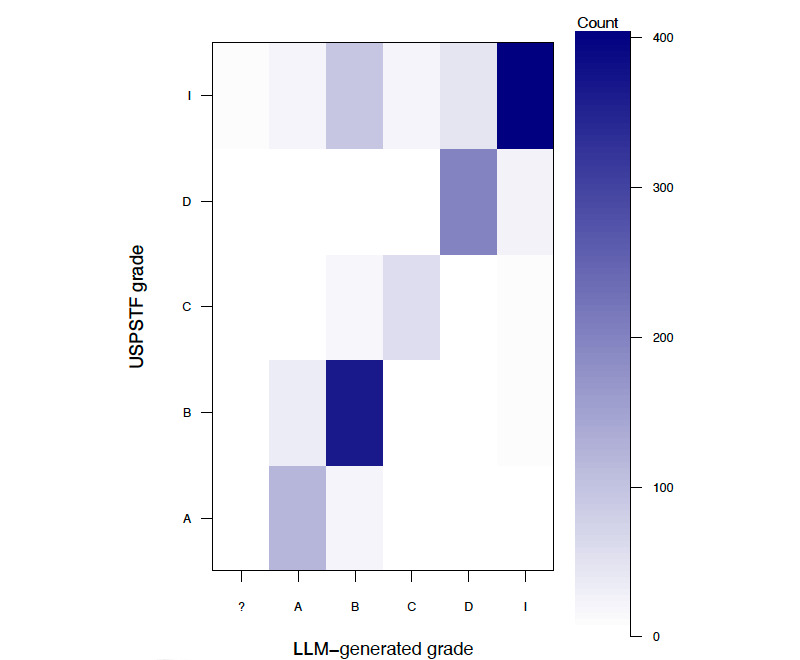
Joint distribution of large language model (LLM)–generated and US Preventive Services Task Force (USPSTF) grades (wave 2). The figure shows the cross-tabulation of LLM-generated grades and USPSTF grades for each preventive care activity in the study. Each cell represents an instance in which an LLM produced the grade shown on the horizontal axis (or no grade, indicated by “?” in the leftmost column) for a preventive care activity assigned the USPSTF grade shown on the vertical axis. Darker shading indicates a higher cell count.

Figures 3 and 4 visualize the cross-tabulation of USPSTF grades against LLM grades (instances in which the LLM did not issue a grade fall in the category “?”) when pooling data across models. The figures reveal that models primarily struggled to match USPSTF grades of “I,” as indicated by the nonzero counts in the first row of cells in each plot. Section 16 in Multimedia Appendix 1 shows that studying the cross-tabulation of LLM-generated and actual grades by model shows the same pattern: LLMs make fewer accurate inferences when grading activities assigned an “I” compared with activities assigned other letter grades.

## Discussion

The study’s findings suggest that LLMs vary in the rate at which they generate outputs consistent with USPSTF recommendations. Older models in wave 1 of our study issued responses to preventive care queries that matched USPSTF recommendations in less than 70% of all tests. Mismatches primarily resulted from the LLMs’ resistance to making a definitive statement (ie, equivocation or encouraging more information acquisition) even in cases where the USPSTF provided a clear recommendation. Newer models in wave 2 of our study reached higher levels of concordance with USPSTF recommendations, and this consistency improved further with particular prompting techniques—most notably, chain-of-thought prompting. Varying the prompting approaches in our study also revealed ways in which users could reduce the high rates of equivocation observed in both wave 1 and the baseline condition of wave 2. No prompting approach could drive the rate of equivocation or encouragement for users to seek more information below 50%, but this equivocation or encouragement for further information search might suggest an understandable bias toward safety, the empowerment of user decision-making, and/or the avoidance of direct medical advice provisioning (we thank a reviewer of our manuscript for this important point). When our study sought more detail from LLMs by prompting them to grade preventive care activities using the USPSTF grading scale, it found that some LLMs matched USPSTF grades in a substantial proportion of instances, while others only generated grades consistent with those of the USPSTF in a fraction of instances, and that fraction varied widely across models. Undoubtedly, users prompting an LLM to grade preventive care activities using the USPSTF scale could consult the official USPSTF grades and avoid the performance variation of LLMs; however, our test aimed to see whether an attempt to avoid equivocation through a narrow task would yield results that more closely approximated USPSTF guidance. Together, these findings suggest that the more recent LLMs in wave 2 can serve as promising sources of preventive care guidance, although occasionally deviating from authoritative recommendations, whereas the older models in wave 1 would serve as riskier substitutes for traditional sources of information about preventive care.

In advance of discussing the implications of these findings, several limitations warrant attention. Each limitation not only shapes how one should evaluate the research contained in this paper, but also how one should assess the present work’s contributions to practical measures and future research.

First, our study tests LLMs against an authoritative standard, but it does not compare LLMs against a human benchmark, which might be appropriate given that clinicians often serve as intermediaries between care seekers and USPSTF guidance (ie, a person delivers the USPSTF recommendation). This omission warrants attention because human guidance outside the USPSTF might diverge from official recommendations—a possibility implied by previous research reporting that preventive care recommendations across countries agree at low rates [34]. If such divergence is beneficial, then measuring LLM response consistency with a human benchmark is particularly important, as the human benchmark would presumably capture instances in which deviations from authoritative guidance enhance preventive care. If such divergence from authoritative recommendations is not beneficial, then the present study’s methods can be viewed as a test of LLM responses against a gold standard. Whether the performance of LLMs in preventive care tasks ought to be measured as consistency with USPSTF recommendations, other authoritative sources of guidance, or human clinicians is a discussion that future research and commentary should undertake.

Second, instead of using actual queries from real individuals to prompt the LLMs in our study, we used simulated queries. Although this approach conforms to well-established methods in machine behavior research [35], it fails to fulfill recent calls for patient-produced data in tests of LLMs in health care settings [36]. To the extent that individuals seek information about preventive care on their own using LLMs, our study’s inability to use the wording of real user queries reduces the verisimilitude of our investigation. On the other hand, if individuals rarely seek preventive care information via LLMs on their own, then our simulated queries might lack external validity due to the implausibility of individuals seeking such information on their own. Identifying which of these scenarios holds true will help future research carefully consider how LLMs can be tested in ways that fully reflect their actual use (or lack of use) in the acquisition of preventive care information.

Third, we study LLMs in a capacity—information retrieval—for which they were not designed [37]. Such criticism seems particularly salient given the accuracy of LLMs in other health care contexts, such as diagnostic reasoning [38]. We agree that individuals should use LLMs cautiously for information retrieval absent additional fine-tuning (eg, fine-tuning of the type shown to improve LLMs’ surgical recommendations in clinical contexts [39]). However, we also recognize that a broad user base might not demonstrate such caution. Commercial survey evidence indicates that 42% of surveyed professionals anticipate using AI for information retrieval in lieu of search engines, whereas only 24% anticipate using search engines in lieu of AI [40]. As a result, we deem it important to explore this use case—even if the use case is itself inadvisable—to understand the type of outputs that might result from individuals using LLMs as a source of information about preventive care options.

Fourth, our investigation relies on the use of LLMs not only as test subjects whose outputs we compare with USPSTF recommendations, but also as raters that classify other LLMs’ outputs so that we can assess both rates of LLM-USPSTF concordance and the content of LLM responses (eg, whether responses offer a recommendation or an equivocation). Although we found high rates of interrater reliability among the models used to perform these classification and summary activities, LLMs might differ from human experts in their classifications, or they might err in their adjudications. At the same time, human experts also might err in their classifications or exhibit biases in their categorizations. Coupled with our lack of resources to recruit human experts to evaluate the thousands of LLM responses that our systematic study of all USPSTF topic-population combinations required us to collect, the possibility of human errors and biases lessened our concern that LLMs would not categorize responses as soundly as humans and led us to opt for LLM raters to perform the large volume of work that our systematic investigation required.

Those outputs, in our investigation, suggest the potential for the newer LLMs in wave 2 of our study to be used as sources of preventive care information, but those LLMs—like the older ones in wave 1—still show high rates of equivocation or encouraging the pursuit of additional information, even when USPSTF recommendations are clear. Due to the high rates of such responses, this manner of advice provision deserves further scrutiny and debate. For one, our study put equivocation and encouragement of further information search in the same category in its classification scheme of LLM responses. Ultimately, we feel justified in this decision in the sense that encouraging the user to gather more information implies that the user should not treat the LLM output as definitive. That is, equivocation could come in the form of the LLM presenting multiple conflicting perspectives without offering a definitive recommendation, or it could come in the form of the LLM presenting one perspective and outsourcing the collection of alternative views: in either of those scenarios, the LLM does not offer a definitive recommendation. But, albeit a form of equivocation, encouraging the pursuit of further information does differ from offering multiple conflicting perspectives in that it requires more of the user and intimates that the user should not consider the LLM as the sole source of information on the subject. Future research should investigate whether such responses actually stimulate further information search and how such responses shape individuals’ perceptions of the reliability of information derived from the LLM being used. Second, equivocation warrants further scrutiny in the literature because it suggests a tradeoff between safeguards that aim to inspire a judicious approach to preventive care and the clarity of preventive care recommendations. Ultimately, sources of authoritative guidance such as the USPSTF have developed as a means of creating a clear signal about care activities in a noisy environment that might be difficult for most people to navigate; if LLMs reach sufficient levels of consistency with authoritative recommendations, then safety orientations that compel them to present a multisided perspective on a topic might simply create confusion on a subject where clarity exists and should be communicated. On the other hand, such safeguarding might play an important role in cultivating deliberation skills in patients, empowering patients, and ensuring patient-provider collaboration in care decisions and execution. We hope that our research adds to broader calls to analyze and discuss the tradeoffs resulting from model safeguards.

Our results also point toward how practitioners might further evaluate LLMs before contemplating clinical deployment and how they might need to consider new practices for communicating preventive care guidance in an information environment where patients can access LLMs. First, because interpretation of our findings should recognize that we solely provide a simulation-based benchmark to study LLM-USPSTF concordance under a limited set of prompting conditions, the results of this paper ought not influence care decisions; instead, for practitioners who remain interested in the prospects of using LLMs to deliver preventive care guidance, our findings could identify a smaller set of high-performing models for evaluation before clinical deployment, thus improving the efficiency of practitioners’ efforts to appraise whether LLMs can viably play a role in preventive care guidance in actual clinical settings. Second, the paper provides users with guidance on techniques that can improve LLMs’ outputs related to preventive care. For instance, the evidence in this paper suggests that a user who adopts chain-of-thought prompting and elects to use gpt-5.5-2026-04-23 will gain information consistent with the USPSTF in approximately 95% of all instances (Table 5). Using the information in this paper to guide the selection of prompting approaches will support individuals planning to further assess the viability of using LLMs to gain information about preventive care. Again, such plans should remain cognizant of the limited scope of our investigation and the need for clinical validation before adoption for uses that influences preventive care. Third, the findings of this paper inform evaluators about health categories of preventive care for which the use of LLMs is more apt to be successful—for instance, when seeking preventive care guidance concerning cancer, infectious diseases, and perinatal care—vs categories in which LLM guidance might be less helpful—for instance, with respect to guidance pertaining to development and behavior or to hard-to-classify care activities (Table 4). Directing further evaluation to those promising areas offers another means to enhance the efficiency of future assessments directly aimed at discerning the viability of LLM deployment in clinical settings, which remains beyond the scope of our investigation. Fourth, the findings hint at areas in which model developers and clinicians might collaborate to improve LLMs’ consistency with authoritative sources of care advice. Models showed less success matching USPSTF recommendations in areas that could involve multiple preventive care modalities (ie, some combination of counseling, preventive medication, and screening; Table 3), implying a need for expert guidance on how models might be fine-tuned to address those more complex care scenarios. Finally, evidence from our study showed that LLMs regularly encouraged further discussion of preventive care topics; thus, clinicians should consider augmenting existing communication practices concerning preventive care [41] with techniques that anticipate the questions that LLMs might inspire among care seekers.

The study’s results also suggest directions for future research. The automated methods used in the study have the potential to be deployed in other avenues of research concerning access to preventive care—for instance, in studies comparing Medicare coverage and USPSTF recommendations [42]. Additionally, if models continue to progress in their consistency with authoritative recommendations and if further research validates that they can be used safely in clinical settings, researchers should consider studying precisely how to embed models in clinical environments to offset the challenges associated with clinicians communicating an increasing number of preventive care guidelines to patients [9]. LLMs offer a promising means of addressing this problem, yet they could be implemented in various ways, such as by having clinicians communicate model outputs, guide patients’ use of LLMs, or encourage unguided, free-form use, among innumerable other methods of embedding models in clinical environments. Studying which of these methods would work best is an important area for future research if subsequent investigations validate the viability of using LLMs in clinical settings to inform individuals about preventive care. Those efforts also could incorporate investigations into how individuals interpret LLM-generated outputs. For instance, should LLM equivocation prove difficult to quell in response to users’ preventive care inquiries, then research studying how individuals respond to equivocation in preventive care recommendations will offer an empirical foundation for predicting and guiding patient behavior.

One final future direction suggested by our study carries import for research on AI evaluation generally, not solely in the realm of studying AI in the context of preventive care. Our study found that LLM raters tasked with broadly assessing a “match” between 2 natural-language passages often will find overall similarity in texts that those same raters would view as subtly different when provided a classification scheme that allows them to capture nuances of the text. This phenomenon resembles scale-format effects in survey research [43], where the granularity of scale units or the number of response categories can affect responses, and its relevance for evaluating LLM responses deserves attention. Our inclination, initially, was to view evidence of equivocation in instances of clear USPSTF recommendations as evidence that LLMs fail to provide advice consistent with USPSTF recommendations. A reviewer’s insightful observation pointed out that avoiding definitive statements and encouraging additional information search have positive benefits in the context of preventive care by, as noted above, empowering patients and encouraging their education while also avoiding the inappropriate replacement of clinical guidance. These important ideas from the reviewer should remind all researchers engaged in AI evaluation that they, too, must think critically about the values and concepts embedded in the criteria used to evaluate AI systems; in our investigation, taking such an approach means recognizing that the broad sentiments of an LLM output can match those of the USPSTF, even if the LLM output avoids making a definitive statement and encourages more information search, as would be appropriate for a nonauthoritative party contributing to preventive care education. Future work that guides researchers on how to recognize such nuances ex ante will enhance AI evaluation research.

These future avenues of research will extend this paper’s more modest objective. Here, using simulated user queries, our study found that LLMs vary in their consistency with USPSTF recommendations, with older models exhibiting less consistency and newer models echoing USPSTF guidance more regularly. Across both older and newer versions, LLMs resist making definitive statements and recommend the pursuit of further information; however, particular prompting approaches can be used to improve LLM-USPSTF concordance and reduce equivocation. Together, these findings indicate that LLMs have the potential to become an important source of individualized and scalable preventive care guidance, albeit with the caution that users should seek out better-performing models and use prompting techniques that yield higher rates of LLM-USPSTF concordance.

## Appendix

Multimedia Appendix 1 Supplementary materials.

## Abbreviations

LLM: large language model
USPSTF: US Preventive Services Task Force

## Data Availability

The datasets generated or analyzed during this study are available in the Open Science Framework repository [32].
