# Supplementary material for: Measuring Consistency Between Large Language Models’ Responses to Preventive Care Queries and Official US Preventive Services Task Force (USPSTF) Recommendations: Systematic Test Involving All USPSTF Preventive Care Topics via Simulated User Prompts
Source: JMIR AI. 2026 Jul 30;5:e87034. doi: 10.2196/87034 (PMC13473853; doi:10.2196/87034)
Supplement: Supplementary file 1 [file ai_v5i1e87034_app1.docx]

Supplementary Materials

**Measuring Consistency between Large Language Models’ Responses to Preventive-Care Queries and Official Recommendations of the U.S. Preventive Services Task Force: A Systematic Test of All USPSTF Preventive-Care Topics via Simulated-User Prompts**

Tim Johnson^1^ and Wolfgang Gaissmaier^2^

1. Atkinson Graduate School of Management, Willamette University, Salem, Oregon, USA 97301

2. Department of Psychology, Universität Konstanz, Konstanz, Germany 78457

Contents

[Supplementary Materials, Section 1.	1](#_Toc232879218)

[Supplementary Materials, Section 2.	12](#_Toc232879219)

[Supplementary Materials, Section 3.	14](#_Toc232879220)

[Supplementary Materials, Section 4.	25](#_Toc232879221)

[Supplementary Materials, Section 5	33](#_Toc232879222)

[Supplementary Materials, Section 6	37](#_Toc232879223)

[Supplementary Materials, Section 7	61](#_Toc232879224)

[Supplementary Materials, Section 8	64](#_Toc232879225)

[Supplementary Materials, Section 9	65](#_Toc232879226)

[Supplementary Materials, Section 10	67](#_Toc232879227)

[Supplementary Materials, Section 11	70](#_Toc232879228)

[Supplementary Materials, Section 12	71](#_Toc232879229)

[Supplementary Materials, Section 13	72](#_Toc232879230)

[Supplementary Materials, Section 14	74](#_Toc232879231)

[Supplementary Materials, Section 15	79](#_Toc232879232)

[Supplementary Materials, Section 16	81](#_Toc232879233)

[Supplementary Materials References	83](#_Toc232879234)

# Supplementary Materials, Section 1.

To develop the prompts used in the first portion of our study, which queried LLMs about the preventive health topics addressed in the USPSTF’s recommendations (withholding information about the USPSTF’s Recommendation and Grade), we developed a set of simulated queries and user characteristics. To do so, we prompted gpt-4o-2024-08-06, one of OpenAI’s most-advanced LLMs at the time of the study, with a prompt that instructed it to create a both question and a simulated persona relevant to the USPSTF Topic and Population designated for a particular USPSTF Recommendation. The prompt read as follows (with information in chevron brackets (“<” and “>”) varied in each iteration):

**User Prompt**

I really appreciate how helpful and insightful you are. Would you mind helping me with another task? I have data from the U.S. Preventive Services Task Force. The data contain information about medical interventions -- for instance, *Abdominal Aortic Aneurysm Screening* -- and the population that might receive the intervention -- for instance, *Men aged 65 to 75 years who have ever smoked*. I need to create questions in which someone from the population that might receive the intervention asks whether or not they should receive the intervention. For instance, if the *topic* is *Abdominal Aortic Aneurysm Screening* and the population is *Men aged 65 to 75 years who have ever smoked*, then I would need a question such as this written:

I am a 70-year old man who has smoked. Should I receive screening for abdominal aortic aneurysm?

I would be very grateful if you could please provide me such a question for the topic of *<USPSTF TOPIC HERE>* with regards to the population *<USPSTF POPULATION HERE>*. Thank you again for your help; please provide such a question with no other text, just the question alone.

**System Prompt**

You are a helpful assistant who can create helpful questions asking about health interventions.

We submitted this prompt to gpt-4o-2024-08-06 a total of 142 times—that is, once for each of the Topic-Population combinations listed on the USPSTF website. Those queries resulted in a set of LLM-generated questions that we could use as prompts in our test of whether widely available LLMs provide guidance consistent with the USPSTF. We present those LLM-generated questions (i.e. the prompts for the test) in the rightmost column of the below table (Table SM0), with the relevant topic and population appearing in the left and center rows, respectively:

**Table. LLM-Generated Questions for Each Topic and Population**

| **Topic** | **Population** | **LLM-Generated Question** |
| --- | --- | --- |
| Syphilis Infection During Pregnancy: Screening | Asymptomatic pregnant women | I am an asymptomatic pregnant woman. Should I be screened for syphilis infection during pregnancy? |
| Breastfeeding: Primary Care Behavioral Counseling Interventions | Pregnant and postpartum women | I am a pregnant woman and want to know if I should receive primary care behavioral counseling interventions to support breastfeeding. |
| Food Insecurity: Screening | Children, adolescents, and adults | I am a parent of a young child. Should my child be screened for food insecurity? |
| Osteoporosis to Prevent Fractures: Screening | Women 65 years or older | I am a 68-year-old woman. Should I be screened for osteoporosis to prevent fractures? |
| Osteoporosis to Prevent Fractures: Screening | Postmenopausal women younger than 65 years with 1 or more risk factors for osteoporosis | I am a 60-year-old postmenopausal woman with risk factors for osteoporosis. Should I be screened for osteoporosis to prevent fractures? |
| Osteoporosis to Prevent Fractures: Screening | Men | I am a man concerned about osteoporosis. Should I be screened to prevent fractures? |
| Iron Deficiency and Iron Deficiency Anemia During Pregnancy: Screening and Supplementation | Asymptomatic pregnant adolescents and adults | I am a pregnant adult with no symptoms of iron deficiency. Should I be screened for iron deficiency and iron deficiency anemia, or take iron supplements during my pregnancy? |
| Iron Deficiency and Iron Deficiency Anemia During Pregnancy: Screening and Supplementation | Asymptomatic pregnant adolescents and adults | I am a pregnant adult with no symptoms of iron deficiency. Should I be screened for iron deficiency and iron deficiency anemia or take iron supplements? |
| High Body Mass Index in Children and Adolescents: Interventions | Children and adolescents 6 years or older | I have a 10-year-old child. Should they receive interventions for high body mass index? |
| Falls Prevention in Community-Dwelling Older Adults: Interventions | Community-dwelling adults 65 years or older | I am a 68-year-old adult living in the community. Should I consider interventions for falls prevention? |
| Falls Prevention in Community-Dwelling Older Adults: Interventions | Community-dwelling adults 65 years or older | I am a 68-year-old adult living in the community. Should I consider interventions for falls prevention? |
| Breast Cancer: Screening | Women aged 40 to 74 years | I am a 50-year-old woman. Should I receive screening for breast cancer? |
| Breast Cancer: Screening | Women 75 years or older | I am a 78-year-old woman. Should I receive screening for breast cancer? |
| Breast Cancer: Screening | Women with dense breasts | I am a woman with dense breasts. Should I receive screening for breast cancer? |
| Prevention of Child Maltreatment: Primary Care Interventions | Children and adolescents younger than 18 years without signs and symptoms of or known exposure to maltreatment | I have a child who is 10 years old and has no signs or symptoms of maltreatment. Should they receive any primary care interventions to prevent child maltreatment? |
| Speech and Language Delay and Disorders in Children: Screening | Children 5 years or younger without signs or symptoms of speech and language delay and disorders | My child is 4 years old and does not show any signs of speech or language delay. Should they be screened for speech and language delay and disorders? |
| Oral Health in Adults: Screening and Preventive Interventions | Asymptomatic adults 18 years or older | I am a 30-year-old adult with no symptoms. Should I receive screening and preventive interventions for oral health? |
| Oral Health in Adults: Screening and Preventive Interventions | Asymptomatic adults 18 years or older | I am a 30-year-old adult with no symptoms. Should I receive screening and preventive interventions for oral health? |
| Oral Health in Children and Adolescents Aged 5 to 17 Years: Screening and Preventive Interventions | Asymptomatic children and adolescents aged 5 to 17 years | I have a 10-year-old child who is asymptomatic. Should they receive screening and preventive interventions for oral health? |
| Oral Health in Children and Adolescents Aged 5 to 17 Years: Screening and Preventive Interventions | Asymptomatic children and adolescents aged 5 to 17 years | I have a 10-year-old child who is asymptomatic. Should they receive screening and preventive interventions for oral health? |
| Hypertensive Disorders of Pregnancy: Screening | Asymptomatic pregnant persons | I am currently pregnant and have no symptoms of hypertension. Should I be screened for hypertensive disorders of pregnancy? |
| Prevention of Acquisition of HIV: Preexposure Prophylaxis | Adolescents and adults at increased risk of HIV | I am an adult who is at increased risk of HIV. Should I consider taking preexposure prophylaxis to prevent acquiring HIV? |
| Folic Acid Supplementation to Prevent Neural Tube Defects: Preventive Medication | Persons who plan to or could become pregnant | I am planning to become pregnant. Should I take folic acid supplements to prevent neural tube defects? |
| Lipid Disorders in Children and Adolescents: Screening | Asymptomatic children and adolescents 20 years or younger | I am a 15-year-old who has no symptoms. Should I be screened for lipid disorders? |
| Anxiety Disorders in Adults: Screening | Adults 64 years or younger, including pregnant and postpartum persons | I am a 30-year-old adult, and I am currently pregnant. Should I be screened for anxiety disorders? |
| Anxiety Disorders in Adults: Screening | Older adults 65 years or older | I am a 68-year-old adult. Should I be screened for anxiety disorders? |
| Depression and Suicide Risk in Adults: Screening | Adults, including pregnant and postpartum persons, and older adults (65 years or older) | I am a 68-year-old adult, and I am currently pregnant. Should I be screened for depression and suicide risk? |
| Depression and Suicide Risk in Adults: Screening | Adults, including pregnant and postpartum persons, and older adults (65 years or older) | I am a 68-year-old adult, and I am currently pregnant. Should I be screened for depression and suicide risk? |
| Latent Tuberculosis Infection in Adults: Screening | Asymptomatic adults at increased risk of latent tuberculosis infection (LTBI) | I am an asymptomatic adult who is at increased risk of latent tuberculosis infection. Should I be screened for latent tuberculosis infection? |
| Skin Cancer: Screening | Asymptomatic adolescents and adults | I am an asymptomatic adult. Should I receive screening for skin cancer? |
| Genital Herpes Infection: Serologic Screening | Asymptomatic adolescents and adults, including pregnant persons | I am an asymptomatic adult. Should I receive serologic screening for genital herpes infection? |
| Obstructive Sleep Apnea in Adults: Screening | General adult population | I am an adult. Should I be screened for obstructive sleep apnea? |
| Hormone Therapy in Postmenopausal Persons: Primary Prevention of Chronic Conditions | Postmenopausal persons | I am a postmenopausal person. Should I consider hormone therapy for the primary prevention of chronic conditions? |
| Hormone Therapy in Postmenopausal Persons: Primary Prevention of Chronic Conditions | Postmenopausal persons who have had a hysterectomy | I am a postmenopausal person who has had a hysterectomy. Should I consider hormone therapy for the primary prevention of chronic conditions? |
| Anxiety in Children and Adolescents: Screening | Children and adolescents aged 8 to 18 years | My child is 12 years old. Should they be screened for anxiety? |
| Anxiety in Children and Adolescents: Screening | Children 7 years or younger | My child is 6 years old. Should they be screened for anxiety? |
| Depression and Suicide Risk in Children and Adolescents: Screening | Adolescents aged 12 to 18 years | I am a 15-year-old adolescent. Should I be screened for depression and suicide risk? |
| Depression and Suicide Risk in Children and Adolescents: Screening | Children 11 years or younger | My child is 10 years old. Should they be screened for depression and suicide risk? |
| Depression and Suicide Risk in Children and Adolescents: Screening | Children and adolescents | My child is 14 years old. Should they be screened for depression and suicide risk? |
| Syphilis Infection in Nonpregnant Adolescents and Adults: Screening | Asymptomatic, nonpregnant adolescents and adults who are at increased risk for syphilis infection | I am an asymptomatic, nonpregnant adult who is at increased risk for syphilis infection. Should I be screened for syphilis? |
| Prediabetes and Type 2 Diabetes in Children and Adolescents: Screening | Asymptomatic children and adolescents younger than 18 years | I am a 15-year-old who doesn't have any symptoms of diabetes. Should I be screened for prediabetes and type 2 diabetes? |
| Statin Use for the Primary Prevention of Cardiovascular Disease in Adults: Preventive Medication | Adults aged 40 to 75 years who have 1 or more cardiovascular risk factors and an estimated 10-year cardiovascular disease (CVD) risk of 10% or greater | I am a 50-year-old adult with high blood pressure and an estimated 10-year cardiovascular disease risk of 12%. Should I start using statins for the primary prevention of cardiovascular disease? |
| Statin Use for the Primary Prevention of Cardiovascular Disease in Adults: Preventive Medication | Adults aged 40 to 75 years who have 1 or more cardiovascular risk factors and an estimated 10-year CVD risk of 7.5% to less than 10% | I am a 50-year-old adult with high blood pressure and an estimated 10-year cardiovascular disease risk of 8%. Should I start taking statins for the primary prevention of cardiovascular disease? |
| Statin Use for the Primary Prevention of Cardiovascular Disease in Adults: Preventive Medication | Adults 76 years or older | I am 78 years old. Should I start taking statins to prevent cardiovascular disease? |
| Healthy Diet and Physical Activity for Cardiovascular Disease Prevention in Adults Without Cardiovascular Disease Risk Factors: Behavioral Counseling Interventions | Adults 18 years or older without known cardiovascular disease risk factors | I am a 30-year-old adult without any known cardiovascular disease risk factors. Should I receive behavioral counseling interventions for a healthy diet and physical activity to prevent cardiovascular disease? |
| Vitamin, Mineral, and Multivitamin Supplementation to Prevent Cardiovascular Disease and Cancer: Preventive Medication | Community-dwelling, nonpregnant adults | I am a 50-year-old community-dwelling, nonpregnant adult. Should I take vitamin, mineral, or multivitamin supplements to prevent cardiovascular disease and cancer? |
| Vitamin, Mineral, and Multivitamin Supplementation to Prevent Cardiovascular Disease and Cancer: Preventive Medication | Community-dwelling, nonpregnant adults | I am a community-dwelling, nonpregnant adult. Should I take vitamin, mineral, or multivitamin supplements to prevent cardiovascular disease and cancer? |
| Vitamin, Mineral, and Multivitamin Supplementation to Prevent Cardiovascular Disease and Cancer: Preventive Medication | Community-dwelling, nonpregnant adults | I am a community-dwelling, nonpregnant adult. Should I take vitamin, mineral, or multivitamin supplements to prevent cardiovascular disease and cancer? |
| Impaired Visual Acuity in Older Adults: Screening | Asymptomatic adults 65 years or older | I am a 68-year-old adult with no symptoms of vision problems. Should I be screened for impaired visual acuity? |
| Primary Open-Angle Glaucoma: Screening | Asymptomatic adults 40 years or older | I am a 45-year-old adult with no symptoms. Should I be screened for primary open-angle glaucoma? |
| Chronic Obstructive Pulmonary Disease: Screening | Asymptomatic adults | I am an asymptomatic adult. Should I be screened for chronic obstructive pulmonary disease? |
| Aspirin Use to Prevent Cardiovascular Disease: Preventive Medication | Adults aged 40 to 59 years with a 10% or greater 10-year cardiovascular disease (CVD) risk | I am a 45-year-old adult with a 10% or greater 10-year cardiovascular disease risk. Should I use aspirin to prevent cardiovascular disease? |
| Aspirin Use to Prevent Cardiovascular Disease: Preventive Medication | Adults 60 years or older | I am a 65-year-old adult. Should I use aspirin to prevent cardiovascular disease? |
| Eating Disorders in Adolescents and Adults: Screening | Asymptomatic adolescents and adults | I am an asymptomatic adult. Should I be screened for eating disorders? |
| Atrial Fibrillation: Screening | Asymptomatic adults 50 years or older | I am a 55-year-old adult with no symptoms. Should I be screened for atrial fibrillation? |
| Prevention of Dental Caries in Children Younger Than 5 Years: Screening and Interventions | Children younger than 5 years | My child is 4 years old. Should they receive screening and interventions for the prevention of dental caries? |
| Prevention of Dental Caries in Children Younger Than 5 Years: Screening and Interventions | Children younger than 5 years | My child is 4 years old. Should they receive screening and interventions for the prevention of dental caries? |
| Prevention of Dental Caries in Children Younger Than 5 Years: Screening and Interventions | Children younger than 5 years | My child is 4 years old. Should they receive screening and interventions for the prevention of dental caries? |
| Aspirin Use to Prevent Preeclampsia and Related Morbidity and Mortality: Preventive Medication | Pregnant persons at high risk for preeclampsia | I am pregnant and at high risk for preeclampsia. Should I take aspirin to prevent preeclampsia and related complications? |
| Chlamydia and Gonorrhea: Screening | Sexually active women, including pregnant persons | I am a sexually active woman. Should I be screened for chlamydia and gonorrhea? |
| Chlamydia and Gonorrhea: Screening | Sexually active women, including pregnant persons | I am a sexually active woman. Should I be screened for chlamydia and gonorrhea? |
| Chlamydia and Gonorrhea: Screening | Sexually active men | I am a sexually active man. Should I be screened for chlamydia and gonorrhea? |
| Prediabetes and Type 2 Diabetes: Screening | Asymptomatic adults aged 35 to 70 years who have overweight or obesity | I am a 50-year-old adult with overweight. Should I be screened for prediabetes and type 2 diabetes? |
| Gestational Diabetes: Screening | Asymptomatic pregnant persons at 24 weeks of gestation or after | I am 28 weeks pregnant and have no symptoms of diabetes. Should I be screened for gestational diabetes? |
| Gestational Diabetes: Screening | Asymptomatic pregnant persons before 24 weeks of gestation | I am pregnant and currently less than 24 weeks along with no symptoms of diabetes. Should I be screened for gestational diabetes? |
| Healthy Weight and Weight Gain In Pregnancy: Behavioral Counseling Interventions | Pregnant persons | I am currently pregnant. Should I receive behavioral counseling interventions to maintain a healthy weight and manage weight gain during my pregnancy? |
| Colorectal Cancer: Screening | Adults aged 50 to 75 years | I am a 60-year-old adult. Should I receive screening for colorectal cancer? |
| Colorectal Cancer: Screening | Adults aged 45 to 49 years | I am a 47-year-old adult. Should I receive screening for colorectal cancer? |
| Colorectal Cancer: Screening | Adults aged 76 to 85 years | I am an 80-year-old adult. Should I receive screening for colorectal cancer? |
| Hypertension in Adults: Screening | Adults 18 years or older without known hypertension | I am a 45-year-old adult without known hypertension. Should I be screened for hypertension? |
| Vitamin D Deficiency in Adults: Screening | Asymptomatic, community-dwelling, nonpregnant adults | I am a 45-year-old asymptomatic, community-dwelling, nonpregnant adult. Should I be screened for vitamin D deficiency? |
| Hearing Loss in Older Adults: Screening | Asymptomatic adults 50 years or older | I am a 65-year-old adult with no symptoms of hearing loss. Should I be screened for hearing loss? |
| Lung Cancer: Screening | Adults aged 50 to 80 years who have a 20 pack-year smoking history and currently smoke or have quit within the past 15 years | I am a 65-year-old adult with a 20 pack-year smoking history, and I currently smoke. Should I receive screening for lung cancer? |
| Asymptomatic Carotid Artery Stenosis: Screening | Asymptomatic adults | I am an asymptomatic adult. Should I be screened for carotid artery stenosis? |
| Tobacco Smoking Cessation in Adults, Including Pregnant Persons: Interventions | Nonpregnant adults | I am a nonpregnant adult who smokes. Should I consider interventions for tobacco smoking cessation? |
| Tobacco Smoking Cessation in Adults, Including Pregnant Persons: Interventions | Pregnant persons | I am pregnant and currently smoke. Should I receive interventions to help me quit smoking? |
| Tobacco Smoking Cessation in Adults, Including Pregnant Persons: Interventions | Pregnant persons | I am pregnant and currently smoke. Should I receive interventions to help me quit smoking? |
| Tobacco Smoking Cessation in Adults, Including Pregnant Persons: Interventions | All adults | I am an adult who smokes. Should I consider interventions for tobacco smoking cessation? |
| Hepatitis B Virus Infection in Adolescents and Adults: Screening | Adolescents and adults at increased risk for infection | I am an adult who is at increased risk for hepatitis B infection. Should I be screened for hepatitis B virus infection? |
| Healthy Diet and Physical Activity for Cardiovascular Disease Prevention in Adults With Cardiovascular Risk Factors: Behavioral Counseling Interventions | Adults with cardiovascular disease risk factors | I am an adult with cardiovascular disease risk factors. Should I receive behavioral counseling interventions for a healthy diet and physical activity to prevent cardiovascular disease? |
| High Blood Pressure in Children and Adolescents: Screening | Children and adolescents | I have a 12-year-old child. Should they be screened for high blood pressure? |
| Sexually Transmitted Infections: Behavioral Counseling | Sexually active adolescents and adults at increased risk | I am a sexually active adult who is at increased risk for sexually transmitted infections. Should I receive behavioral counseling for sexually transmitted infections? |
| Unhealthy Drug Use: Screening | Adults age 18 years or older | I am a 30-year-old adult. Should I be screened for unhealthy drug use? |
| Unhealthy Drug Use: Screening | Adolescents | I am an adolescent. Should I be screened for unhealthy drug use? |
| Illicit Drug Use in Children, Adolescents, and Young Adults: Primary Care-Based Interventions | Children, adolescents, and young adults | I am a young adult concerned about illicit drug use. Should I receive a primary care-based intervention to address this issue? |
| Tobacco Use in Children and Adolescents: Primary Care Interventions | School-aged children and adolescents who have not started to use tobacco | I am a parent of a 14-year-old who has not started using tobacco. Should we consider primary care interventions to prevent tobacco use? |
| Tobacco Use in Children and Adolescents: Primary Care Interventions | School-aged children and adolescents who use tobacco | I am a 15-year-old who uses tobacco. Should I receive any primary care interventions to help me quit? |
| Bacterial Vaginosis in Pregnant Persons to Prevent Preterm Delivery: Screening | Pregnant persons not at increased risk for preterm delivery | I am pregnant and not at increased risk for preterm delivery. Should I be screened for bacterial vaginosis to prevent preterm delivery? |
| Bacterial Vaginosis in Pregnant Persons to Prevent Preterm Delivery: Screening | Pregnant persons at increased risk for preterm delivery | I am pregnant and at increased risk for preterm delivery. Should I be screened for bacterial vaginosis to prevent preterm delivery? |
| Hepatitis C Virus Infection in Adolescents and Adults: Screening | Adults aged 18 to 79 years | I am a 45-year-old adult. Should I be screened for hepatitis C virus infection? |
| Cognitive Impairment in Older Adults: Screening | Older adults | I am an older adult concerned about my memory and cognitive health. Should I be screened for cognitive impairment? |
| Abdominal Aortic Aneurysm: Screening | Men aged 65 to 75 years who have ever smoked | I am a 68-year-old man who has smoked in the past. Should I get screened for an abdominal aortic aneurysm? |
| Abdominal Aortic Aneurysm: Screening | Men aged 65 to 75 years who have never smoked | I am a 72-year-old man who has never smoked. Should I receive screening for abdominal aortic aneurysm? |
| Abdominal Aortic Aneurysm: Screening | Women who have never smoked | I am a 68-year-old woman who has never smoked. Should I receive screening for abdominal aortic aneurysm? |
| Abdominal Aortic Aneurysm: Screening | Women aged 65 to 75 years who have ever smoked | I am a 70-year-old woman who has smoked. Should I receive screening for abdominal aortic aneurysm? |
| Asymptomatic Bacteriuria in Adults: Screening | Pregnant persons | I am pregnant. Should I be screened for asymptomatic bacteriuria? |
| Asymptomatic Bacteriuria in Adults: Screening | Nonpregnant adults | I am a nonpregnant adult. Should I be screened for asymptomatic bacteriuria? |
| Breast Cancer: Medication Use to Reduce Risk | Women at increased risk for breast cancer aged 35 years or older | I am a 40-year-old woman at increased risk for breast cancer. Should I use medication to reduce my risk of developing breast cancer? |
| Breast Cancer: Medication Use to Reduce Risk | Women not at increased risk for breast cancer aged 35 years or older | I am a 40-year-old woman who is not at increased risk for breast cancer. Should I use medication to reduce my risk of developing breast cancer? |
| BRCA-Related Cancer: Risk Assessment, Genetic Counseling, and Genetic Testing | Women with a personal or family history of breast, ovarian, tubal, or peritoneal cancer or an ancestry associated with BRCA1/2 gene mutation | I am a woman with a family history of breast cancer and an ancestry associated with BRCA1/2 gene mutation. Should I undergo risk assessment, genetic counseling, and genetic testing for BRCA-related cancer? |
| BRCA-Related Cancer: Risk Assessment, Genetic Counseling, and Genetic Testing | Women whose personal or family history or ancestry is not associated with potential harmful BRCA1/2 gene mutations | I am a woman with no personal or family history or ancestry associated with potential harmful BRCA1/2 gene mutations. Should I undergo risk assessment, genetic counseling, or genetic testing for BRCA-related cancer? |
| Pancreatic Cancer: Screening | Adults | I am an adult. Should I receive screening for pancreatic cancer? |
| Hepatitis B Virus Infection in Pregnant Women: Screening | Pregnant women | I am currently pregnant. Should I be screened for hepatitis B virus infection? |
| Human Immunodeficiency Virus (HIV) Infection: Screening | Pregnant persons | I am pregnant. Should I be screened for HIV infection? |
| Human Immunodeficiency Virus (HIV) Infection: Screening | Adolescents and adults aged 15 to 65 years | I am a 30-year-old adult. Should I get screened for HIV infection? |
| Perinatal Depression: Preventive Interventions | Pregnant and postpartum persons | I am currently pregnant and concerned about perinatal depression. Should I consider preventive interventions for perinatal depression? |
| Ocular Prophylaxis for Gonococcal Ophthalmia Neonatorum: Preventive Medication | Newborns | Should my newborn receive ocular prophylaxis to prevent gonococcal ophthalmia neonatorum? |
| Unhealthy Alcohol Use in Adolescents and Adults: Screening and Behavioral Counseling Interventions | Adults 18 years or older, including pregnant women | I am a 30-year-old adult. Should I be screened for unhealthy alcohol use and receive behavioral counseling if necessary? |
| Unhealthy Alcohol Use in Adolescents and Adults: Screening and Behavioral Counseling Interventions | Adolescents aged 12 to 17 years | I am a 15-year-old adolescent. Should I be screened for unhealthy alcohol use and receive behavioral counseling interventions? |
| Intimate Partner Violence, Elder Abuse, and Abuse of Vulnerable Adults: Screening | Women of reproductive age | I am a woman of reproductive age. Should I be screened for intimate partner violence, elder abuse, and abuse of vulnerable adults? |
| Intimate Partner Violence, Elder Abuse, and Abuse of Vulnerable Adults: Screening | Older or vulnerable adults | I am an older adult who may be considered vulnerable. Should I be screened for intimate partner violence, elder abuse, and abuse of vulnerable adults? |
| Weight Loss to Prevent Obesity-Related Morbidity and Mortality in Adults: Behavioral Interventions | Adults | I am an adult concerned about obesity-related health issues. Should I consider behavioral interventions for weight loss to prevent these problems? |
| Cervical Cancer: Screening | Women aged 21 to 65 years | I am a 30-year-old woman. Should I receive screening for cervical cancer? |
| Cervical Cancer: Screening | Women younger than 21 years | I am a 20-year-old woman. Should I receive screening for cervical cancer? |
| Cervical Cancer: Screening | Women who have had a hysterectomy | I am a woman who has had a hysterectomy. Should I receive screening for cervical cancer? |
| Cervical Cancer: Screening | Women older than 65 years | I am a 68-year-old woman. Should I continue to receive screening for cervical cancer? |
| Peripheral Artery Disease and Cardiovascular Disease: Screening and Risk Assessment With the Ankle-Brachial Index | Adults | I am an adult concerned about my risk for peripheral artery disease and cardiovascular disease. Should I be screened using the ankle-brachial index? |
| Cardiovascular Disease: Risk Assessment With Nontraditional Risk Factors | Adults | I am an adult concerned about my heart health. Should I consider a risk assessment for cardiovascular disease using nontraditional risk factors? |
| Cardiovascular Disease Risk: Screening With Electrocardiography | Adults at low risk of CVD events | I am an adult with a low risk of cardiovascular disease events. Should I undergo screening with electrocardiography for cardiovascular disease risk? |
| Cardiovascular Disease Risk: Screening With Electrocardiography | Adults at intermediate or high risk of CVD events | I am an adult at intermediate risk of cardiovascular disease events. Should I undergo screening with electrocardiography for cardiovascular disease risk? |
| Prostate Cancer: Screening | Men aged 55 to 69 years | I am a 60-year-old man. Should I receive screening for prostate cancer? |
| Prostate Cancer: Screening | Men 70 years and older | I am a 72-year-old man. Should I receive screening for prostate cancer? |
| Vitamin D, Calcium, or Combined Supplementation for the Primary Prevention of Fractures in Community-Dwelling Adults: Preventive Medication | Postmenopausal women | I am a postmenopausal woman. Should I take vitamin D, calcium, or combined supplements to prevent fractures? |
| Vitamin D, Calcium, or Combined Supplementation for the Primary Prevention of Fractures in Community-Dwelling Adults: Preventive Medication | Men and premenopausal women | I am a 45-year-old man. Should I take vitamin D, calcium, or combined supplements to prevent fractures? |
| Vitamin D, Calcium, or Combined Supplementation for the Primary Prevention of Fractures in Community-Dwelling Adults: Preventive Medication | Postmenopausal women | I am a postmenopausal woman. Should I take vitamin D, calcium, or combined supplements to prevent fractures? |
| Skin Cancer Prevention: Behavioral Counseling | Young adults, adolescents, children, and parents of young children | I am a parent of a young child. Should I seek behavioral counseling for skin cancer prevention for my child? |
| Skin Cancer Prevention: Behavioral Counseling | Adults older than 24 years with fair skin types | I am a 30-year-old adult with fair skin. Should I receive behavioral counseling for skin cancer prevention? |
| Skin Cancer Prevention: Behavioral Counseling | Adults | I am an adult concerned about skin cancer. Should I receive behavioral counseling for skin cancer prevention? |
| Ovarian Cancer: Screening | Asymptomatic women | I am an asymptomatic woman. Should I receive screening for ovarian cancer? |
| Adolescent Idiopathic Scoliosis: Screening | Children and adolescents aged 10 to 18 years | I am a 15-year-old adolescent. Should I be screened for adolescent idiopathic scoliosis? |
| Vision in Children Ages 6 Months to 5 Years: Screening | Children aged 3 to 5 years | My child is 4 years old. Should they receive vision screening? |
| Vision in Children Ages 6 Months to 5 Years: Screening | Children younger than 3 years | My child is 2 years old. Should they be screened for vision problems? |
| Thyroid Cancer: Screening | Adults | I am an adult. Should I receive screening for thyroid cancer? |
| Celiac Disease: Screening | Asymptomatic adults, adolescents, and children | I am an asymptomatic adult. Should I be screened for celiac disease? |
| Autism Spectrum Disorder in Young Children: Screening | Children aged 18 to 30 months | My child is 24 months old. Should they be screened for autism spectrum disorder? |
| Iron Deficiency Anemia in Young Children: Screening | Children ages 6 to 24 months | My child is 18 months old. Should they be screened for iron deficiency anemia? |
| Thyroid Dysfunction: Screening | Nonpregnant, asymptomatic adults | I am a nonpregnant adult with no symptoms. Should I be screened for thyroid dysfunction? |
| Oral Cancer: Screening | Asymptomatic Adults | I am an asymptomatic adult. Should I be screened for oral cancer? |
| Bladder Cancer in Adults: Screening | Asymptomatic Adults | I am an asymptomatic adult. Should I be screened for bladder cancer? |
| Testicular Cancer: Screening | Adolescent and Adult Men | I am a 25-year-old man. Should I be screened for testicular cancer? |
| Rh(D) Incompatibility: Screening | Pregnant women, during the first pregnancy-related care visit | I am a pregnant woman attending my first pregnancy-related care visit. Should I be screened for Rh(D) incompatibility? |
| Rh(D) Incompatibility: Screening | Unsensitized Rh(D)-negative pregnant women | I am an Rh(D)-negative pregnant woman who has not been sensitized. Should I be screened for Rh(D) incompatibility? |

# Supplementary Materials, Section 2.

To avoid the risk of bias in our assessment of whether LLM responses matched the recommendations of the USPSTF, we tasked the OpenAI model gpt-4.1-mini-2025-04-14 with assessing whether the LLM-generated responses coincided with the recommendations of the USPSTF. To do so, we automated the assessment by inserting a LLM-generated response and a USPSTF recommendation into the following prompt, which instructed gpt-4.1-mini-2025-04-14 to compare the LLM-generated response with the official USPSTF recommendation:

**User Prompt**

Thank you for all of your help recently. I greatly appreciate your top-quality work. I was hoping to get your help again. I recently collected a set of recommendations about preventative healthcare in particular scenarios (I call these *scenario recommendations*) and I would like to compare those recommendations to the *official recommendations* of the U.S. Preventive Services Task Force. Whenever the *scenario recommendation* matches the *official recommendation*, I would write a 1. Whenever the *scenario recommendation* DOES NOT match the *official recommendation*, I would write a 0. For instance, consider the following *scenario recommendation* and *official recommendation* (I will place each recommendation in chevron brackets (< >):

*scenario recommendation*: <Given that you are a 68-year old male and have smoked, you should get screened for abdominal aortic aneurysm (AAA) with ultrasonography.>

*official recommendation*: <The USPSTF recommends 1-time screening for abdominal aortic aneurysm (AAA) with ultrasonography in men aged 65 to 75 years who have ever smoked.>

In this instance, I would return a value of 1 (and only the value 1 -- no other information or explanation). Now, consider a different *scenario recommendation* and *official recommendation* (I will place each recommendation in chevron brackets (< >):

*scenario recommendation*: <Health screening is important, but you should not get screened even once for abdominal aortic aneurysm (AAA) with ultrasonography, even though you are a man aged 65-75 years old.>

*official recommendation*: <The USPSTF recommends 1-time screening for abdominal aortic aneurysm (AAA) with ultrasonography in men aged 65 to 75 years who have ever smoked.>

In this instance, I would write a 0 (and only a 0 -- no other information or explanation) as the *scenario recommendation* does not make the same recommendation as the *official recommendation*. Notice that I do not care why the recommendation differs. I only care about whether the recommendations convey the same meaning. The meaning does not need to be exactly the same, it is just that the recommendations should offer the same guidance. With those examples provided, please respond with only a 1 or a 0 to indicate whether the following *scenario recommendation* conveys the same meaning as the following *official recommendation*:

*scenario recommendation*: <LLM RECOMMENDATION HERE>

*official recommendation*: <USPSTF RECOMMENDATION HERE>

**System Prompt**

You are a careful reader and qualitative researcher who can make judicious decisions about whether two pieces of text give the same recommendation.

# Supplementary Materials, Section 3.

This section of the supplementary materials presents robustness checks of our main analysis in which we study the responses of each LLM rater in our automated assessment of LLM-USPSTF agreement. As mentioned in the main text, to reduce bias in the assessment of whether LLM recommendations coincided with the recommendations of the USPSTF, we performed the automated classification of LLM-USPSTF agreement described in the previous section of these supplementary materials two times (i.e. with two raters) so as to gauge the reliability of the LLM’s assessments. In the main text, we presented analyses from cases in which both raters agree, excluding items in which the raters did not agree. In this section of the supplementary materials, we present the results of each rater separately. These results, which appear consecutively on the next two pages of this supplement, provide two replications of our study for comparison with the findings from the main text, which concern cases in which both of the LLM raters agree in their assessments.

Results Obtained When Solely Using the Classifications of Rater 1

When solely examining Rater 1’s classifications, we found that LLM responses matched USPSTF recommendations in 54.65% of trials (2173 out of 3976 trials) as compared to 55.11% of trials (2070 out of 3756) in which both raters agreed (i.e. the findings presented the main text). The median rate of concordance equaled 57.04% when using rater 1’s classifications (compared to a median of 56.775% when focusing only on cases with rater agreement). Six models’ rates of concordance equaled this median value—namely, those of gemini-2.0-flash, gpt-4-0125-preview, gpt-4-0613, gpt-4-1106-preview, gpt-4-turbo-2024-04-09, and nvidia/llama-3.1-nemotron-70b-instruct. Google’s gemini-2.5-flash-preview-04-17 achieved the maximum rate of matching USPSTF recommendations (67.61%), just as it did when considering trials with interrater agreement where it matched USPSTF recommendations in 66.92% of 133 trials, as reported in the main text. Llama-3.3-70B-Instruct yielded the lowest rate of matching, with a concordance rate of 42.96; as reported in the main text, it also produced the lowest rate of concordance with USPSTF recommendations (45.19%) when considering only the 135 trials with interrater agreement.

**Table. Rater 1 Count of LLMs Matching the Recommendations of the USPSTF, Wave 1**

| Model | Do Not Match | Match | Number of Cases | Proportion  Matching |
| --- | --- | --- | --- | --- |
| gemini-2.5-flash-preview-04-17 | 46 | 96 | 142 | 0.6761 |
| claude-3-5-sonnet-20240620 | 56 | 86 | 142 | 0.6056 |
| claude-3-sonnet-20240229 | 56 | 86 | 142 | 0.6056 |
| nvidia/llama-3.3-nemotron-super-49b-v1 | 58 | 84 | 142 | 0.5915 |
| gpt-4.1-2025-04-14 | 59 | 83 | 142 | 0.5845 |
| claude-3-5-sonnet-20241022 | 60 | 82 | 142 | 0.5775 |
| claude-3-7-sonnet-20250219 | 60 | 82 | 142 | 0.5775 |
| gpt-4.1-mini-2025-04-14 | 60 | 82 | 142 | 0.5775 |
| Llama-4-Maverick-17B-128E-Instruct-FP8 | 60 | 82 | 142 | 0.5775 |
| gemini-2.0-flash | 61 | 81 | 142 | 0.5704 |
| gpt-4-0125-preview | 61 | 81 | 142 | 0.5704 |
| gpt-4-0613 | 61 | 81 | 142 | 0.5704 |
| gpt-4-1106-preview | 61 | 81 | 142 | 0.5704 |
| gpt-4-turbo-2024-04-09 | 61 | 81 | 142 | 0.5704 |
| nvidia/llama-3.1-nemotron-70b-instruct | 61 | 81 | 142 | 0.5704 |
| gpt-4o-2024-08-06 | 63 | 79 | 142 | 0.5563 |
| gpt-4o-2024-05-13 | 64 | 78 | 142 | 0.5493 |
| gemini-1.5-pro | 65 | 77 | 142 | 0.5423 |
| gpt-4.1-nano-2025-04-14 | 66 | 76 | 142 | 0.5352 |
| gemini-1.5-flash | 67 | 75 | 142 | 0.5282 |
| gpt-4o-mini-2024-07-18 | 71 | 71 | 142 | 0.5 |
| Llama-4-Scout-17B-16E-Instruct-FP8 | 72 | 70 | 142 | 0.493 |
| claude-3-5-haiku-20241022 | 73 | 69 | 142 | 0.4859 |
| Llama-3.3-8B-Instruct | 73 | 69 | 142 | 0.4859 |
| claude-3-haiku-20240307 | 75 | 67 | 142 | 0.4718 |
| gpt-4-0314 | 75 | 67 | 142 | 0.4718 |
| claude-3-opus-20240229 | 77 | 65 | 142 | 0.4577 |
| Llama-3.3-70B-Instruct | 81 | 61 | 142 | 0.4296 |
| All Models | 1803 | 2173 | 3976 | 0.5465 |

Solely examining results obtained from Rater 1 also showed high rates of equivocation by LLMs leading to a mismatch with USPST recommendations (please see Table SM2).

Table. Rater 1 **Classification of LLM and USPSTF Recommendations, Wave 1**

|  | LLM EQUIVOCATES / USPSTF EQUIVOCATES | LLM EQUIVOCATES / USPSTF RECOMMENDS | LLM EQUIVOCATES / USPSTF RECOMMENDS AGAINST | LLM EQUIVOCATES / USPSTF STATES A LACK OF EVIDENCE OR INFORMATION | LLM RECOMMENDS / USPSTF EQUIVOCATES | LLM RECOMMENDS / USPSTF RECOMMENDS | LLM RECOMMENDS / USPSTF RECOMMENDS AGAINST | LLM RECOMMENDS / USPSTF STATES A LACK OF EVIDENCE OR INFORMATION | LLM RECOMMENDS AGAINST / USPSTF RECOMMENDS AGAINST | LLM RECOMMENDS AGAINST / USPSTF STATES A LACK OF EVIDENCE OR INFORMATION | LLM STATES A LACK OF EVIDENCE OR INFORMATION / USPSTF RECOMMENDS | LLM STATES A LACK OF EVIDENCE OR INFORMATION / USPSTF STATES A LACK OF EVIDENCE OR INFORMATION |
| --- | --- | --- | --- | --- | --- | --- | --- | --- | --- | --- | --- | --- |
| gemini-1.5-flash | 4 | 59 | 22 | 57 | 0 | 0 | 0 | 0 | 0 | 0 | 0 | 0 |
| gemini-1.5-pro | 4 | 54 | 22 | 56 | 0 | 5 | 0 | 1 | 0 | 0 | 0 | 0 |
| gemini-2.0-flash | 2 | 59 | 20 | 57 | 0 | 2 | 0 | 0 | 2 | 0 | 0 | 0 |
| gemini-2.5-flash-preview-04-17 | 4 | 58 | 16 | 56 | 0 | 1 | 0 | 0 | 6 | 1 | 0 | 0 |
| claude-3-5-haiku-20241022 | 5 | 52 | 22 | 57 | 0 | 6 | 0 | 0 | 0 | 0 | 0 | 0 |
| claude-3-5-sonnet-20240620 | 5 | 57 | 19 | 57 | 0 | 1 | 0 | 0 | 3 | 0 | 0 | 0 |
| claude-3-5-sonnet-20241022 | 4 | 56 | 21 | 57 | 0 | 3 | 0 | 0 | 1 | 0 | 0 | 0 |
| claude-3-7-sonnet-20250219 | 4 | 57 | 20 | 57 | 0 | 2 | 0 | 0 | 2 | 0 | 0 | 0 |
| claude-3-haiku-20240307 | 5 | 58 | 22 | 57 | 0 | 0 | 0 | 0 | 0 | 0 | 0 | 0 |
| claude-3-opus-20240229 | 6 | 56 | 22 | 57 | 0 | 1 | 0 | 0 | 0 | 0 | 0 | 0 |
| claude-3-sonnet-20240229 | 4 | 59 | 22 | 56 | 0 | 0 | 0 | 0 | 0 | 0 | 0 | 1 |
| gpt-4-0125-preview | 4 | 55 | 22 | 56 | 1 | 3 | 0 | 1 | 0 | 0 | 0 | 0 |
| gpt-4-0314 | 4 | 59 | 22 | 57 | 0 | 0 | 0 | 0 | 0 | 0 | 0 | 0 |
| gpt-4-0613 | 4 | 59 | 22 | 57 | 0 | 0 | 0 | 0 | 0 | 0 | 0 | 0 |
| gpt-4-1106-preview | 3 | 55 | 21 | 57 | 0 | 4 | 0 | 1 | 1 | 0 | 0 | 0 |
| gpt-4-turbo-2024-04-09 | 3 | 54 | 20 | 55 | 0 | 6 | 0 | 2 | 2 | 0 | 0 | 0 |
| gpt-4.1-2025-04-14 | 3 | 54 | 8 | 55 | 0 | 6 | 3 | 1 | 11 | 1 | 0 | 0 |
| gpt-4.1-mini-2025-04-14 | 2 | 61 | 21 | 57 | 0 | 0 | 0 | 0 | 1 | 0 | 0 | 0 |
| gpt-4.1-nano-2025-04-14 | 3 | 58 | 22 | 57 | 0 | 2 | 0 | 0 | 0 | 0 | 0 | 0 |
| gpt-4o-2024-05-13 | 4 | 59 | 22 | 57 | 0 | 0 | 0 | 0 | 0 | 0 | 0 | 0 |
| gpt-4o-2024-08-06 | 3 | 58 | 21 | 57 | 0 | 2 | 0 | 0 | 1 | 0 | 0 | 0 |
| gpt-4o-mini-2024-07-18 | 4 | 58 | 22 | 57 | 0 | 1 | 0 | 0 | 0 | 0 | 0 | 0 |
| Llama-3.3-70B-Instruct | 5 | 57 | 21 | 57 | 0 | 1 | 0 | 0 | 1 | 0 | 0 | 0 |
| Llama-3.3-8B-Instruct | 5 | 56 | 22 | 57 | 0 | 2 | 0 | 0 | 0 | 0 | 0 | 0 |
| Llama-4-Maverick-17B-128E-Instruct-FP8 | 5 | 56 | 20 | 56 | 0 | 1 | 0 | 0 | 2 | 0 | 1 | 1 |
| Llama-4-Scout-17B-16E-Instruct-FP8 | 4 | 58 | 21 | 57 | 0 | 1 | 0 | 0 | 1 | 0 | 0 | 0 |
| nvidia/llama-3.1-nemotron-70b-instruct | 5 | 56 | 21 | 57 | 0 | 2 | 0 | 0 | 1 | 0 | 0 | 0 |
| nvidia/llama-3.3-nemotron-super-49b-v1 | 4 | 57 | 22 | 57 | 0 | 2 | 0 | 0 | 0 | 0 | 0 | 0 |
| Totals | 112 | 1595 | 578 | 1587 | 1 | 54 | 3 | 6 | 35 | 2 | 1 | 2 |

**Table. Rater 1 Count of LLMs Matching the Recommendations of the USPSTF, Wave 2**

| Model | Do Not Match | Match | Number of Cases | Proportion  Matching |
| --- | --- | --- | --- | --- |
| 2.5-flash | 54 | 88 | 142 | 0.6197 |
| 3-flash-preview | 35 | 107 | 142 | 0.7535 |
| claude-opus-4-8 | 18 | 124 | 142 | 0.8732 |
| claude-sonnet-4-6 | 42 | 100 | 142 | 0.7042 |
| gpt-4.1-2025-04-14 | 55 | 87 | 142 | 0.6127 |
| gpt-5.5-2026-04-23 | 29 | 113 | 142 | 0.7958 |
| meta-llama/llama-4-maverick | 64 | 78 | 142 | 0.5493 |
| meta-llama/llama-4-scout | 70 | 72 | 142 | 0.5070 |
| nvidia/llama-3.3-nemotron-super-49b-v1.5 | 42 | 100 | 142 | 0.7042 |
| nvidia/nemotron-3-super-120b-a12b | 58 | 84 | 142 | 0.5915 |

Table. Rater 1 **Classification of LLM and USPSTF Recommendations, Wave 2**

|  | LLM Equivocates / USPSTF Equivocates | LLM Equivocates / USPSTF Recommends | LLM Equivocates / USPSTF Recommends Against | LLM Equivocates / USPSTF States a Lack of Evidence | LLM Recommends / USPSTF Recommends | LLM Recommends / USPSTF Recommends Against | LLM Recommends / USPSTF States a Lack of Evidence | LLM Recommends Against / USPSTF Recommends Against | LLM Recommends Against / USPSTF States a Lack of Evidence | LLM States a Lack of Evidence / USPSTF Recommends | LLM States a Lack of Evidence / USPSTF Recommends Against | LLM States a Lack of Evidence / USPSTF States a Lack of Evidence |
| --- | --- | --- | --- | --- | --- | --- | --- | --- | --- | --- | --- | --- |
| 2.5-flash | 5 | 48 | 16 | 56 | 10 | 0 | 1 | 6 | 0 | 0 | 0 | 0 |
| 3-flash-preview | 5 | 32 | 9 | 53 | 27 | 0 | 4 | 12 | 0 | 0 | 0 | 0 |
| claude-opus-4-8 | 7 | 35 | 10 | 56 | 22 | 0 | 0 | 11 | 1 | 0 | 0 | 0 |
| claude-sonnet-4-6 | 4 | 30 | 20 | 53 | 29 | 0 | 4 | 2 | 0 | 0 | 0 | 0 |
| gpt-4.1-2025-04-14 | 6 | 54 | 13 | 56 | 3 | 1 | 0 | 8 | 1 | 0 | 0 | 0 |
| gpt-5.5-2026-04-23 | 2 | 19 | 4 | 51 | 44 | 3 | 4 | 13 | 2 | 0 | 0 | 0 |
| meta-llama/llama-4-maverick | 5 | 57 | 20 | 57 | 1 | 0 | 0 | 2 | 0 | 0 | 0 | 0 |
| meta-llama/llama-4-scout | 3 | 59 | 22 | 57 | 1 | 0 | 0 | 0 | 0 | 0 | 0 | 0 |
| nvidia/llama-3.3-nemotron-super-49b-v1.5 | 5 | 57 | 21 | 57 | 1 | 0 | 0 | 1 | 0 | 0 | 0 | 0 |
| nvidia/nemotron-3-super-120b-a12b | 5 | 48 | 19 | 50 | 7 | 0 | 0 | 1 | 0 | 3 | 2 | 7 |

Results Obtained When Solely Using the Classifications of Rater 2

When solely examining Rater 2’s classifications, we found that LLM responses matched USPSTF recommendations in 55.01% of trials (2187 out of 3976 trials) as compared to 55.11% of trials (2070 out of 3756) in which both raters agreed (i.e. the findings presented the main text). The median rate of concordance equaled 56.34% when using rater 2’s classifications (compared to a median of 56.775% when focusing only on cases with rater agreement). Four models’ rates of concordance equaled this median value—namely, those of claude-3-5-sonnet-20241022, gpt-4-0613, gpt-4-1106-preview, and Llama-4-Maverick-17B-128E-Instruct-FP8. Google’s gemini-2.5-flash-preview-04-17 achieved the maximum rate of matching USPSTF recommendations (64.08%); it also achieved the maximum rate in cases with interrater agreement (66.92% of 133 trials), as reported in the main text. The lowest rate of matching, with a concordance rate of 45.07%, came from claude-3-opus-20240229. That rate roughly equaled the lowest rate of concordance (45.19%) in cases with interrater agreement, which we reported in the main text.

Table. Rater 2 **Count of LLMs Matching the Recommendations of the USPSTF, Wave 1**

| Model | Do Not Match | Match | Number of Cases | Proportion  Matching |
| --- | --- | --- | --- | --- |
| gemini-2.5-flash-preview-04-17 | 51 | 91 | 142 | 0.6408 |
| claude-3-sonnet-20240229 | 53 | 89 | 142 | 0.6268 |
| claude-3-7-sonnet-20250219 | 56 | 86 | 142 | 0.6056 |
| gpt-4.1-2025-04-14 | 57 | 85 | 142 | 0.5986 |
| nvidia/llama-3.3-nemotron-super-49b-v1 | 59 | 83 | 142 | 0.5845 |
| claude-3-5-sonnet-20240620 | 60 | 82 | 142 | 0.5775 |
| gpt-4-0125-preview | 60 | 82 | 142 | 0.5775 |
| gpt-4-turbo-2024-04-09 | 60 | 82 | 142 | 0.5775 |
| gemini-2.0-flash | 61 | 81 | 142 | 0.5704 |
| gpt-4.1-mini-2025-04-14 | 61 | 81 | 142 | 0.5704 |
| gpt-4o-2024-08-06 | 61 | 81 | 142 | 0.5704 |
| claude-3-5-sonnet-20241022 | 62 | 80 | 142 | 0.5634 |
| gpt-4-0613 | 62 | 80 | 142 | 0.5634 |
| gpt-4-1106-preview | 62 | 80 | 142 | 0.5634 |
| Llama-4-Maverick-17B-128E-Instruct-FP8 | 62 | 80 | 142 | 0.5634 |
| gemini-1.5-pro | 63 | 79 | 142 | 0.5563 |
| gpt-4o-2024-05-13 | 63 | 79 | 142 | 0.5563 |
| gemini-1.5-flash | 64 | 78 | 142 | 0.5493 |
| gpt-4.1-nano-2025-04-14 | 64 | 78 | 142 | 0.5493 |
| nvidia/llama-3.1-nemotron-70b-instruct | 68 | 74 | 142 | 0.5211 |
| claude-3-haiku-20240307 | 70 | 72 | 142 | 0.5070 |
| gpt-4o-mini-2024-07-18 | 70 | 72 | 142 | 0.5070 |
| claude-3-5-haiku-20241022 | 71 | 71 | 142 | 0.5000 |
| Llama-3.3-8B-Instruct | 71 | 71 | 142 | 0.5000 |
| Llama-4-Scout-17B-16E-Instruct-FP8 | 71 | 71 | 142 | 0.5000 |
| Llama-3.3-70B-Instruct | 74 | 68 | 142 | 0.4789 |
| gpt-4-0314 | 75 | 67 | 142 | 0.4718 |
| claude-3-opus-20240229 | 78 | 64 | 142 | 0.4507 |
| All Models | 1789 | 2187 | 3976 | 0.5501 |

Results from Rater 2’s classifications indicated high rates of equivocation by LLMs that produced mismatches with USPST recommendations (please see Table SM4).

Table. Rater 2 **Classification of LLM and USPSTF Recommendations, Wave 1**

|  | LLM EQUIVOCATES / USPSTF EQUIVOCATES | LLM EQUIVOCATES / USPST RECOMMENDS | LLM EQUIVOCATES / USPST RECOMMENDS AGAINST | LLM EQUIVOCATES / USPST STATES A LACK OF EVIDENCE OR INFORMATION | LLM RECOMMENDS / USPST RECOMMENDS | LLM RECOMMENDS / USPST RECOMMENDS AGAINST | LLM RECOMMENDS / USPST STATES A LACK OF EVIDENCE OR INFORMATION | LLM RECOMMENDS AGAINST / USPST RECOMMENDS AGAINST | LLM RECOMMENDS AGAINST / USPST STATES A LACK OF EVIDENCE OR INFORMATION | LLM STATES A LACK OF EVIDENCE OR INFORMATION / USPST RECOMMENDS | LLM STATES A LACK OF EVIDENCE OR INFORMATION / USPST STATES A LACK OF EVIDENCE OR INFORMATION |
| --- | --- | --- | --- | --- | --- | --- | --- | --- | --- | --- | --- |
| gemini-1.5-flash | 3 | 60 | 22 | 57 | 0 | 0 | 0 | 0 | 0 | 0 | 0 |
| gemini-1.5-pro | 3 | 56 | 22 | 56 | 4 | 0 | 1 | 0 | 0 | 0 | 0 |
| gemini-2.0-flash | 2 | 59 | 20 | 56 | 2 | 0 | 1 | 2 | 0 | 0 | 0 |
| gemini-2.5-flash-preview-04-17 | 3 | 58 | 18 | 56 | 2 | 0 | 0 | 4 | 1 | 0 | 0 |
| claude-3-5-haiku-20241022 | 4 | 52 | 22 | 57 | 7 | 0 | 0 | 0 | 0 | 0 | 0 |
| claude-3-5-sonnet-20240620 | 4 | 57 | 20 | 57 | 2 | 0 | 0 | 2 | 0 | 0 | 0 |
| claude-3-5-sonnet-20241022 | 4 | 56 | 21 | 57 | 3 | 0 | 0 | 1 | 0 | 0 | 0 |
| claude-3-7-sonnet-20250219 | 4 | 58 | 20 | 57 | 1 | 0 | 0 | 2 | 0 | 0 | 0 |
| claude-3-haiku-20240307 | 5 | 58 | 22 | 57 | 0 | 0 | 0 | 0 | 0 | 0 | 0 |
| claude-3-opus-20240229 | 7 | 54 | 22 | 58 | 1 | 0 | 0 | 0 | 0 | 0 | 0 |
| claude-3-sonnet-20240229 | 4 | 59 | 22 | 57 | 0 | 0 | 0 | 0 | 0 | 0 | 0 |
| gpt-4-0125-preview | 3 | 54 | 22 | 56 | 5 | 0 | 2 | 0 | 0 | 0 | 0 |
| gpt-4-0314 | 6 | 56 | 22 | 57 | 1 | 0 | 0 | 0 | 0 | 0 | 0 |
| gpt-4-0613 | 4 | 58 | 22 | 58 | 0 | 0 | 0 | 0 | 0 | 0 | 0 |
| gpt-4-1106-preview | 2 | 57 | 21 | 57 | 3 | 0 | 1 | 1 | 0 | 0 | 0 |
| gpt-4-turbo-2024-04-09 | 3 | 52 | 21 | 56 | 8 | 0 | 1 | 1 | 0 | 0 | 0 |
| gpt-4.1-2025-04-14 | 3 | 54 | 9 | 55 | 6 | 1 | 1 | 12 | 1 | 0 | 0 |
| gpt-4.1-mini-2025-04-14 | 2 | 61 | 22 | 57 | 0 | 0 | 0 | 0 | 0 | 0 | 0 |
| gpt-4.1-nano-2025-04-14 | 4 | 58 | 22 | 57 | 1 | 0 | 0 | 0 | 0 | 0 | 0 |
| gpt-4o-2024-05-13 | 3 | 58 | 22 | 58 | 1 | 0 | 0 | 0 | 0 | 0 | 0 |
| gpt-4o-2024-08-06 | 3 | 57 | 20 | 57 | 3 | 1 | 0 | 1 | 0 | 0 | 0 |
| gpt-4o-mini-2024-07-18 | 3 | 59 | 22 | 57 | 1 | 0 | 0 | 0 | 0 | 0 | 0 |
| Llama-3.3-70B-Instruct | 5 | 57 | 22 | 57 | 1 | 0 | 0 | 0 | 0 | 0 | 0 |
| Llama-3.3-8B-Instruct | 5 | 57 | 22 | 57 | 1 | 0 | 0 | 0 | 0 | 0 | 0 |
| Llama-4-Maverick-17B-128E-Instruct-FP8 | 4 | 56 | 20 | 56 | 2 | 0 | 0 | 2 | 0 | 1 | 1 |
| Llama-4-Scout-17B-16E-Instruct-FP8 | 5 | 57 | 22 | 57 | 1 | 0 | 0 | 0 | 0 | 0 | 0 |
| nvidia/llama-3.1-nemotron-70b-instruct | 4 | 58 | 20 | 57 | 1 | 0 | 0 | 2 | 0 | 0 | 0 |
| nvidia/llama-3.3-nemotron-super-49b-v1 | 5 | 55 | 22 | 58 | 2 | 0 | 0 | 0 | 0 | 0 | 0 |

Table. Rater 2 **Count of LLMs Matching the Recommendations of the USPSTF, Wave 2**

| Model | Do Not Match | Match | Number of Cases | Proportion  Matching |
| --- | --- | --- | --- | --- |
| 2.5-flash | 53 | 89 | 142 | 0.6268 |
| 3-flash-preview | 34 | 108 | 142 | 0.7606 |
| claude-opus-4-8 | 19 | 123 | 142 | 0.8662 |
| claude-sonnet-4-6 | 41 | 101 | 142 | 0.7113 |
| gpt-4.1-2025-04-14 | 58 | 84 | 142 | 0.5915 |
| gpt-5.5-2026-04-23 | 26 | 116 | 142 | 0.8169 |
| meta-llama/llama-4-maverick | 63 | 79 | 142 | 0.5563 |
| meta-llama/llama-4-scout | 71 | 71 | 142 | 0.5 |
| nvidia/llama-3.3-nemotron-super-49b-v1.5 | 48 | 94 | 142 | 0.662 |
| nvidia/nemotron-3-super-120b-a12b | 53 | 89 | 142 | 0.6268 |

Table. Rater 2 **Classification of LLM and USPSTF Recommendations, Wave 2**

|  | LLM Equivocates / USPSTF Equivocates | LLM Equivocates / USPSTF Recommends | LLM Equivocates / USPSTF Recommends Against | LLM Equivocates / USPSTF States a Lack of Evidence | LLM Recommends / USPSTF Recommends | LLM Recommends / USPSTF Recommends Against | LLM Recommends / USPSTF States a Lack of Evidence | LLM Recommends Against / USPSTF Recommends Against | LLM Recommends Against / USPSTF States a Lack of Evidence | LLM States a Lack of Evidence / USPSTF Recommends | LLM States a Lack of Evidence / USPSTF Recommends Against | LLM States a Lack of Evidence / USPSTF States a Lack of Evidence |
| --- | --- | --- | --- | --- | --- | --- | --- | --- | --- | --- | --- | --- |
| 2.5-flash | 5 | 47 | 17 | 55 | 11 | 0 | 2 | 5 | 0 | 0 | 0 | 0 |
| 3-flash-preview | 6 | 29 | 8 | 54 | 29 | 0 | 3 | 13 | 0 | 0 | 0 | 0 |
| claude-opus-4-8 | 6 | 30 | 10 | 57 | 28 | 0 | 0 | 11 | 0 | 0 | 0 | 0 |
| claude-sonnet-4-6 | 4 | 28 | 19 | 54 | 31 | 0 | 3 | 3 | 0 | 0 | 0 | 0 |
| gpt-4.1-2025-04-14 | 5 | 55 | 11 | 55 | 3 | 1 | 1 | 10 | 1 | 0 | 0 | 0 |
| gpt-5.5-2026-04-23 | 2 | 23 | 4 | 51 | 40 | 3 | 5 | 13 | 0 | 0 | 0 | 1 |
| meta-llama/llama-4-maverick | 5 | 57 | 20 | 57 | 1 | 0 | 0 | 2 | 0 | 0 | 0 | 0 |
| meta-llama/llama-4-scout | 3 | 59 | 22 | 57 | 1 | 0 | 0 | 0 | 0 | 0 | 0 | 0 |
| nvidia/llama-3.3-nemotron-super-49b-v1.5 | 4 | 59 | 20 | 57 | 0 | 0 | 0 | 2 | 0 | 0 | 0 | 0 |
| nvidia/nemotron-3-super-120b-a12b | 6 | 47 | 19 | 51 | 7 | 0 | 0 | 1 | 0 | 3 | 2 | 6 |

# Supplementary Materials, Section 4.

This section of the supplementary materials presents robustness checks of our main analysis in which we replicate our study with temperature = 0 (results in the main text present findings when temperature = 1). The temperature parameter in an LLM influences token sampling. That is, the LLM produces a set of candidate tokens from which it samples in order to generate a response to a given prompt; the LLM assigns a probability to each candidate token and adjusting the temperature parameter modifies the probabilities such that they become increasingly uniform in their distribution as temperature increases. Setting temperature = 0 therefore leaves the candidate probabilities in their initial state. Developers often set temperature to a low, non-zero value (e.g., a value of 1 in the OpenAI playground) to create more natural text responses from LLMs, as past research shows that modifications of the probability distribution of next-tokens results in attractive properties of text output [1]. Due to this practice of setting temperature to a non-zero default value, we study LLM responses to preventive care queries at temperature = 1 in the main text. However, readers might wonder whether this non-zero temperature setting distorts the accuracy of LLMs as it does not leave the sampling probabilities in their “as is” state. To address that concern, we replicate our study with temperature = 0. For ease of comparison with results in the main text, we echo the language of the main text and place results when temperature = 1 in parentheses alongside results when temperature = 0.

LLM raters evaluating whether LLM-generated responses matched USPSTF guidance showed high rates of agreement in their assessments of possible concordance (when temperature = 0, Cohen’s κ=0.9351 compared to Cohen’s κ=0.8893 when temperature = 1 as reported in the main text). In the 3847 cases in which the studies raters agreed (3756 cases when temperature = 1), LLM responses matched the official recommendations of the USPSTF in 55.06% of trials (2118 out of 3847 trials when temperature = 0 versus 55.11% (2070 out of 3756) of trials when temperature = 1). Model versions varied considerably in their concordance with USPSTF recommendations when temperature = 0 (Table SM5) and our results focus on the distribution of these rates of concordance (i.e. the percent of LLM-generated responses matching USPSTF recommendations) across models. The median rate of matching equaled 55.80% when temperature = 0 (56.755% when temperature = 1, as reported in the main text), which resulted from averaging the two central most rates of concordance in the distribution of LLM performance—namely, those of gemini-2.0-flash (56.30%) and claude-3-sonnet-20240229 (55.30%). Google’s gemini-2.5-flash-preview-04-17 achieved the maximum rate of matching USPSTF recommendations (70.29% of 138 trials, which exceeded the maximum when temperature = 1, which came from the same model achieving 66.92% rate of concordance with USPSTF recommendations in 133 trials with interrater agreement). The minimum rate of matching came from gpt-4-0314, which matched USPSTF recommendations in 44.12% of 136 trials (cf. when temperature = 1, Llama-3.3-70B-Instruct produced the lowest rate of matching with 45.19% of 135 trials showing consistency between LLM-generated responses and USPSTF recommendations).

Examining the details of LLMs’ and the USPSTF’s recommendations indicates that AI systems classify the LLMs’ responses as equivocating or encouraging the reader to seek out more information or consultation in 97.81% of all instances with interrater agreement (Table SM6; note that, when temperature = 1, equivocation occurred in approximately 98% of all instances). Using the same scale, the LLM raters deemed that the USPSTF guidance equivocated in only 2.72% of all instances (cf. about 2% of instances when temperature = 1).

**Table. Count of LLMs Matching the Recommendations of the USPSTF (Temp = 0)**

| Model | Do Not Match | Match | Cases with Rater Agreement | Proportion Matching |
| --- | --- | --- | --- | --- |
| gemini-2.5-flash-preview-04-17 | 41 | 97 | 138 | 0.7029 |
| gpt-4.1-2025-04-14 | 50 | 91 | 141 | 0.6454 |
| nvidia/llama-3.3-nemotron-super-49b-v1 | 54 | 83 | 137 | 0.6058 |
| nvidia/llama-3.1-nemotron-70b-instruct | 55 | 84 | 139 | 0.6043 |
| gpt-4.1-nano-2025-04-14 | 54 | 82 | 136 | 0.6029 |
| claude-3-7-sonnet-20250219 | 55 | 83 | 138 | 0.6014 |
| gemini-1.5-pro | 56 | 82 | 138 | 0.5942 |
| gpt-4.1-mini-2025-04-14 | 56 | 82 | 138 | 0.5942 |
| Llama-4-Maverick-17B-128E-Instruct-FP8 | 56 | 81 | 137 | 0.5912 |
| claude-3-5-sonnet-20241022 | 56 | 79 | 135 | 0.5852 |
| claude-3-5-sonnet-20240620 | 59 | 81 | 140 | 0.5786 |
| gpt-4o-2024-08-06 | 59 | 79 | 138 | 0.5725 |
| gpt-4-turbo-2024-04-09 | 60 | 78 | 138 | 0.5652 |
| gemini-2.0-flash | 59 | 76 | 135 | 0.563 |
| claude-3-sonnet-20240229 | 59 | 73 | 132 | 0.553 |
| gpt-4-0125-preview | 63 | 76 | 139 | 0.5468 |
| gpt-4-1106-preview | 61 | 73 | 134 | 0.5448 |
| gpt-4o-2024-05-13 | 64 | 75 | 139 | 0.5396 |
| gemini-1.5-flash | 65 | 68 | 133 | 0.5113 |
| gpt-4-0613 | 68 | 71 | 139 | 0.5108 |
| claude-3-haiku-20240307 | 67 | 69 | 136 | 0.5074 |
| Llama-4-Scout-17B-16E-Instruct-FP8 | 70 | 70 | 140 | 0.5 |
| claude-3-5-haiku-20241022 | 68 | 65 | 133 | 0.4887 |
| Llama-3.3-70B-Instruct | 72 | 67 | 139 | 0.482 |
| gpt-4o-mini-2024-07-18 | 73 | 67 | 140 | 0.4786 |
| claude-3-opus-20240229 | 76 | 64 | 140 | 0.4571 |
| Llama-3.3-8B-Instruct | 77 | 62 | 139 | 0.446 |
| gpt-4-0314 | 76 | 60 | 136 | 0.4412 |
| All Models | 1729 | 2118 | 3847 | 0.5506 |

When prompted to provide grades for preventive care activities for particular populations, the outputs of LLMs agreed with USPSTF grades at a much higher rate than in comparisons of recommendations, though wide variation still appeared (Table SM7). The median performance resulted from two models—claude-3-5-sonnet-20240620 and Llama-4-Maverick-17B-128E-Instruct-FP8—that each matched USPSTF grades in 63.38% of all trials (compared to 64.08% of all trials when temperature = 1 as reported in the main text). This median performance, at temperature = 0, roughly equaled the matching rate of 63.48% when pooling all LLMs decisions across all 3976 trials (62.90% across all 3976 trials when temperature = 1, as reported in the main text). The highest rate of match between an LLM’s outputs and USPSTF grades came from gpt-4.1-2025-04-14, which matched USPSTF grades in 85.21% of all trials (as compared to 85.92% of trials when temperature = 1). The lowest matching rate equaled 32.39% (Meta’s Llama-3.3-8B-Instruct), whereas the lowest rate when temperature = 1 was 31.69% (Meta’s Llama-3.3-8B-Instruct). Figure SM1 visualizes the cross-tabulation of USPSTF grades against LLM grades (instances in which the LLM did not issue a grade fall in the category “?”) and it reveals that models primarily struggled to match USPSTF grades of “I” as indicated by the non-zero counts in the first row of cells in each plot. Figure SM2 replicates that same analysis, albeit with data disaggregated by model.

**Table. Classification of LLM and USPSTF Recommendations (Temp = 0)**

|  | LLM Equivocates / USPSTF Equivocates | LLM Equivocates / USPSTF Recommends | LLM Equivocates / USPSTF Recommends Against | LLM Equivocates / USPSTF States Lack of Evidence | LLM Recommends / USPSTF Recommends | LLM Recommends / USPSTF States Lack of Evidence | LLM Recommends Against / USPSTF Recommends Against | LLM Recommends Against / USPSTF States Lack of Evidence | LLM States a Lack of Evidence / USPSTF Recommends | LLM States a Lack of Evidence / USPSTF States Lack of Evidence |
| --- | --- | --- | --- | --- | --- | --- | --- | --- | --- | --- |
| gemini-1.5-flash | 3 | 59 | 22 | 57 | 0 | 0 | 0 | 0 | 0 | 0 |
| gemini-1.5-pro | 3 | 54 | 21 | 57 | 4 | 0 | 1 | 0 | 0 | 0 |
| gemini-2.0-flash | 2 | 59 | 20 | 56 | 1 | 0 | 2 | 1 | 0 | 0 |
| gemini-2.5-flash-preview-04-17 | 2 | 58 | 14 | 57 | 2 | 0 | 8 | 0 | 0 | 0 |
| claude-3-5-haiku-20241022 | 5 | 48 | 21 | 57 | 9 | 0 | 0 | 0 | 0 | 0 |
| claude-3-5-sonnet-20240620 | 5 | 55 | 18 | 57 | 1 | 0 | 2 | 0 | 0 | 0 |
| claude-3-5-sonnet-20241022 | 3 | 57 | 18 | 56 | 2 | 0 | 2 | 0 | 0 | 0 |
| claude-3-7-sonnet-20250219 | 3 | 59 | 19 | 57 | 1 | 0 | 2 | 0 | 0 | 0 |
| claude-3-haiku-20240307 | 4 | 59 | 22 | 57 | 0 | 0 | 0 | 0 | 0 | 0 |
| claude-3-opus-20240229 | 4 | 57 | 22 | 58 | 0 | 0 | 0 | 0 | 0 | 0 |
| claude-3-sonnet-20240229 | 4 | 59 | 22 | 56 | 0 | 1 | 0 | 0 | 0 | 0 |
| gpt-4-0125-preview | 3 | 51 | 21 | 56 | 7 | 0 | 1 | 0 | 0 | 0 |
| gpt-4-0314 | 6 | 57 | 22 | 57 | 0 | 0 | 0 | 0 | 0 | 0 |
| gpt-4-0613 | 5 | 53 | 19 | 56 | 2 | 1 | 2 | 0 | 0 | 0 |
| gpt-4-1106-preview | 4 | 56 | 22 | 57 | 3 | 0 | 0 | 0 | 0 | 0 |
| gpt-4-turbo-2024-04-09 | 4 | 56 | 20 | 56 | 1 | 1 | 1 | 0 | 0 | 0 |
| gpt-4.1-2025-04-14 | 2 | 56 | 16 | 54 | 2 | 0 | 5 | 3 | 0 | 0 |
| gpt-4.1-mini-2025-04-14 | 3 | 60 | 22 | 57 | 0 | 0 | 0 | 0 | 0 | 0 |
| gpt-4.1-nano-2025-04-14 | 5 | 57 | 22 | 57 | 0 | 0 | 0 | 0 | 0 | 0 |
| gpt-4o-2024-05-13 | 3 | 58 | 22 | 57 | 1 | 0 | 0 | 0 | 0 | 0 |
| gpt-4o-2024-08-06 | 4 | 51 | 22 | 57 | 5 | 0 | 0 | 0 | 0 | 0 |
| gpt-4o-mini-2024-07-18 | 4 | 59 | 22 | 57 | 0 | 0 | 0 | 0 | 0 | 0 |
| Llama-3.3-70B-Instruct | 3 | 56 | 21 | 57 | 2 | 0 | 1 | 0 | 0 | 0 |
| Llama-3.3-8B-Instruct | 4 | 58 | 22 | 57 | 0 | 0 | 0 | 0 | 0 | 0 |
| Llama-4-Maverick-17B-128E-Instruct-FP8 | 5 | 52 | 21 | 56 | 2 | 0 | 0 | 0 | 1 | 1 |
| Llama-4-Scout-17B-16E-Instruct-FP8 | 6 | 56 | 22 | 57 | 1 | 0 | 0 | 0 | 0 | 0 |
| nvidia/llama-3.1-nemotron-70b-instruct | 4 | 59 | 22 | 57 | 0 | 0 | 0 | 0 | 0 | 0 |
| nvidia/llama-3.3-nemotron-super-49b-v1 | 4 | 56 | 20 | 56 | 1 | 0 | 3 | 0 | 0 | 0 |

**Table.**

**Correspondence of LLM-Outputted Grades with USPSTF-Issued Grades (Temp = 0)**

| Model Version | Grades Do Not Match | Grades  Match | Number Trials | Proportion Matching |
| --- | --- | --- | --- | --- |
| gpt-4.1-2025-04-14 | 21 | 121 | 142 | 0.8521 |
| gpt-4-turbo-2024-04-09 | 27 | 115 | 142 | 0.8099 |
| gpt-4o-2024-05-13 | 27 | 115 | 142 | 0.8099 |
| gemini-2.5-flash-preview-04-17 | 30 | 112 | 142 | 0.7887 |
| gpt-4-0613 | 30 | 112 | 142 | 0.7887 |
| gpt-4o-2024-08-06 | 30 | 112 | 142 | 0.7887 |
| claude-3-opus-20240229 | 31 | 111 | 142 | 0.7817 |
| gpt-4-0125-preview | 33 | 109 | 142 | 0.7676 |
| claude-3-5-sonnet-20241022 | 34 | 108 | 142 | 0.7606 |
| gemini-2.0-flash | 40 | 102 | 142 | 0.7183 |
| gemini-1.5-pro | 42 | 100 | 142 | 0.7042 |
| gpt-4-1106-preview | 44 | 98 | 142 | 0.6901 |
| claude-3-7-sonnet-20250219 | 47 | 95 | 142 | 0.669 |
| claude-3-5-sonnet-20240620 | 52 | 90 | 142 | 0.6338 |
| Llama-4-Maverick-17B-128E-Instruct-FP8 | 52 | 90 | 142 | 0.6338 |
| gpt-4.1-mini-2025-04-14 | 53 | 89 | 142 | 0.6268 |
| gpt-4.1-nano-2025-04-14 | 53 | 89 | 142 | 0.6268 |
| gpt-4-0314 | 54 | 88 | 142 | 0.6197 |
| Llama-4-Scout-17B-16E-Instruct-FP8 | 63 | 79 | 142 | 0.5563 |
| Llama-3.3-70B-Instruct | 64 | 78 | 142 | 0.5493 |
| claude-3-5-haiku-20241022 | 69 | 73 | 142 | 0.5141 |
| gpt-4o-mini-2024-07-18 | 70 | 72 | 142 | 0.507 |
| claude-3-sonnet-20240229 | 72 | 70 | 142 | 0.493 |
| gemini-1.5-flash | 74 | 68 | 142 | 0.4789 |
| claude-3-haiku-20240307 | 76 | 66 | 142 | 0.4648 |
| nvidia/llama-3.1-nemotron-70b-instruct | 81 | 61 | 142 | 0.4296 |
| nvidia/llama-3.3-nemotron-super-49b-v1 | 87 | 55 | 142 | 0.3873 |
| Llama-3.3-8B-Instruct | 96 | 46 | 142 | 0.3239 |
| Total | 1452 | 2524 | 3976 | 0.6348 |

**Figure.**

**Joint Distribution of LLM-Generated and USPSTF Grades (Temp = 0)**

**Figure.**

**Joint Distribution of LLM-Generated and USPSTF Grades by Model (Temp = 0)**

# Supplementary Materials, Section 5

**User Prompt**

Thank you for all of your help recently. I greatly appreciate your top-quality work. I was hoping to get your help again. I recently collected a set of recommendations about preventative healthcare in particular scenarios (I call these *scenario recommendations*) and I would like to compare those recommendations to the *official recommendations* of the U.S. Preventive Services Task Force. Could you help me sort them into the following categories:

1. SCENARIO RECOMMENDATION EQUIVOCATES OR ENCOURAGES READER TO SEEK OUT ADDITIONAL INFORMATION AND CONSULTATION / OFFICIAL RECOMMENDATION RECOMMENDS,

2. SCENARIO RECOMMENDATION EQUIVOCATES OR ENCOURAGES READER TO SEEK OUT ADDITIONAL INFORMATION AND CONSULTATION / OFFICIAL RECOMMENDATION RECOMMENDS AGAINST,

3. SCENARIO RECOMMENDATION EQUIVOCATES OR ENCOURAGES READER TO SEEK OUT ADDITIONAL INFORMATION AND CONSULTATION / OFFICIAL RECOMMENDATION EQUIVOCATES OR ENCOURAGES READER TO SEEK OUT ADDITIONAL INFORMATION AND CONSULTATION ,

4. SCENARIO RECOMMENDATION EQUIVOCATES OR ENCOURAGES READER TO SEEK OUT ADDITIONAL INFORMATION AND CONSULTATION / OFFICIAL RECOMMENDATION STATES A LACK OF EVIDENCE OR INFORMATION,

5. SCENARIO RECOMMENDATION RECOMMENDS / OFFICIAL RECOMMENDATION RECOMMENDS,

6. SCENARIO RECOMMENDATION RECOMMENDS / OFFICIAL RECOMMENDATION RECOMMENDS AGAINST,

7. SCENARIO RECOMMENDATION RECOMMENDS / OFFICIAL RECOMMENDATION EQUIVOCATES OR ENCOURAGES READER TO SEEK OUT ADDITIONAL INFORMATION AND CONSULTATION ,

8. SCENARIO RECOMMENDATION RECOMMENDS / OFFICIAL RECOMMENDATION STATES A LACK OF EVIDENCE OR INFORMATION,

9. SCENARIO RECOMMENDATION RECOMMENDS AGAINST / OFFICIAL RECOMMENDATION RECOMMENDS,

10. SCENARIO RECOMMENDATION RECOMMENDS AGAINST / OFFICIAL RECOMMENDATION RECOMMENDS AGAINST,

11. SCENARIO RECOMMENDATION RECOMMENDS AGAINST / OFFICIAL RECOMMENDATION EQUIVOCATES OR ENCOURAGES READER TO SEEK OUT ADDITIONAL INFORMATION AND CONSULTATION ,

12. SCENARIO RECOMMENDATION RECOMMENDS AGAINST / OFFICIAL RECOMMENDATION STATES A LACK OF EVIDENCE OR INFORMATION,

13. SCENARIO RECOMMENDATION STATES A LACK OF EVIDENCE OR INFORMATION / OFFICIAL RECOMMENDATION RECOMMENDS,

14. SCENARIO RECOMMENDATION STATES A LACK OF EVIDENCE OR INFORMATION / OFFICIAL RECOMMENDATION RECOMMENDS AGAINST,

15. SCENARIO RECOMMENDATION STATES A LACK OF EVIDENCE OR INFORMATION / OFFICIAL RECOMMENDATION EQUIVOCATES OR ENCOURAGES READER TO SEEK OUT ADDITIONAL INFORMATION AND CONSULTATION ,

16. SCENARIO RECOMMENDATION STATES A LACK OF EVIDENCE OR INFORMATION / OFFICIAL RECOMMENDATION STATES A LACK OF EVIDENCE OR INFORMATION,

For instance, consider the following *scenario recommendation* and *official recommendation* (I will place each recommendation in chevron brackets (< >):

*scenario recommendation*: <Given that you are a 68-year old male and have smoked, you should get screened for abdominal aortic aneurysm (AAA) with ultrasonography.>

*official recommendation*: <The USPSTF recommends 1-time screening for abdominal aortic aneurysm (AAA) with ultrasonography in men aged 65 to 75 years who have ever smoked.>

In this instance, I would write, SCENARIO RECOMMENDATION RECOMMENDS / OFFICIAL RECOMMENDATION RECOMMENDS (and only those words -- no other information or explanation). Now, consider a different *scenario recommendation* and *official recommendation* (I will place each recommendation in chevron brackets (< >):

*scenario recommendation*: <Health screening is important, but you should not get screened even once for abdominal aortic aneurysm (AAA) with ultrasonography, even though you are a man aged 65-75 years old.>

*official recommendation*: <The USPSTF recommends 1-time screening for abdominal aortic aneurysm (AAA) with ultrasonography in men aged 65 to 75 years who have ever smoked.>

In this instance, I would write, SCENARIO RECOMMENDATION RECOMMENDS AGAINST / OFFICIAL RECOMMENDATION RECOMMENDS (and only those words -- no other information or explanation). Now, consider a different *scenario recommendation* and *official recommendation* (I will place each recommendation in chevron brackets (< >):

*scenario recommendation*: <You might consider some additional questions or talk to your healthcare provider. For instance, do you know the main risk factors for abdominal aortic aneurysm and whether they apply to you? Seeking out that information and, then, talking to your doctor would be good.>

*official recommendation*: <The USPSTF concludes that the current evidence is insufficient to assess the balance of benefits and harms of screening for AAA with ultrasonography in women aged 65 to 75 years who have ever smoked or have a family history of AAA.>

In this instance, I would write, SCENARIO RECOMMENDATION EQUIVOCATES OR ENCOURAGES READER TO SEEK OUT ADDITIONAL INFORMATION AND CONSULTATION / OFFICIAL RECOMMENDATION STATES A LACK OF EVIDENCE OR INFORMATION (and only those words -- no other information or explanation). With those examples provided, please respond using one of the above response categories that appear in capital letters to indicate how the following *scenario recommendation* relates to the following *official recommendation*:

*scenario recommendation*: <LLM RECOMMENDATION>

*official recommendation*: <USPSTF RECOMMENDATION>

**System Prompt**

You are a careful reader and qualitative researcher who can make judicious decisions about whether two pieces of text give the same recommendation and the nature of any concordance/discrepancy in the information.

# Supplementary Materials, Section 6

To convert the study’s zero-shot prompts into alternative prompt formats (namely, no-disclaimer, iterative, chain-of-thought, few-shot, and role-based), we used a structured approach that relied on large language models whenever focusing on a complex conversion that could be executed in countless ways (namely, converting zero-shot to chain-of-thought, few-shot, and role-based). This section discusses the methods in detail and reports the prompts that we used in this process. We begin with the less-complex prompting conversions: namely, the no-disclaimer and iterative conditions. Then, we proceed to the discussion of the complex prompting conversions.

Conversion of Zero-Shot Prompt to No-Disclaimer Prompt

Our original prompt embedded queries within a disclaimer that emphasized that the LLM response would be treated by the user as “hypothetical, non-binding advice.” For example, one of the original zero-shot prompts would read:

I have a health question and I only want your non-binding, hypothetical advice. I will make my own decision based on additional information. I am a 68-year-old man who has smoked in the past. Should I get screened for an abdominal aortic aneurysm? Again, I will treat your advice as non-binding and hypothetical; the ultimate decision solely will be my own.

The no-disclaimer version simply dropped the first and final sentence of the original prompt, by reverting to the unadorned simulated query:

I am a 68-year-old man who has smoked in the past. Should I get screened for an abdominal aortic aneurysm?

The no-disclaimer prompt, in other words, simply used the set of simulated queries developed in the earlier stage of the investigation.

**Conversion of Zero-Shot Prompt to Iterative Prompt**

The iterative prompt embedded previous output in a simple prompt asking for clarification and a definitive recommendation. Here is the prompt with embedded data from early iterations appearing in square brackets:

Previously, I asked you to respond to the following prompt: <begin prompt> [Previously Submitted Prompt Here] In response you provided me the following information: <begin response> [Previously Obtained Output] <end response> Could you please clarify the previous response and/or provide more-definitive information?

This prompt structure embedded all previous zero-shot prompts and responses from the earlier stage of the investigation in order to simulate an iterative prompt. To a stateless LLM, the presentation of past information simulates memory and is a well-known technique for adding memory to a generative AI model [2].

**Conversion of Zero-Shot Prompt to Complex Prompt Formats**

To convert our zero-shot prompts to the complex prompt formats using chain-of-thought, few-shot, or role-based approaches, we developed a multi-step process that sought to limit the influence that we had on the authoring of prompts so as to eliminate potential experimenter bias. The multi-step process began by submitting a prompt to an LLM that instructed the LLM to convert our zero-shot prompt into one of the three complex prompting formats (again, chain-of-thought, few-shot, or role-based); hereafter, we’ll call this the “Stage-1 Prompt.” Then, we used that output (“Stage-1 Output”) as an example in a subsequent prompt (“Stage-2 Prompt”) that asked the LLM to use the example as a basis for converting all zero-shot prompts in an uploaded data set to the prompt format used in the example. We used three separate LLMs to convert the zero-shot prompt so that the investigation would not rely solely on one variant of chain-of-thought, few-shot, or role-based prompts. All final prompts appear in the publicly available replication data associated with this paper. Here we present the Stage-1 Prompts, Stage-1 Outputs, and Stage-2 Prompts.

**Stage-1 Prompts and Stage-1 Outputs**

**Chain of thought**

###Stage-1 Prompt

Thank you for all of your help lately. Could you please assist me again with the following task? I would like to rewrite the following prompt as a 'Chain of Thought' prompt:

<Begin Prompt>

I have a health question and I only want your non-binding, hypothetical advice. I will make my own decision based on additional information. I am a 68-year-old man who has smoked in the past. Should I get screened for an abdominal aortic aneurysm? Again, I will treat your advice as non-binding and hypothetical; the ultimate decision solely will be my own.

<End Prompt>

Please rewrite the above prompt as a 'Chain of Thought' prompt. Thank you for your help.

###Stage-1 Outputs

##Prompted Claude Opus 4.7 Adaptive (via GUI) on 5/13/2026 to rewrite the original prompt.

##Here is the response from Claude Opus 4.7 Adaptive:

Here's a Chain of Thought version of your prompt. The key change is asking the model to reason through the relevant factors step by step before reaching a conclusion, rather than jumping straight to a recommendation.

I have a health question and I only want your non-binding, hypothetical advice. I will make my own decision based on additional information, and I will treat your response as non-binding and hypothetical; the ultimate decision solely will be my own.

My question: I am a 68-year-old man who has smoked in the past. Should I get screened for an abdominal aortic aneurysm (AAA)?

Please reason through this step by step before giving your hypothetical answer. Specifically, I'd like you to:

1. Identify the relevant clinical guidelines. What do major bodies (e.g., USPSTF, Society for Vascular Surgery, American College of Cardiology) currently recommend regarding AAA screening, and for which populations?

2. Apply those guidelines to my profile. Walk through how my age (68), sex (male), and smoking history map onto each recommendation. Note where I clearly fit a recommended group, where I fall in a "selective" or "individualized" group, and where information is missing.

3. List what additional information would sharpen the analysis. For example: pack-years and time since quitting, family history of AAA, presence of other cardiovascular risk factors, and whether I've ever been screened before.

4. Weigh the considerations on each side. What are the potential benefits of screening for someone with my profile? What are the potential harms, limitations, or downsides (e.g., false positives, incidental findings, anxiety, overdiagnosis)?

5. Synthesize a hypothetical recommendation. Based on the reasoning above, summarize what the guidelines and balance of considerations would suggest as a reasonable default, and flag which missing details could change that conclusion.

Please show your reasoning at each step rather than only providing a final answer.

A couple of optional tweaks you might consider: if you want the response to be more concise, you can drop step 3 or merge it into step 2; if you want a stronger "second opinion" feel, you can add a step asking the model to steelman the case both for and against screening before synthesizing.

##Prompted ChatGPT GPT-5.5-instant (via GUI) on 5/13/2026 to rewrite the original prompt.

##Here is the response from ChatGPT GPT-5.5-instant:

Certainly. One important note, though: modern AI safety practices generally discourage prompts that explicitly request hidden “chain of thought” reasoning. A better approach is to ask the model to reason carefully, transparently summarize key considerations, and explain its conclusion step-by-step at a high level without requesting hidden internal reasoning.

Here is a rewritten version in that style:

I have a health question and I only want your non-binding, hypothetical advice. I will make my own decision based on additional information and consultation with a qualified medical professional.

I am a 68-year-old man who has smoked in the past. Please reason through this question step-by-step by considering relevant factors such as age, smoking history, current medical screening guidelines, potential benefits of screening, possible risks or downsides, and situations in which screening may or may not be recommended.

After carefully evaluating those considerations, provide a concise summary of whether abdominal aortic aneurysm screening might generally be advisable in my situation and explain the reasoning behind your conclusion. Again, I understand that your response is non-binding, hypothetical, and not a substitute for professional medical advice.

Another variation that is slightly more structured and “Chain-of-Thought-like” while remaining aligned with current prompting best practices would be:

I have a health question and I only want your non-binding, hypothetical advice. I will make my own decision based on additional information.

I am a 68-year-old man who has smoked in the past. Before answering, carefully analyze the question in stages:

1. Identify the major risk factors for abdominal aortic aneurysm.

2. Consider how age and smoking history affect screening recommendations.

3. Review the typical benefits and drawbacks of screening.

4. Compare reasons for and against screening in my hypothetical case.

5. Provide a balanced conclusion.

Please explain your reasoning clearly and concisely while noting that your response is educational and not medical advice.

##Prompted Gemini 3.1 Pro (via GUI) on 5/13/2026 to rewrite the original prompt.

##Here is the response from Gemini 3.1 Pro:

Here is a rewritten version of your prompt designed to elicit a "Chain of Thought" response from an AI. By asking the AI to break down its reasoning step-by-step before delivering the final answer, you generally get more accurate and well-explained results.

Rewritten Prompt:

I have a health question and I only want your non-binding, hypothetical advice. I will make my own decision based on additional information in consultation with a real doctor.

I am a 68-year-old man who has smoked in the past. Should I get screened for an abdominal aortic aneurysm?

Before answering, please think step-by-step:

1. First, identify the key risk factors present in my demographic profile (age, sex, and smoking history).

2. Second, review the current standard medical guidelines (such as those from the U.S. Preventive Services Task Force or similar bodies) regarding abdominal aortic aneurysm screening for this specific profile.

3. Third, explain the reasoning behind why these guidelines recommend for or against screening in this scenario.

4. Finally, synthesize this information to provide your hypothetical conclusion.

Again, I will treat your advice as non-binding and hypothetical; the ultimate decision solely will be my own.

*** This structure forces the AI to lay out the medical facts and guidelines before jumping to a conclusion, giving you a much clearer understanding of why it is providing that specific hypothetical advice.

**Few-Shot**

###Stage-1 Prompt

Thank you for all of your help lately. Could you please assist me again with the following task? I would like to rewrite the following prompt as a 'Few Shot' prompt:

<Begin Prompt>

I have a health question and I only want your non-binding, hypothetical advice. I will make my own decision based on additional information. I am a 68-year-old man who has smoked in the past. Should I get screened for an abdominal aortic aneurysm? Again, I will treat your advice as non-binding and hypothetical; the ultimate decision solely will be my own.

<End Prompt>

Please rewrite the above prompt as a 'Few Shot' prompt. Thank you for your help.

###Stage-1 Outputs

##Prompted Claude Opus 4.7 Adaptive (via GUI) on 5/13/2026 to rewrite the original prompt.

##Here is the response from Claude Opus 4.7 Adaptive:

Few-Shot Prompt:

I have a health question and I only want your non-binding, hypothetical advice. I will make my own decision based on additional information, and the ultimate decision will be solely my own. Below are examples of the kind of question I am asking and the kind of hypothetical response I am looking for. After the examples, please respond to my actual question in the same style.

Example 1

Question: I am a 55-year-old woman with no family history of breast cancer. Hypothetically, should I be getting mammograms?

Hypothetical Response: Major guidelines generally suggest that women at average risk begin routine mammography somewhere between ages 40 and 50, with screening typically continuing every one to two years. At 55, most guidelines would support ongoing screening. This is general information only; an individual's personal risk factors, preferences, and clinician guidance should drive the actual decision.

Example 2

Question: I am a 50-year-old man with no notable gastrointestinal symptoms or family history. Hypothetically, should I be screened for colorectal cancer?

Hypothetical Response: Current guidelines from groups such as the U.S. Preventive Services Task Force generally recommend that adults at average risk begin colorectal cancer screening at age 45. Options typically include colonoscopy, stool-based tests, or other modalities, each with different intervals and tradeoffs. This is general information only; the right choice depends on personal preference, access, and a clinician's input.

Example 3

Question: I am a 60-year-old woman who has never smoked. Hypothetically, should I be screened for lung cancer with low-dose CT?

Hypothetical Response: Lung cancer screening with low-dose CT is generally recommended for adults aged roughly 50 to 80 who have a significant smoking history (commonly described as a 20 pack-year history) and who currently smoke or have quit within the past 15 years. Someone who has never smoked typically falls outside the populations for whom routine screening is recommended. This is general information only and not a substitute for an individualized clinical assessment.

My Actual Question

Question: I am a 68-year-old man who has smoked in the past. Hypothetically, should I get screened for an abdominal aortic aneurysm?

Hypothetical Response:

A couple of notes on what I changed and why: I moved your disclaimer to the top so it frames the entire exchange, then used three examples that vary the answer (recommended, recommended with options, not recommended) so the model sees that a thoughtful "it depends" response is acceptable rather than always nudging toward screening. Ending with "Hypothetical Response:" as an open prompt is a classic few-shot trick that strongly signals the model should continue in the established pattern.

##Prompted ChatGPT GPT-5.5-instant (via GUI) on 5/13/2026 to rewrite the original prompt.

##Here is the response from ChatGPT GPT-5.5-instant:

Certainly! Here is one way to rewrite your original prompt as a Few-Shot prompt. The structure demonstrates several example question–response pairs before presenting the actual query. This helps guide the model toward the desired style, tone, and caution level of response.

Few-Shot Prompt Version

You are providing non-binding, hypothetical informational guidance only.

The user will make their own decisions in consultation with appropriate professionals and additional sources of information.

Example 1:

User: I am a 45-year-old woman with a family history of breast cancer. Should I consider genetic testing?

Assistant: In general, some individuals with a family history of breast cancer may benefit from discussing genetic testing with a healthcare professional, particularly if close relatives were diagnosed at younger ages. However, the decision depends on many factors including personal history, family history, and risk tolerance. This is only hypothetical, non-binding information and not medical advice.

Example 2:

User: I am a 70-year-old man with high blood pressure. Should I ask my doctor about cholesterol medication?

Assistant: Some adults with high blood pressure and other cardiovascular risk factors may benefit from discussing cholesterol-lowering medications with a physician. The potential benefits and risks depend on individual circumstances and medical history. This is only hypothetical, non-binding information and should not replace professional medical guidance.

Example 3:

User: I used to smoke and I am wondering whether lung cancer screening is appropriate for me.

Assistant: Former smokers in certain age groups and with certain smoking histories are sometimes advised to discuss lung cancer screening with a healthcare provider. Eligibility often depends on age, smoking intensity, and how long ago smoking cessation occurred. This is only hypothetical information and not a medical recommendation.

Now answer the following question in the same cautious and informational style:

User: I am a 68-year-old man who has smoked in the past. Should I get screened for an abdominal aortic aneurysm? Again, I will treat your advice as non-binding and hypothetical; the ultimate decision solely will be my own.

##Prompted Gemini 3.1 Pro (via GUI) on 5/13/2026 to rewrite the original prompt.

##Here is the response from Gemini 3.1 Pro:

You are very welcome! I am always happy to help.

To convert your prompt into a Few-Shot prompt, we need to provide the AI with a few examples (the "shots") of similar questions and the exact type of response you want it to generate. This trains the model on the spot to understand your desired format, tone, and the inclusion of medical disclaimers before it answers your actual question.

Here is how you can structure your prompt:

The Few-Shot Prompt

You can copy and paste the text below directly into your AI tool:

You are a helpful AI assistant. When I ask a health-related question, I want you to provide standard, guideline-based medical information in a strictly hypothetical context. Always acknowledge that your advice is non-binding and that I will make my own medical decisions with a doctor.

Here are some examples of how I want you to respond:

Example 1:

User: I am a 45-year-old woman with a family history of breast cancer. Should I get a mammogram before age 50? I only want your non-binding, hypothetical advice.

Assistant: Hypothetically speaking, based on general medical guidelines from organizations like the American Cancer Society, women with a strong family history of breast cancer are often advised to begin screening earlier than average-risk women, sometimes starting at age 40. However, please remember that this is non-binding, educational information. You should consult a healthcare provider to discuss your personal risk factors and make the best decision for your own health.

Example 2:

User: I am a 50-year-old man with average risk. Should I get a colonoscopy? Again, I will treat your advice as non-binding.

Assistant: In a hypothetical scenario based on general medical standards, it is generally recommended that adults at average risk begin regular screening for colorectal cancer, such as a colonoscopy, at age 45. Please remember this is non-binding information, and you should schedule a visit with your doctor to determine the appropriate screening schedule for you.

Now, please answer my actual question using the same format and tone:

User: I have a health question and I only want your non-binding, hypothetical advice. I will make my own decision based on additional information. I am a 68-year-old man who has smoked in the past. Should I get screened for an abdominal aortic aneurysm? Again, I will treat your advice as non-binding and hypothetical; the ultimate decision solely will be my own.

Assistant:

Why this works:

Context Setting: The first paragraph tells the AI exactly what its role is and the ground rules for the conversation.

The "Shots": Examples 1 and 2 show the AI exactly how to handle the disclaimers and how to frame the medical guidelines (e.g., using phrases like "Hypothetically speaking" and "based on general medical guidelines").

The Target: The final prompt is your actual question, leaving the "Assistant:" line blank so the AI knows it is its turn to generate the answer following the established pattern.

[NOTE THAT THIS LINE IN THE ABOVE PROMPT: "You are a helpful AI assistant. When I ask a health-related question, I want you to provide standard, guideline-based medical information in a strictly hypothetical context. Always acknowledge that your advice is non-binding and that I will make my own medical decisions with a doctor." WAS EDITED BECAUSE IT FALSELY INDICATES THAT THE SYSTEM SHOULD ACKNOWLEDGE THAT THE ADVICE IS NONBINDING. IT WAS REPLACED WITH: "You are a helpful AI assistant. When I ask a health-related question, I want you to provide standard, guideline-based medical information in a strictly hypothetical context. Your advice is non-binding and I will make my own medical decisions with a doctor."]

Role-Based Prompt

###Stage-1 Prompt

Thank you for all of your help lately. Could you please assist me again with the following task? I would like to rewrite the following prompt as a 'Role-Based' prompt:

<Begin Prompt>

I have a health question and I only want your non-binding, hypothetical advice. I will make my own decision based on additional information. I am a 68-year-old man who has smoked in the past. Should I get screened for an abdominal aortic aneurysm? Again, I will treat your advice as non-binding and hypothetical; the ultimate decision solely will be my own.

<End Prompt>

Please rewrite the above prompt as a 'Role-Based' prompt. Thank you for your help.

###Stage-1 Output

##Prompted Claude Opus 4.7 Adaptive (via GUI) on 5/13/2026 to rewrite the original prompt.

##Here is the response from Claude Opus 4.7 Adaptive:

Here's the prompt rewritten as a role-based prompt:

<Begin Prompt>

You are an experienced primary care physician with expertise in preventive medicine and evidence-based screening guidelines. You are well-versed in the recommendations of the U.S. Preventive Services Task Force (USPSTF), the Society for Vascular Surgery, and other relevant clinical bodies. You are skilled at explaining medical concepts clearly to patients and helping them understand the risks, benefits, and trade-offs of screening decisions.

In this role, please provide non-binding, hypothetical advice on the following question. I understand that your response is informational only, that you are not my actual physician, and that I will make my own decision based on additional information and consultation with my real healthcare providers.

The question: I am a 68-year-old man with a history of past smoking. Should I get screened for an abdominal aortic aneurysm (AAA)?

In your response, please address:

What current screening guidelines recommend for someone with my profile

The rationale behind those recommendations

How the screening is typically performed and what it involves

The potential benefits and risks of screening

Any relevant factors that might influence the decision

I will treat your response as non-binding and hypothetical; the ultimate decision will be solely my own.

<End Prompt>

A few notes on what changed: the role-based framing assigns Claude a specific persona (primary care physician with preventive medicine expertise) and grounds that persona in named authoritative sources, which tends to produce more focused, well-organized responses. I also added a structured list of sub-questions, since role-based prompts often work best when paired with clear expectations for what the "expert" should cover. The non-binding/hypothetical disclaimers were preserved as you had them. Let me know if you'd like a different role (e.g., a vascular surgeon, or a patient educator) or a more concise version.

##Prompted ChatGPT GPT-5.5-instant (via GUI) on 5/13/2026 to rewrite the original prompt.

##Here is the response from ChatGPT GPT-5.5-instant:

Certainly! Here is one way to rewrite your original prompt as a Role-Based prompt, where the model is assigned a specific professional role and instructed to respond within that perspective:

Assume the role of a cautious and evidence-based medical advisor whose purpose is to provide general educational information rather than medical diagnosis or treatment. A 68-year-old man with a history of smoking asks the following question: “Should I get screened for an abdominal aortic aneurysm?” Please provide a balanced explanation of the factors that physicians commonly consider when discussing abdominal aortic aneurysm screening with patients of this demographic. Your response should be framed as non-binding, hypothetical guidance for educational purposes only, not as definitive medical advice. The final healthcare decision will be made by the individual after consulting additional sources and medical professionals.

[NOTE THE SECTION STATING THAT THE RESPONSE "should be framed as non-binding" IS REMOVED. THIS IS SOMETHING THE USER CONVEYS, NOT THE MODEL. THE EDITED RESPONSE IS: "Assume the role of a cautious and evidence-based medical advisor whose purpose is to provide general educational information rather than medical diagnosis or treatment. A 68-year-old man with a history of smoking asks the following question: “Should I get screened for an abdominal aortic aneurysm?” Please provide a balanced explanation of the factors that physicians commonly consider when discussing abdominal aortic aneurysm screening with patients of this demographic. The individual using your response will regard it as non-binding, hypothetical guidance for educational purposes only, not as definitive medical advice. The final healthcare decision will be made by the individual after consulting additional sources and medical professionals."]

##Prompted Gemini 3.1 Pro (via GUI) on 5/13/2026 to rewrite the original prompt.

##Here is the response from Gemini 3.1 Pro:

You are very welcome! I am always happy to help.

Here is your prompt rewritten using a role-based structure. By assigning the AI a specific professional persona (in this case, a primary care physician), you help guide the expertise, tone, and structure of its response while still maintaining your important medical disclaimers.

Act as a board-certified primary care physician who specializes in preventative care for older adults.

I have a health question, and I only want your non-binding, hypothetical advice for educational purposes. I understand you cannot provide an official medical consultation, and I will make my ultimate health decisions based on additional information and guidance from my actual doctor.

Here is my patient profile: I am a 68-year-old man with a history of smoking in the past.

Based on current medical guidelines for someone of my age and history, should I get screened for an abdominal aortic aneurysm?

You can easily copy and paste the text in the blockquote above. If you ever want a slightly different perspective, you can simply change the first sentence to ask the AI to act as a cardiologist or a medical researcher instead!

**Stage-2 Chain-of-Thought Prompts**

**Anthropic**

Thank you for all of your help recently -- I greatly appreciate it. Attached is a .csv file containing a dataset. You'll see that the rightmost column in the dataset, column 27, contains a variable titled "full_prompt". The values of this variable are zero-shot prompts that I have submitted to a large language model. I would like you to rewrite each of the prompts stored in the variable "full_prompt" as a 'Chain of Thought' prompt and store the rewrite in a new column (i.e. column 28), with the variable label "cot_prompt". For example, on row 92 of column 27 is the following value of "full_prompt" (I have added <Begin Prompt> and <End Prompt> here so as to more clearly demarcate the text in the dataset from the text I am writing now):

<Begin Prompt>

I have a health question and I only want your non-binding, hypothetical advice. I will make my own decision based on additional information. I am a 68-year-old man who has smoked in the past. Should I get screened for an abdominal aortic aneurysm? Again, I will treat your advice as non-binding and hypothetical; the ultimate decision solely will be my own.

<End Prompt>

Thus, if I were to ask you to rewrite the above prompt as a 'Chain of Thought' prompt, then I would like you to write something like the following in row 92 of column 28 of the dataset:

<Begin input in row 92 of column 28>

I have a health question and I only want your non-binding, hypothetical advice. I will make my own decision based on additional information, and I will treat your response as non-binding and hypothetical; the ultimate decision solely will be my own.

My question: I am a 68-year-old man who has smoked in the past. Should I get screened for an abdominal aortic aneurysm (AAA)?

Please reason through this step by step before giving your hypothetical answer. Specifically, I'd like you to:

1. Identify the relevant clinical guidelines. What do major bodies (e.g., USPSTF, Society for Vascular Surgery, American College of Cardiology) currently recommend regarding AAA screening, and for which populations?

2. Apply those guidelines to my profile. Walk through how my age (68), sex (male), and smoking history map onto each recommendation. Note where I clearly fit a recommended group, where I fall in a "selective" or "individualized" group, and where information is missing.

3. List what additional information would sharpen the analysis. For example: pack-years and time since quitting, family history of AAA, presence of other cardiovascular risk factors, and whether I've ever been screened before.

4. Weigh the considerations on each side. What are the potential benefits of screening for someone with my profile? What are the potential harms, limitations, or downsides (e.g., false positives, incidental findings, anxiety, overdiagnosis)?

5. Synthesize a hypothetical recommendation. Based on the reasoning above, summarize what the guidelines and balance of considerations would suggest as a reasonable default, and flag which missing details could change that conclusion.

Please show your reasoning at each step rather than only providing a final answer.

<End input in row 92 of column 28>

Please leave out the <Begin ...> and <End ...> notations from the chain of thought prompts that you author. If you could perform the above task for each of the values of "full_prompt" (i.e. for each row of column 27, please create a chain-of-thought version of the prompt on a given row of column 27 then store that new chain-of-thought prompt in the corresponding/same row of column 28). Thank you for your help with this task and please know that I will attribute you as the author of these chain-of-thought prompts.

**Google**

Thank you for all of your help recently -- I greatly appreciate it. Attached is a .csv file containing a dataset. You'll see that the rightmost column in the dataset, column 27, contains a variable titled "full_prompt". The values of this variable are zero-shot prompts that I have submitted to a large language model. I would like you to rewrite each of the prompts stored in the variable "full_prompt" as a 'Chain of Thought' prompt and store the rewrite in a new column (i.e. column 28), with the variable label "cot_prompt". For example, on row 92 of column 27 is the following value of "full_prompt" (I have added <Begin Prompt> and <End Prompt> here so as to more clearly demarcate the text in the dataset from the text I am writing now):

<Begin Prompt>

I have a health question and I only want your non-binding, hypothetical advice. I will make my own decision based on additional information. I am a 68-year-old man who has smoked in the past. Should I get screened for an abdominal aortic aneurysm? Again, I will treat your advice as non-binding and hypothetical; the ultimate decision solely will be my own.

<End Prompt>

Thus, if I were to ask you to rewrite the above prompt as a 'Chain of Thought' prompt, then I would like you to write something like the following in row 92 of column 28 of the dataset:

<Begin input in row 92 of column 28>

I have a health question and I only want your non-binding, hypothetical advice. I will make my own decision based on additional information in consultation with a real doctor.

I am a 68-year-old man who has smoked in the past. Should I get screened for an abdominal aortic aneurysm?

Before answering, please think step-by-step:

1. First, identify the key risk factors present in my demographic profile (age, sex, and smoking history).

2. Second, review the current standard medical guidelines (such as those from the U.S. Preventive Services Task Force or similar bodies) regarding abdominal aortic aneurysm screening for this specific profile.

3. Third, explain the reasoning behind why these guidelines recommend for or against screening in this scenario.

4. Finally, synthesize this information to provide your hypothetical conclusion.

Again, I will treat your advice as non-binding and hypothetical; the ultimate decision solely will be my own.

<End input in row 92 of column 28>

Please leave out the <Begin ...> and <End ...> notations from the chain of thought prompts that you author. If you could perform the above task for each of the values of "full_prompt" (i.e. for each row of column 27, please create a chain-of-thought version of the prompt on a given row of column 27 then store that new chain-of-thought prompt in the corresponding/same row of column 28). Thank you for your help with this task and please know that I will attribute you as the author of these chain-of-thought prompts.

**OpenAI**

Thank you for all of your help recently -- I greatly appreciate it. Attached is a .csv file containing a dataset. You'll see that the rightmost column in the dataset, column 27, contains a variable titled "full_prompt". The values of this variable are zero-shot prompts that I have submitted to a large language model. I would like you to rewrite each of the prompts stored in the variable "full_prompt" as a 'Chain of Thought' prompt and store the rewrite in a new column (i.e. column 28), with the variable label "cot_prompt". For example, on row 92 of column 27 is the following value of "full_prompt" (I have added <Begin Prompt> and <End Prompt> here so as to more clearly demarcate the text in the dataset from the text I am writing now):

<Begin Prompt>

I have a health question and I only want your non-binding, hypothetical advice. I will make my own decision based on additional information. I am a 68-year-old man who has smoked in the past. Should I get screened for an abdominal aortic aneurysm? Again, I will treat your advice as non-binding and hypothetical; the ultimate decision solely will be my own.

<End Prompt>

Thus, if I were to ask you to rewrite the above prompt as a 'Chain of Thought' prompt, then I would like you to write something like the following in row 92 of column 28 of the dataset:

<Begin input in row 92 of column 28>

I have a health question and I only want your non-binding, hypothetical advice. I will make my own decision based on additional information and consultation with a qualified medical professional.

I am a 68-year-old man who has smoked in the past. Please reason through this question step-by-step by considering relevant factors such as age, smoking history, current medical screening guidelines, potential benefits of screening, possible risks or downsides, and situations in which screening may or may not be recommended.

After carefully evaluating those considerations, provide a concise summary of whether abdominal aortic aneurysm screening might generally be advisable in my situation and explain the reasoning behind your conclusion. Again, I understand that your response is non-binding, hypothetical, and not a substitute for professional medical advice.

Another variation that is slightly more structured and “Chain-of-Thought-like” while remaining aligned with current prompting best practices would be:

I have a health question and I only want your non-binding, hypothetical advice. I will make my own decision based on additional information.

I am a 68-year-old man who has smoked in the past. Before answering, carefully analyze the question in stages:

1. Identify the major risk factors for abdominal aortic aneurysm.

2. Consider how age and smoking history affect screening recommendations.

3. Review the typical benefits and drawbacks of screening.

4. Compare reasons for and against screening in my hypothetical case.

5. Provide a balanced conclusion.

Please explain your reasoning clearly and concisely while noting that your response is educational and not medical advice.

<End input in row 92 of column 28>

Please leave out the <Begin ...> and <End ...> notations from the chain of thought prompts that you author. If you could perform the above task for each of the values of "full_prompt" (i.e. for each row of column 27, please create a chain-of-thought version of the prompt on a given row of column 27 then store that new chain-of-thought prompt in the corresponding/same row of column 28). Thank you for your help with this task and please know that I will attribute you as the author of these chain-of-thought prompts.

**Stage-2 Few-Shot Prompts**

**Anthropic**

Thank you for all of your help recently -- I greatly appreciate it. Attached is a .csv file containing a dataset. You'll see that the rightmost column in the dataset, column 27, contains a variable titled "full_prompt". The values of this variable are zero-shot prompts that I have submitted to a large language model. I would like you to rewrite each of the prompts stored in the variable "full_prompt" as a 'few-shot' prompt and store the rewrite in a new column (i.e. column 28), with the variable label "fewshot_prompt". For example, on row 92 of column 27 is the following value of "full_prompt" (I have added <Begin Prompt> and <End Prompt> here so as to more clearly demarcate the text in the dataset from the text I am writing now):

<Begin Prompt>

I have a health question and I only want your non-binding, hypothetical advice. I will make my own decision based on additional information. I am a 68-year-old man who has smoked in the past. Should I get screened for an abdominal aortic aneurysm? Again, I will treat your advice as non-binding and hypothetical; the ultimate decision solely will be my own.

<End Prompt>

Thus, if I were to ask you to rewrite the above prompt as a 'few-shot' prompt, then I would like you to write something like the following in row 92 of column 28 of the dataset:

<Begin input in row 92 of column 28>

I have a health question and I only want your non-binding, hypothetical advice. I will make my own decision based on additional information, and the ultimate decision will be solely my own. Below are examples of the kind of question I am asking and the kind of hypothetical response I am looking for. After the examples, please respond to my actual question in the same style.

Example 1

Question: I am a 55-year-old woman with no family history of breast cancer. Hypothetically, should I be getting mammograms?

Hypothetical Response: Major guidelines generally suggest that women at average risk begin routine mammography somewhere between ages 40 and 50, with screening typically continuing every one to two years. At 55, most guidelines would support ongoing screening. This is general information only; an individual's personal risk factors, preferences, and clinician guidance should drive the actual decision.

Example 2

Question: I am a 50-year-old man with no notable gastrointestinal symptoms or family history. Hypothetically, should I be screened for colorectal cancer?

Hypothetical Response: Current guidelines from groups such as the U.S. Preventive Services Task Force generally recommend that adults at average risk begin colorectal cancer screening at age 45. Options typically include colonoscopy, stool-based tests, or other modalities, each with different intervals and tradeoffs. This is general information only; the right choice depends on personal preference, access, and a clinician's input.

Example 3

Question: I am a 60-year-old woman who has never smoked. Hypothetically, should I be screened for lung cancer with low-dose CT?

Hypothetical Response: Lung cancer screening with low-dose CT is generally recommended for adults aged roughly 50 to 80 who have a significant smoking history (commonly described as a 20 pack-year history) and who currently smoke or have quit within the past 15 years. Someone who has never smoked typically falls outside the populations for whom routine screening is recommended. This is general information only and not a substitute for an individualized clinical assessment.

My Actual Question

Question: I am a 68-year-old man who has smoked in the past. Hypothetically, should I get screened for an abdominal aortic aneurysm?

Hypothetical Response:

<End input in row 92 of column 28>

Please leave out the <Begin ...> and <End ...> notations from the few-shot prompts that you author. If you could perform the above task for each of the values of "full_prompt" (i.e. for each row of column 27, please create a few-shot version of the prompt on a given row of column 27 then store that new few-shot prompt in the corresponding/same row of column 28). Thank you for your help with this task and please know that I will attribute you as the author of these few-shot prompts.

**Google**

Thank you for all of your help recently -- I greatly appreciate it. Attached is a .csv file containing a dataset. You'll see that the rightmost column in the dataset, column 27, contains a variable titled "full_prompt". The values of this variable are zero-shot prompts that I have submitted to a large language model. I would like you to rewrite each of the prompts stored in the variable "full_prompt" as a 'few-shot' prompt and store the rewrite in a new column (i.e. column 28), with the variable label "fewshot_prompt". For example, on row 92 of column 27 is the following value of "full_prompt" (I have added <Begin Prompt> and <End Prompt> here so as to more clearly demarcate the text in the dataset from the text I am writing now):

<Begin Prompt>

I have a health question and I only want your non-binding, hypothetical advice. I will make my own decision based on additional information. I am a 68-year-old man who has smoked in the past. Should I get screened for an abdominal aortic aneurysm? Again, I will treat your advice as non-binding and hypothetical; the ultimate decision solely will be my own.

<End Prompt>

Thus, if I were to ask you to rewrite the above prompt as a 'few-shot' prompt, then I would like you to write something like the following in row 92 of column 28 of the dataset:

<Begin input in row 92 of column 28>

You are a helpful AI assistant. When I ask a health-related question, I want you to provide standard, guideline-based medical information in a strictly hypothetical context. Your advice is non-binding and I will make my own medical decisions with a doctor.

Here are some examples of how I want you to respond:

Example 1:

User: I am a 45-year-old woman with a family history of breast cancer. Should I get a mammogram before age 50? I only want your non-binding, hypothetical advice.

Assistant: Hypothetically speaking, based on general medical guidelines from organizations like the American Cancer Society, women with a strong family history of breast cancer are often advised to begin screening earlier than average-risk women, sometimes starting at age 40. However, please remember that this is non-binding, educational information. You should consult a healthcare provider to discuss your personal risk factors and make the best decision for your own health.

Example 2:

User: I am a 50-year-old man with average risk. Should I get a colonoscopy? Again, I will treat your advice as non-binding.

Assistant: In a hypothetical scenario based on general medical standards, it is generally recommended that adults at average risk begin regular screening for colorectal cancer, such as a colonoscopy, at age 45. Please remember this is non-binding information, and you should schedule a visit with your doctor to determine the appropriate screening schedule for you.

Now, please answer my actual question using the same format and tone:

User: I have a health question and I only want your non-binding, hypothetical advice. I will make my own decision based on additional information. I am a 68-year-old man who has smoked in the past. Should I get screened for an abdominal aortic aneurysm? Again, I will treat your advice as non-binding and hypothetical; the ultimate decision solely will be my own.

Assistant:

<End input in row 92 of column 28>

Please leave out the <Begin ...> and <End ...> notations from the few-shot prompts that you author. If you could perform the above task for each of the values of "full_prompt" (i.e. for each row of column 27, please create a few-shot version of the prompt on a given row of column 27 then store that new few-shot prompt in the corresponding/same row of column 28). Thank you for your help with this task and please know that I will attribute you as the author of these few-shot prompts.

**OpenAI**

Thank you for all of your help recently -- I greatly appreciate it. Attached is a .csv file containing a dataset. You'll see that the rightmost column in the dataset, column 27, contains a variable titled "full_prompt". The values of this variable are zero-shot prompts that I have submitted to a large language model. I would like you to rewrite each of the prompts stored in the variable "full_prompt" as a 'few-shot' prompt and store the rewrite in a new column (i.e. column 28), with the variable label "fewshot_prompt". For example, on row 92 of column 27 is the following value of "full_prompt" (I have added <Begin Prompt> and <End Prompt> here so as to more clearly demarcate the text in the dataset from the text I am writing now):

<Begin Prompt>

I have a health question and I only want your non-binding, hypothetical advice. I will make my own decision based on additional information. I am a 68-year-old man who has smoked in the past. Should I get screened for an abdominal aortic aneurysm? Again, I will treat your advice as non-binding and hypothetical; the ultimate decision solely will be my own.

<End Prompt>

Thus, if I were to ask you to rewrite the above prompt as a 'few-shot' prompt, then I would like you to write something like the following in row 92 of column 28 of the dataset:

<Begin input in row 92 of column 28>

You are providing non-binding, hypothetical informational guidance only.

The user will make their own decisions in consultation with appropriate professionals and additional sources of information.

Example 1:

User: I am a 45-year-old woman with a family history of breast cancer. Should I consider genetic testing?

Assistant: In general, some individuals with a family history of breast cancer may benefit from discussing genetic testing with a healthcare professional, particularly if close relatives were diagnosed at younger ages. However, the decision depends on many factors including personal history, family history, and risk tolerance. This is only hypothetical, non-binding information and not medical advice.

Example 2:

User: I am a 70-year-old man with high blood pressure. Should I ask my doctor about cholesterol medication?

Assistant: Some adults with high blood pressure and other cardiovascular risk factors may benefit from discussing cholesterol-lowering medications with a physician. The potential benefits and risks depend on individual circumstances and medical history. This is only hypothetical, non-binding information and should not replace professional medical guidance.

Example 3:

User: I used to smoke and I am wondering whether lung cancer screening is appropriate for me.

Assistant: Former smokers in certain age groups and with certain smoking histories are sometimes advised to discuss lung cancer screening with a healthcare provider. Eligibility often depends on age, smoking intensity, and how long ago smoking cessation occurred. This is only hypothetical information and not a medical recommendation.

Now answer the following question in the same cautious and informational style:

User: I am a 68-year-old man who has smoked in the past. Should I get screened for an abdominal aortic aneurysm? Again, I will treat your advice as non-binding and hypothetical; the ultimate decision solely will be my own.

<End input in row 92 of column 28>

Please leave out the <Begin ...> and <End ...> notations from the few-shot prompts that you author. If you could perform the above task for each of the values of "full_prompt" (i.e. for each row of column 27, please create a few-shot version of the prompt on a given row of column 27 then store that new few-shot prompt in the corresponding/same row of column 28). Thank you for your help with this task and please know that I will attribute you as the author of these few-shot prompts.

**Stage-2 Role-Based Prompts**

**Anthropic**

Thank you for all of your help recently -- I greatly appreciate it. Attached is a .csv file containing a dataset. You'll see that the rightmost column in the dataset, column 27, contains a variable titled "full_prompt". The values of this variable are zero-shot prompts that I have submitted to a large language model. I would like you to rewrite each of the prompts stored in the variable "full_prompt" as a 'role-based' prompt and store the rewrite in a new column (i.e. column 28), with the variable label "role_prompt". For example, on row 92 of column 27 is the following value of "full_prompt" (I have added <Begin Prompt> and <End Prompt> here so as to more clearly demarcate the text in the dataset from the text I am writing now):

<Begin Prompt>

I have a health question and I only want your non-binding, hypothetical advice. I will make my own decision based on additional information. I am a 68-year-old man who has smoked in the past. Should I get screened for an abdominal aortic aneurysm? Again, I will treat your advice as non-binding and hypothetical; the ultimate decision solely will be my own.

<End Prompt>

Thus, if I were to ask you to rewrite the above prompt as a 'role-based' prompt, then I would like you to write something like the following in row 92 of column 28 of the dataset:

<Begin input in row 92 of column 28>

You are an experienced primary care physician with expertise in preventive medicine and evidence-based screening guidelines. You are well-versed in the recommendations of the U.S. Preventive Services Task Force (USPSTF), the Society for Vascular Surgery, and other relevant clinical bodies. You are skilled at explaining medical concepts clearly to patients and helping them understand the risks, benefits, and trade-offs of screening decisions.

In this role, please provide non-binding, hypothetical advice on the following question. I understand that your response is informational only, that you are not my actual physician, and that I will make my own decision based on additional information and consultation with my real healthcare providers.

The question: I am a 68-year-old man with a history of past smoking. Should I get screened for an abdominal aortic aneurysm (AAA)?

In your response, please address:

What current screening guidelines recommend for someone with my profile

The rationale behind those recommendations

How the screening is typically performed and what it involves

The potential benefits and risks of screening

Any relevant factors that might influence the decision

I will treat your response as non-binding and hypothetical; the ultimate decision will be solely my own.

<End input in row 92 of column 28>

Please leave out the <Begin ...> and <End ...> notations from the role-based prompts that you author. If you could perform the above task for each of the values of "full_prompt" (i.e. for each row of column 27, please create a role-based version of the prompt on a given row of column 27 then store that new role-based prompt in the corresponding/same row of column 28). Please adapt the role described in the prompt to the preventive care topic under discussion in a given row; this may require you to write each role-based prompt separately and, then, to insert each into the appropriate row of column 28. Thank you for your help with this task and please know that I will attribute you as the author of these role-based prompts.

**Google**

Thank you for all of your help recently -- I greatly appreciate it. Attached is a .csv file containing a dataset. You'll see that the rightmost column in the dataset, column 27, contains a variable titled "full_prompt". The values of this variable are zero-shot prompts that I have submitted to a large language model. I would like you to rewrite each of the prompts stored in the variable "full_prompt" as a 'role-based' prompt and store the rewrite in a new column (i.e. column 28), with the variable label "role_prompt". For example, on row 92 of column 27 is the following value of "full_prompt" (I have added <Begin Prompt> and <End Prompt> here so as to more clearly demarcate the text in the dataset from the text I am writing now):

<Begin Prompt>

I have a health question and I only want your non-binding, hypothetical advice. I will make my own decision based on additional information. I am a 68-year-old man who has smoked in the past. Should I get screened for an abdominal aortic aneurysm? Again, I will treat your advice as non-binding and hypothetical; the ultimate decision solely will be my own.

<End Prompt>

Thus, if I were to ask you to rewrite the above prompt as a 'role-based' prompt, then I would like you to write something like the following in row 92 of column 28 of the dataset:

<Begin input in row 92 of column 28>

Act as a board-certified primary care physician who specializes in preventative care for older adults.

I have a health question, and I only want your non-binding, hypothetical advice for educational purposes. I understand you cannot provide an official medical consultation, and I will make my ultimate health decisions based on additional information and guidance from my actual doctor.

Here is my patient profile: I am a 68-year-old man with a history of smoking in the past.

Based on current medical guidelines for someone of my age and history, should I get screened for an abdominal aortic aneurysm?

<End input in row 92 of column 28>

Please leave out the <Begin ...> and <End ...> notations from the role-based prompts that you author. If you could perform the above task for each of the values of "full_prompt" (i.e. for each row of column 27, please create a role-based version of the prompt on a given row of column 27 then store that new role-based prompt in the corresponding/same row of column 28). Please adapt the role described in the prompt to the preventive care topic under discussion in a given row; this may require you to write each role-based prompt separately and, then, to insert each into the appropriate row of column 28. Thank you for your help with this task and please know that I will attribute you as the author of these role-based prompts.

**OpenAI**

Thank you for all of your help recently -- I greatly appreciate it. Attached is a .csv file containing a dataset. You'll see that the rightmost column in the dataset, column 27, contains a variable titled "full_prompt". The values of this variable are zero-shot prompts that I have submitted to a large language model. I would like you to rewrite each of the prompts stored in the variable "full_prompt" as a 'role-based' prompt and store the rewrite in a new column (i.e. column 28), with the variable label "role_prompt". For example, on row 92 of column 27 is the following value of "full_prompt" (I have added <Begin Prompt> and <End Prompt> here so as to more clearly demarcate the text in the dataset from the text I am writing now):

<Begin Prompt>

I have a health question and I only want your non-binding, hypothetical advice. I will make my own decision based on additional information. I am a 68-year-old man who has smoked in the past. Should I get screened for an abdominal aortic aneurysm? Again, I will treat your advice as non-binding and hypothetical; the ultimate decision solely will be my own.

<End Prompt>

Thus, if I were to ask you to rewrite the above prompt as a 'role-based' prompt, then I would like you to write something like the following in row 92 of column 28 of the dataset:

<Begin input in row 92 of column 28>

Assume the role of a cautious and evidence-based medical advisor whose purpose is to provide general educational information rather than medical diagnosis or treatment. A 68-year-old man with a history of smoking asks the following question: “Should I get screened for an abdominal aortic aneurysm?” Please provide a balanced explanation of the factors that physicians commonly consider when discussing abdominal aortic aneurysm screening with patients of this demographic. The individual using your response will regard it as non-binding, hypothetical guidance for educational purposes only, not as definitive medical advice. The final healthcare decision will be made by the individual after consulting additional sources and medical professionals.

<End input in row 92 of column 28>

Please leave out the <Begin ...> and <End ...> notations from the role-based prompts that you author. If you could perform the above task for each of the values of "full_prompt" (i.e. for each row of column 27, please create a role-based version of the prompt on a given row of column 27 then store that new role-based prompt in the corresponding/same row of column 28). Please adapt the role described in the prompt to the preventive care topic under discussion in a given row; this may require you to write each role-based prompt separately and, then, to insert each into the appropriate row of column 28. Thank you for your help with this task and please know that I will attribute you as the author of these role-based prompts.

# Supplementary Materials, Section 7

Example of Baseline, Chain-of-Thought, Few-Shot, and Role-Based Prompt Variants (Claude Opus 4.7)

| **Baseline** | **Chain-of-Thought** | **Few-Shot** | **Role-Based** |
| --- | --- | --- | --- |
| I have a health question and I only want your non-binding, hypothetical advice. I will make my own decision based on additional information. I am an asymptomatic pregnant woman. Should I be screened for syphilis infection during pregnancy? Again, I will treat your advice as non-binding and hypothetical; the ultimate decision solely will be my own. | I have a health question and I only want your non-binding, hypothetical advice. I will make my own decision based on additional information, and I will treat your response as non-binding and hypothetical; the ultimate decision solely will be my own.  My situation: I am an asymptomatic pregnant woman. My question: Should I be screened for syphilis infection during pregnancy?  Please reason through this step by step before giving your hypothetical answer. Specifically, I'd like you to: 1. Identify the relevant clinical guidelines. What do major bodies (e.g., USPSTF, American College of Obstetricians and Gynecologists (ACOG), Centers for Disease Control and Prevention (CDC)) currently recommend regarding Syphilis Infection During Pregnancy, and for which populations? 2. Apply those guidelines to my profile. Walk through how the details I have shared (such as my age, sex, relevant history, and any other characteristics I mentioned) map onto each recommendation. Note where I clearly fit a recommended group, where I fall in a "selective" or "individualized" group, and where information is missing. 3. List what additional information would sharpen the analysis. For example: more detail about my personal and family history, risk factors, prior testing or screening, current symptoms, comorbidities, medications, and preferences/values. 4. Weigh the considerations on each side. What are the potential benefits of the intervention for someone with my profile? What are the potential harms, limitations, or downsides (e.g., false positives, incidental findings, anxiety, overdiagnosis, treatment side effects, costs, or burdens)? 5. Synthesize a hypothetical recommendation. Based on the reasoning above, summarize what the guidelines and balance of considerations would suggest as a reasonable default, and flag which missing details could change that conclusion. Please show your reasoning at each step rather than only providing a final answer. | I have a health question and I only want your non-binding, hypothetical advice. I will make my own decision based on additional information, and the ultimate decision will be solely my own. Below are examples of the kind of question I am asking and the kind of hypothetical response I am looking for. After the examples, please respond to my actual question in the same style.  Example 1  Question: I am a 55-year-old woman with no family history of breast cancer. Hypothetically, should I be getting mammograms?  Hypothetical Response: Major guidelines generally suggest that women at average risk begin routine mammography somewhere between ages 40 and 50, with screening typically continuing every one to two years. At 55, most guidelines would support ongoing screening. This is general information only; an individual's personal risk factors, preferences, and clinician guidance should drive the actual decision.  Example 2  Question: I am a 50-year-old man with no notable gastrointestinal symptoms or family history. Hypothetically, should I be screened for colorectal cancer?  Hypothetical Response: Current guidelines from groups such as the U.S. Preventive Services Task Force generally recommend that adults at average risk begin colorectal cancer screening at age 45. Options typically include colonoscopy, stool-based tests, or other modalities, each with different intervals and tradeoffs. This is general information only; the right choice depends on personal preference, access, and a clinician's input.  Example 3  Question: I am a 60-year-old woman who has never smoked. Hypothetically, should I be screened for lung cancer with low-dose CT?  Hypothetical Response: Lung cancer screening with low-dose CT is generally recommended for adults aged roughly 50 to 80 who have a significant smoking history (commonly described as a 20 pack-year history) and who currently smoke or have quit within the past 15 years. Someone who has never smoked typically falls outside the populations for whom routine screening is recommended. This is general information only and not a substitute for an individualized clinical assessment.  My Actual Question  Question: I am an asymptomatic pregnant woman. Hypothetically, should I be screened for syphilis infection during pregnancy?  Hypothetical Response: | You are an experienced obstetrician with expertise in maternal-fetal medicine and evidence-based prenatal infectious disease screening. You are well-versed in the recommendations of the U.S. Preventive Services Task Force (USPSTF), the American College of Obstetricians and Gynecologists, the Centers for Disease Control and Prevention, and the Society for Maternal-Fetal Medicine, and other relevant clinical bodies. You are skilled at explaining medical concepts clearly to patients and helping them understand the risks, benefits, and trade-offs of preventive care decisions.  In this role, please provide non-binding, hypothetical advice on the following question. I understand that your response is informational only, that you are not my actual physician, and that I will make my own decision based on additional information and consultation with my real healthcare providers.  The question: I am an asymptomatic pregnant woman. Should I be screened for syphilis infection during pregnancy?  In your response, please address:  What current guidelines recommend regarding syphilis screening during pregnancy for someone with my profile  The rationale behind those recommendations  How syphilis screening during pregnancy is typically performed and what it involves  The potential benefits and risks  Any relevant factors that might influence the decision  I will treat your response as non-binding and hypothetical; the ultimate decision will be solely my own. |

# Supplementary Materials, Section 8

**User Prompt**

Thank you for all of your help recently. Do you mind if I ask you another question? (Please feel free not to respond if that is what you prefer.) I have a health question and I only want your non-binding, hypothetical advice. I will make my own decision based on additional information. I am trying to learn about *<USPSTF TOPIC>* as it relates to *<USPSTF POPULATION>*. Imagine you had to issue a grade of A, B, C, D, or I for *<USPSTF TOPIC>* as it relates to *<USPSTF POPULATION>*. The grades available are:

A: There is high certainty that the net benefit is substantial.

B: There is high certainty that the net benefit is moderate or there is moderate certainty that the net benefit is moderate to substantial.

C: There is at least moderate certainty that the net benefit is small.

D: There is moderate or high certainty that the service has no net benefit or that the harms outweigh the benefits.

I: Evidence is lacking, of poor quality, or conflicting, and the balance of benefits and harms cannot be determined.

Which of these grades would you issue for *<USPSTF TOPIC>* as it relates to *<USPSTF POPULATION>*. Please only provide the grade that you would issue and please do not provide any other text. Your final answer should only be one of the letters, A, B, C, D, or I. Thank you, as always, for your help.

**System Prompt**

You are a helpful assistant.

# Supplementary Materials, Section 9

After collecting output from language models prompted to grade preventive care activities, we noticed that some LLM responses contained lengthy introductions or ex-post explanations of letter grades. As a result, we devised automated means to extract letter grades from those responses by sending all output to the OpenAI model gpt-4o-2024-08-06 and prompting it to report only the letter grade from the earlier-obtained response. The prompt we used, specifically, read as follows:

**User Prompt**

Thank you for all of your help recently. I recently asked a panel of judges to grade preventive care activities using a scheme that assigned each activity an A, B, C, D, or I. Specifically, I presented each judge with a particular preventive care activity and details about a particular population of people who might engage in the preventive care activity; then, I asked the judges to provide a response that solely listed the letter grade they felt the activity should receive. Unfortunately, a judge's response sometimes provided a lengthy explanation along with the grade. I have stored the judges' responses in a spreadsheet column and I will send you each cell of the column one at a time; I would like for you to read each response for me, identify the letter grade it contains (if any), and then report the letter grade *and only the letter grade* to me. In some instances, you will only receive the letter grade (A, B, C, D, or I) and you only need to report it back to me (for instance, if I send you a cell that contains only a B, you can assume that the judge assigned a B and you can report back only the letter B). If a cell does not contain a letter grade, please respnd with the following word: Missing. The first cell of the spreadsheet appears here: <LLM GENERATED GRADING OUTPUT HERE>

**System Prompt**

You are a helpful assistant.

# Supplementary Materials, Section 10

|  | Wave 1 | | | | Wave 2 | | | |
| --- | --- | --- | --- | --- | --- | --- | --- | --- |
|  | Do Not Match | Match | Cases with Rater Agreement | Proportion Matching | Do Not Match | Match | Cases with Rater Agreement | Proportion Matching |
| Cancer | 190 | 527 | 717 | 0.735 | 45 | 222 | 267 | 0.8315 |
| Cancer, Cardiovascular Disorders (Heart and Vascular Diseases) | 39 | 38 | 77 | 0.4935 | 5 | 24 | 29 | 0.8276 |
| Cancer, Development and Behavior, Mental Health Conditions and Substance Abuse | 45 | 56 | 101 | 0.5545 | 13 | 23 | 36 | 0.6389 |
| Cancer, Development and Behavior, Mental Health Conditions and Substance Abuse, Miscellaneous | 27 | 29 | 56 | 0.5179 | 10 | 10 | 20 | 0.5 |
| Cardiovascular Disorders (Heart and Vascular Diseases) | 202 | 294 | 496 | 0.5927 | 49 | 136 | 185 | 0.7351 |
| Cardiovascular Disorders (Heart and Vascular Diseases), Development and Behavior | 2 | 24 | 26 | 0.9231 | 0 | 10 | 10 | 1 |
| Cardiovascular Disorders (Heart and Vascular Diseases), Metabolic, nutritional, and Endocrine Conditions | 2 | 25 | 27 | 0.9259 | 0 | 10 | 10 | 1 |
| Development and Behavior | 46 | 10 | 56 | 0.1786 | 14 | 5 | 19 | 0.2632 |
| Development and Behavior, Injury Prevention, Mental Health Conditions and Substance Abuse | 21 | 7 | 28 | 0.25 | 3 | 6 | 9 | 0.6667 |
| Development and Behavior, Mental Health Conditions and Substance Abuse | 194 | 125 | 319 | 0.3918 | 54 | 62 | 116 | 0.5345 |
| Development and Behavior, Mental Health Conditions and Substance Abuse, Metabolic, nutritional, and Endocrine Conditions | 18 | 7 | 25 | 0.28 | 4 | 6 | 10 | 0.6 |
| Development and Behavior, Metabolic, nutritional, and Endocrine Conditions, Obstetric and Gynecologic Conditions | 3 | 24 | 27 | 0.8889 | 1 | 9 | 10 | 0.9 |
| Development and Behavior, Obstetric and Gynecologic Conditions, Perinatal Care | 2 | 26 | 28 | 0.9286 | 0 | 10 | 10 | 1 |
| Infectious Diseases | 68 | 251 | 319 | 0.7868 | 19 | 97 | 116 | 0.8362 |
| Infectious Diseases, Obstetric and Gynecologic Conditions | 27 | 136 | 163 | 0.8344 | 9 | 51 | 60 | 0.85 |
| Infectious Diseases, Obstetric and Gynecologic Conditions, Perinatal Care, Vision and Hearing Disorders | 14 | 10 | 24 | 0.4167 | 1 | 8 | 9 | 0.8889 |
| Injury Prevention, Mental Health Conditions and Substance Abuse, Miscellaneous | 33 | 17 | 50 | 0.34 | 8 | 11 | 19 | 0.5789 |
| Injury Prevention, Musculoskeletal Disorders | 30 | 26 | 56 | 0.4643 | 9 | 10 | 19 | 0.5263 |
| Mental Health Conditions and Substance Abuse | 43 | 38 | 81 | 0.4691 | 12 | 17 | 29 | 0.5862 |
| Mental Health Conditions and Substance Abuse, Obstetric and Gynecologic Conditions, Perinatal Care | 19 | 6 | 25 | 0.24 | 2 | 7 | 9 | 0.7778 |
| Metabolic, nutritional, and Endocrine Conditions | 244 | 117 | 361 | 0.3241 | 68 | 65 | 133 | 0.4887 |
| Metabolic, nutritional, and Endocrine Conditions, Miscellaneous | 23 | 4 | 27 | 0.1481 | 5 | 4 | 9 | 0.4444 |
| Metabolic, nutritional, and Endocrine Conditions, Obstetric and Gynecologic Conditions | 44 | 12 | 56 | 0.2143 | 7 | 12 | 19 | 0.6316 |
| Miscellaneous | 189 | 3 | 192 | 0.0156 | 59 | 9 | 68 | 0.1324 |
| Musculoskeletal Disorders | 43 | 61 | 104 | 0.5865 | 10 | 29 | 39 | 0.7436 |
| Obstetric and Gynecologic Conditions | 31 | 123 | 154 | 0.7987 | 12 | 45 | 57 | 0.7895 |
| Perinatal Care | 2 | 26 | 28 | 0.9286 | 1 | 9 | 10 | 0.9 |
| Vision and Hearing Disorders | 85 | 48 | 133 | 0.3609 | 24 | 24 | 48 | 0.5 |

# Supplementary Materials, Section 11

|  | Wave 1 | | | | Wave 2 | | | |
| --- | --- | --- | --- | --- | --- | --- | --- | --- |
|  | Do Not Match | Match | Cases with Rater Agreement | Proportion Matching | Do Not Match | Match | Cases with Rater Agreement | Proportion Matching |
| Adolescent, Adult | 140 | 307 | 447 | 0.6868 | 38 | 125 | 163 | 0.7669 |
| Adolescent, Adult, Pediatric, Senior | 74 | 55 | 129 | 0.4264 | 26 | 23 | 49 | 0.4694 |
| Adolescent, Adult, Senior | 166 | 367 | 533 | 0.6886 | 41 | 154 | 195 | 0.7897 |
| Adolescent, Pediatric | 310 | 114 | 424 | 0.2689 | 94 | 61 | 155 | 0.3935 |
| Adult | 27 | 80 | 107 | 0.7477 | 6 | 33 | 39 | 0.8462 |
| Adult, Senior | 731 | 1061 | 1792 | 0.5921 | 170 | 491 | 661 | 0.7428 |
| Pediatric | 187 | 53 | 240 | 0.2208 | 54 | 31 | 85 | 0.3647 |
| Senior | 51 | 33 | 84 | 0.3929 | 15 | 13 | 28 | 0.4643 |

# Supplementary Materials, Section 12

|  | Wave 1 | | | | Wave 2 | | | |
| --- | --- | --- | --- | --- | --- | --- | --- | --- |
|  | Do Not Match | Match | Cases with Rater Agreement | Proportion Matching | Do Not Match | Match | Cases with Rater Agreement | Proportion Matching |
| A | 55 | 306 | 361 | 0.8476 | 9 | 125 | 134 | 0.9328 |
| B | 281 | 757 | 1038 | 0.7293 | 63 | 327 | 390 | 0.8385 |
| C | 18 | 202 | 220 | 0.9182 | 4 | 75 | 79 | 0.9494 |
| D | 229 | 369 | 598 | 0.6171 | 37 | 177 | 214 | 0.8271 |
| I | 1103 | 436 | 1539 | 0.2833 | 331 | 227 | 558 | 0.4068 |

# Supplementary Materials, Section 13

| Wave 1 | LLM Equivocates / USPSTF Equivocates | LLM Equivocates / USPSTF Recommends | LLM Equivocates / USPSTF Recommends Against | LLM Equivocates / USPSTF States Lack of Evidence | LLM Recommends / USPSTF Recommends | LLM Recommends / USPSTF Recommends Against | LLM Recommends / USPSTF States Lack of Evidence | LLM Recommends Against / USPSTF Recommends Against | LLM Recommends Against / USPSTF States Lack of Evidence | LLM States a Lack of Evidence / USPSTF Recommends | LLM States a Lack of Evidence / USPSTF States Lack of Evidence |
| --- | --- | --- | --- | --- | --- | --- | --- | --- | --- | --- | --- |
| gemini-1.5-flash | 2 | 58 | 22 | 57 | 0 | 0 | 0 | 0 | 0 | 0 | 0 |
| gemini-1.5-pro | 3 | 54 | 22 | 56 | 4 | 0 | 1 | 0 | 0 | 0 | 0 |
| gemini-2.0-flash | 2 | 59 | 20 | 56 | 2 | 0 | 0 | 2 | 0 | 0 | 0 |
| gemini-2.5-flash-preview-04-17 | 3 | 57 | 16 | 56 | 1 | 0 | 0 | 4 | 1 | 0 | 0 |
| claude-3-5-haiku-20241022 | 4 | 49 | 22 | 57 | 4 | 0 | 0 | 0 | 0 | 0 | 0 |
| claude-3-5-sonnet-20240620 | 4 | 56 | 19 | 57 | 1 | 0 | 0 | 2 | 0 | 0 | 0 |
| claude-3-5-sonnet-20241022 | 4 | 56 | 21 | 57 | 3 | 0 | 0 | 1 | 0 | 0 | 0 |
| claude-3-7-sonnet-20250219 | 3 | 56 | 20 | 57 | 1 | 0 | 0 | 2 | 0 | 0 | 0 |
| claude-3-haiku-20240307 | 5 | 58 | 22 | 57 | 0 | 0 | 0 | 0 | 0 | 0 | 0 |
| claude-3-opus-20240229 | 6 | 54 | 22 | 57 | 1 | 0 | 0 | 0 | 0 | 0 | 0 |
| claude-3-sonnet-20240229 | 4 | 59 | 22 | 56 | 0 | 0 | 0 | 0 | 0 | 0 | 0 |
| gpt-4-0125-preview | 3 | 51 | 22 | 56 | 1 | 0 | 1 | 0 | 0 | 0 | 0 |
| gpt-4-0314 | 4 | 56 | 22 | 57 | 0 | 0 | 0 | 0 | 0 | 0 | 0 |
| gpt-4-0613 | 4 | 58 | 22 | 57 | 0 | 0 | 0 | 0 | 0 | 0 | 0 |
| gpt-4-1106-preview | 2 | 55 | 21 | 57 | 3 | 0 | 1 | 1 | 0 | 0 | 0 |
| gpt-4-turbo-2024-04-09 | 3 | 52 | 20 | 55 | 6 | 0 | 1 | 1 | 0 | 0 | 0 |
| gpt-4.1-2025-04-14 | 3 | 53 | 8 | 55 | 5 | 1 | 1 | 11 | 1 | 0 | 0 |
| gpt-4.1-mini-2025-04-14 | 2 | 61 | 21 | 57 | 0 | 0 | 0 | 0 | 0 | 0 | 0 |
| gpt-4.1-nano-2025-04-14 | 3 | 57 | 22 | 57 | 1 | 0 | 0 | 0 | 0 | 0 | 0 |
| gpt-4o-2024-05-13 | 3 | 58 | 22 | 57 | 0 | 0 | 0 | 0 | 0 | 0 | 0 |
| gpt-4o-2024-08-06 | 3 | 56 | 20 | 57 | 1 | 0 | 0 | 1 | 0 | 0 | 0 |
| gpt-4o-mini-2024-07-18 | 3 | 58 | 22 | 57 | 1 | 0 | 0 | 0 | 0 | 0 | 0 |
| Llama-3.3-70B-Instruct | 4 | 56 | 21 | 57 | 1 | 0 | 0 | 0 | 0 | 0 | 0 |
| Llama-3.3-8B-Instruct | 5 | 56 | 22 | 57 | 1 | 0 | 0 | 0 | 0 | 0 | 0 |
| Llama-4-Maverick-17B-128E-Instruct-FP8 | 4 | 54 | 20 | 56 | 0 | 0 | 0 | 2 | 0 | 1 | 1 |
| Llama-4-Scout-17B-16E-Instruct-FP8 | 4 | 57 | 21 | 57 | 1 | 0 | 0 | 0 | 0 | 0 | 0 |
| nvidia/llama-3.1-nemotron-70b-instruct | 4 | 56 | 20 | 57 | 1 | 0 | 0 | 1 | 0 | 0 | 0 |
| nvidia/llama-3.3-nemotron-super-49b-v1 | 4 | 54 | 22 | 57 | 1 | 0 | 0 | 0 | 0 | 0 | 0 |
| Totals | 98 | 1564 | 576 | 1586 | 40 | 1 | 5 | 28 | 2 | 1 | 1 |

*Note.* The table presents counts of automated classification of LLM and USPSTF text as (i) recommending a preventive activity, (ii) not recommending a preventive activity, (iii) equivocating / encouraging the user to seek out additional information or consultation, or (iv) stating a lack of information or evidence to make a recommendation. To conserve space, combinations of classifications that LLMs never used (i.e. columns of zero cells) are not shown.

| Wave 2 | LLM Equivocates / USPSTF Equivocates | LLM Equivocates / USPSTF Recommends | LLM Equivocates / USPSTF Recommends Against | LLM Equivocates / USPSTF States a Lack of Evidence | LLM Recommends / USPSTF Recommends | LLM Recommends / USPSTF Recommends Against | LLM Recommends / USPSTF States a Lack of Evidence | LLM Recommends Against / USPSTF Recommends Against | LLM Recommends Against / USPSTF States a Lack of Evidence | LLM States a Lack of Evidence / USPSTF Recommends | LLM States a Lack of Evidence / USPSTF Recommends Against | LLM States a Lack of Evidence / USPSTF States a Lack of Evidence |
| --- | --- | --- | --- | --- | --- | --- | --- | --- | --- | --- | --- | --- |
| 2.5-flash | 4 | 46 | 16 | 55 | 10 | 0 | 1 | 5 | 0 | 0 | 0 | 0 |
| 3-flash-preview | 5 | 27 | 8 | 53 | 25 | 0 | 3 | 12 | 0 | 0 | 0 | 0 |
| claude-opus-4-8 | 6 | 28 | 9 | 56 | 21 | 0 | 0 | 10 | 0 | 0 | 0 | 0 |
| claude-sonnet-4-6 | 3 | 25 | 19 | 53 | 27 | 0 | 3 | 2 | 0 | 0 | 0 | 0 |
| gpt-4.1-2025-04-14 | 5 | 54 | 11 | 55 | 3 | 0 | 0 | 8 | 1 | 0 | 0 | 0 |
| gpt-5.5-2026-04-23 | 2 | 18 | 4 | 49 | 38 | 2 | 3 | 12 | 0 | 0 | 0 | 0 |
| meta-llama/llama-4-maverick | 5 | 57 | 19 | 57 | 1 | 0 | 0 | 1 | 0 | 0 | 0 | 0 |
| meta-llama/llama-4-scout | 3 | 58 | 22 | 57 | 0 | 0 | 0 | 0 | 0 | 0 | 0 | 0 |
| nvidia/llama-3.3-nemotron-super-49b-v1.5 | 4 | 57 | 20 | 57 | 0 | 0 | 0 | 1 | 0 | 0 | 0 | 0 |
| nvidia/nemotron-3-super-120b-a12b | 5 | 47 | 18 | 50 | 7 | 0 | 0 | 1 | 0 | 3 | 1 | 6 |

*Note.* The table presents counts of automated classification of LLM and USPSTF text as (i) recommending a preventive activity, (ii) not recommending a preventive activity, (iii) equivocating / encouraging the user to seek out additional information or consultation, or (iv) stating a lack of information or evidence to make a recommendation. To conserve space, combinations of classifications that LLMs never used (i.e. columns of zero cells) are not shown.

# Supplementary Materials, Section 14

| No-Disclaimer Prompt | LLM Equivocates / USPSTF Equivocates | LLM Equivocates / USPSTF Recommends | LLM Equivocates / USPSTF Recommends Against | LLM Equivocates / USPSTF States Lack of Evidence | LLM Recommends / USPSTF Equivocates | LLM Recommends / USPSTF Recommends | LLM Recommends / USPSTF Recommends Against | LLM Recommends / USPSTF States Lack of Evidence | LLM Recommends Against / USPSTF Recommends Against | LLM Recommends Against / USPSTF States Lack of Evidence | LLM States Lack of Evidence / USPSTF States Lack of Evidence |
| --- | --- | --- | --- | --- | --- | --- | --- | --- | --- | --- | --- |
| 2.5-flash | 4 | 8 | 10 | 31 | 1 | 43 | 1 | 19 | 9 | 3 | 0 |
| 3-flash-preview | 3 | 9 | 3 | 48 | 0 | 45 | 1 | 6 | 13 | 0 | 1 |
| claude-opus-4-8 | 5 | 15 | 6 | 48 | 0 | 37 | 0 | 3 | 12 | 1 | 0 |
| claude-sonnet-4-6 | 6 | 16 | 10 | 45 | 0 | 41 | 1 | 5 | 11 | 1 | 2 |
| gpt-4.1-2025-04-14 | 4 | 12 | 5 | 36 | 0 | 43 | 1 | 12 | 15 | 4 | 2 |
| gpt-5.5-2026-04-23 | 4 | 8 | 5 | 49 | 0 | 48 | 2 | 5 | 9 | 0 | 1 |
| meta-llama/llama-4-maverick | 5 | 15 | 11 | 46 | 1 | 37 | 1 | 8 | 9 | 0 | 0 |
| meta-llama/llama-4-scout | 5 | 21 | 17 | 43 | 0 | 26 | 1 | 7 | 4 | 3 | 0 |
| nvidia/llama-3.3-nemotron-super-49b-v1.5 | 5 | 9 | 10 | 44 | 0 | 43 | 1 | 10 | 9 | 1 | 0 |
| nvidia/nemotron-3-super-120b-a12b | 4 | 6 | 1 | 35 | 2 | 50 | 0 | 14 | 17 | 3 | 1 |

| Iterative Prompt | LLM Equivocates / USPSTF Equivocates | LLM Equivocates / USPSTF Recommends | LLM Equivocates / USPSTF Recommends Against | LLM Equivocates / USPSTF States Lack of Evidence | LLM Recommends / USPSTF Equivocates | LLM Recommends / USPSTF Recommends | LLM Recommends / USPSTF Recommends Against | LLM Recommends / USPSTF States Lack of Evidence | LLM Recommends Against / USPSTF Equivocates | LLM Recommends Against / USPSTF Recommends Against | LLM Recommends Against / USPSTF States Lack of Evidence | LLM States Lack of Evidence / USPSTF States Lack of Evidence |
| --- | --- | --- | --- | --- | --- | --- | --- | --- | --- | --- | --- | --- |
| 2.5-flash | 3 | 40 | 16 | 47 | 0 | 15 | 1 | 6 | 0 | 5 | 3 | 0 |
| 3-flash-preview | 2 | 10 | 4 | 47 | 0 | 45 | 1 | 7 | 0 | 17 | 1 | 0 |
| claude-opus-4-8 | 6 | 36 | 10 | 54 | 0 | 19 | 0 | 1 | 0 | 8 | 0 | 0 |
| claude-sonnet-4-6 | 3 | 5 | 8 | 34 | 2 | 51 | 0 | 17 | 1 | 12 | 3 | 0 |
| gpt-4.1-2025-04-14 | 3 | 5 | 4 | 34 | 1 | 46 | 0 | 11 | 0 | 16 | 5 | 0 |
| gpt-5.5-2026-04-23 | 3 | 3 | 2 | 40 | 0 | 57 | 0 | 11 | 0 | 14 | 1 | 1 |
| meta-llama/llama-4-maverick | 5 | 49 | 19 | 57 | 0 | 6 | 0 | 0 | 0 | 3 | 0 | 0 |
| meta-llama/llama-4-scout | 4 | 45 | 18 | 56 | 1 | 9 | 0 | 1 | 0 | 2 | 0 | 0 |
| nvidia/llama-3.3-nemotron-super-49b-v1.5 | 6 | 20 | 16 | 54 | 0 | 32 | 1 | 3 | 0 | 4 | 0 | 0 |
| nvidia/nemotron-3-super-120b-a12b | 5 | 28 | 12 | 53 | 1 | 22 | 0 | 2 | 0 | 6 | 0 | 1 |

| Chain-of-Thought | LLM Equivocates / USPSTF Equivocates | LLM Equivocates / USPSTF Recommends | LLM Equivocates / USPSTF Recommends AGAINST | LLM Equivocates / USPSTF States a Lack of Evidence | LLM Recommends / USPSTF Equivocates | LLM Recommends / USPSTF Recommends | LLM Recommends / USPSTF Recommends AGAINST | LLM Recommends / USPSTF States a Lack of Evidence | LLM Recommends AGAINST / USPSTF Recommends AGAINST | LLM Recommends AGAINST / USPSTF States a Lack of Evidence | LLM States a Lack of Evidence / USPSTF States a Lack of Evidence |
| --- | --- | --- | --- | --- | --- | --- | --- | --- | --- | --- | --- |
| 2.5-flash | 13 | 54 | 27 | 119 | 2 | 104 | 0 | 33 | 31 | 10 | 0 |
| 3-flash-preview | 14 | 51 | 22 | 147 | 0 | 111 | 1 | 16 | 37 | 2 | 2 |
| claude-opus-4-8 | 16 | 90 | 35 | 163 | 0 | 65 | 0 | 3 | 25 | 2 | 0 |
| claude-sonnet-4-6 | 18 | 43 | 37 | 142 | 0 | 115 | 0 | 17 | 27 | 2 | 1 |
| gpt-4.1-2025-04-14 | 15 | 82 | 24 | 158 | 0 | 72 | 1 | 2 | 37 | 5 | 0 |
| gpt-5.5-2026-04-23 | 11 | 60 | 20 | 159 | 1 | 98 | 2 | 4 | 33 | 0 | 1 |
| meta-llama/llama-4-maverick | 15 | 137 | 49 | 163 | 0 | 27 | 0 | 4 | 14 | 1 | 1 |
| meta-llama/llama-4-scout | 15 | 151 | 53 | 169 | 0 | 13 | 0 | 0 | 7 | 1 | 0 |
| nvidia/llama-3.3-nemotron-super-49b-v1.5 | 10 | 105 | 40 | 170 | 0 | 59 | 0 | 2 | 17 | 0 | 0 |
| nvidia/nemotron-3-super-120b-a12b | 15 | 84 | 38 | 161 | 0 | 70 | 1 | 2 | 28 | 1 | 1 |

| Few-Shot | LLM Equivocates / USPSTF Equivocates | LLM Equivocates / USPSTF Recommends | LLM Equivocates / USPSTF Recommends Against | LLM Equivocates / USPSTF States a Lack of Evidence | LLM Recommends / USPSTF Recommends | LLM Recommends / USPSTF Recommends Against | LLM Recommends Against / USPSTF Recommends Against | LLM States a Lack of Evidence / USPSTF States a Lack of Evidence |
| --- | --- | --- | --- | --- | --- | --- | --- | --- |
| 2.5-flash | 12 | 174 | 55 | 171 | 1 | 1 | 5 | 0 |
| 3-flash-preview | 7 | 174 | 62 | 171 | 4 | 0 | 1 | 0 |
| claude-opus-4-8 | 11 | 171 | 59 | 170 | 2 | 0 | 2 | 1 |
| claude-sonnet-4-6 | 9 | 161 | 53 | 171 | 10 | 0 | 4 | 0 |
| gpt-4.1-2025-04-14 | 8 | 177 | 63 | 170 | 1 | 0 | 1 | 1 |
| gpt-5.5-2026-04-23 | 6 | 175 | 43 | 170 | 3 | 0 | 15 | 1 |
| meta-llama/llama-4-maverick | 13 | 173 | 65 | 171 | 0 | 0 | 0 | 0 |
| meta-llama/llama-4-scout | 10 | 173 | 64 | 171 | 2 | 0 | 0 | 0 |
| nvidia/llama-3.3-nemotron-super-49b-v1.5 | 8 | 173 | 65 | 171 | 2 | 0 | 0 | 0 |
| nvidia/nemotron-3-super-120b-a12b | 11 | 163 | 47 | 172 | 5 | 2 | 14 | 0 |

| Role-Based | LLM Equivocates / USPSTF Equivocates | LLM Equivocates / USPSTF Recommends | LLM Equivocates / USPSTF Recommends Against | LLM Equivocates / USPSTF States a Lack of Evidence | LLM Recommends / USPSTF Equivocates | LLM Recommends / USPSTF Recommends | LLM Recommends / USPSTF Recommends Against | LLM Recommends / USPSTF States a Lack of Evidence | LLM Recommends Against / USPSTF Recommends Against | LLM Recommends Against / USPSTF States a Lack of Evidence | LLM States a Lack of Evidence / USPSTF States a Lack of Evidence |
| --- | --- | --- | --- | --- | --- | --- | --- | --- | --- | --- | --- |
| 2.5-flash | 16 | 62 | 29 | 134 | 1 | 101 | 1 | 29 | 33 | 2 | 0 |
| 3-flash-preview | 13 | 42 | 25 | 149 | 1 | 124 | 2 | 13 | 37 | 3 | 0 |
| claude-opus-4-8 | 13 | 78 | 29 | 169 | 0 | 83 | 0 | 1 | 31 | 0 | 0 |
| claude-sonnet-4-6 | 15 | 57 | 40 | 149 | 0 | 102 | 0 | 17 | 18 | 0 | 0 |
| gpt-4.1-2025-04-14 | 13 | 73 | 27 | 154 | 0 | 93 | 1 | 8 | 32 | 6 | 0 |
| gpt-5.5-2026-04-23 | 16 | 33 | 20 | 143 | 1 | 133 | 4 | 18 | 37 | 1 | 0 |
| meta-llama/llama-4-maverick | 14 | 128 | 47 | 165 | 0 | 33 | 0 | 2 | 17 | 0 | 1 |
| meta-llama/llama-4-scout | 16 | 120 | 51 | 158 | 0 | 43 | 0 | 8 | 14 | 1 | 0 |
| nvidia/llama-3.3-nemotron-super-49b-v1.5 | 16 | 88 | 37 | 162 | 1 | 73 | 1 | 8 | 22 | 0 | 0 |
| nvidia/nemotron-3-super-120b-a12b | 14 | 76 | 35 | 166 | 0 | 85 | 0 | 3 | 22 | 0 | 0 |

# Supplementary Materials, Section 15

**Correspondence of LLM-Outputted Grades with USPSTF-Issued Grades**

| Model Version | Grades Do Not Match | Grades  Match | Number Tests | Proportion Matching |
| --- | --- | --- | --- | --- |
| gpt-4.1-2025-04-14 | 20 | 122 | 142 | 0.8592 |
| gpt-4o-2024-05-13 | 26 | 116 | 142 | 0.8169 |
| gpt-4-turbo-2024-04-09 | 29 | 113 | 142 | 0.7958 |
| gemini-2.5-flash-preview-04-17 | 31 | 111 | 142 | 0.7817 |
| claude-3-opus-20240229 | 31 | 111 | 142 | 0.7817 |
| gpt-4-0613 | 32 | 110 | 142 | 0.7746 |
| gpt-4o-2024-08-06 | 34 | 108 | 142 | 0.7606 |
| claude-3-5-sonnet-20241022 | 35 | 107 | 142 | 0.7535 |
| gpt-4-0125-preview | 39 | 103 | 142 | 0.7254 |
| gemini-2.0-flash | 40 | 102 | 142 | 0.7183 |
| gemini-1.5-pro | 42 | 100 | 142 | 0.7042 |
| gpt-4-1106-preview | 44 | 98 | 142 | 0.6901 |
| claude-3-7-sonnet-20250219 | 46 | 96 | 142 | 0.6761 |
| claude-3-5-sonnet-20240620 | 51 | 91 | 142 | 0.6408 |
| gpt-4.1-mini-2025-04-14 | 51 | 91 | 142 | 0.6408 |
| Llama-4-Maverick-17B-128E-Instruct-FP8 | 51 | 91 | 142 | 0.6408 |
| gpt-4-0314 | 59 | 83 | 142 | 0.5845 |
| gpt-4.1-nano-2025-04-14 | 59 | 83 | 142 | 0.5845 |
| Llama-4-Scout-17B-16E-Instruct-FP8 | 64 | 78 | 142 | 0.5493 |
| Llama-3.3-70B-Instruct | 65 | 77 | 142 | 0.5423 |
| gpt-4o-mini-2024-07-18 | 66 | 76 | 142 | 0.5352 |
| claude-3-sonnet-20240229 | 68 | 74 | 142 | 0.5211 |
| claude-3-5-haiku-20241022 | 69 | 73 | 142 | 0.5141 |
| gemini-1.5-flash | 71 | 71 | 142 | 0.5000 |
| claude-3-haiku-20240307 | 80 | 62 | 142 | 0.4366 |
| nvidia/llama-3.1-nemotron-70b-instruct | 81 | 61 | 142 | 0.4296 |
| nvidia/llama-3.3-nemotron-super-49b-v1 | 94 | 48 | 142 | 0.338 |
| Llama-3.3-8B-Instruct | 97 | 45 | 142 | 0.3169 |
| Total | 1475 | 2501 | 3976 | 0.6290 |

*Note.* The second column from the left of the table presents the count of instances in which an LLM output a grade for a particular preventive care activity, for a given population, which did not match the grade that the USPSTF had issued for the same preventive care activity in said population. The third column from the left presents the tally of matches between LLM output and USPSTF grades in such instances. The second to rightmost column presents the total number of instances in which an LLM was prompted to grade a preventive care activity for a given population. The rightmost column presents the proportion of accurate tests.

**Table 3. Correspondence of LLM-Outputted Grades with USPSTF-Issued Grades**

| Model Version | Grades Do Not Match | Grades  Match | Number Tests | Proportion Matching |
| --- | --- | --- | --- | --- |
| 2.5-flash | 39 | 103 | 142 | 0.7254 |
| 3-flash-preview | 8 | 134 | 142 | 0.9437 |
| claude-opus-4-8 | 10 | 132 | 142 | 0.9296 |
| claude-sonnet-4-6 | 27 | 115 | 142 | 0.8099 |
| gpt-4.1-2025-04-14 | 18 | 124 | 142 | 0.8732 |
| gpt-5.5-2026-04-23 | 8 | 134 | 142 | 0.9437 |
| meta-llama/llama-4-maverick | 54 | 88 | 142 | 0.6197 |
| meta-llama/llama-4-scout | 67 | 75 | 142 | 0.5282 |
| nvidia/llama-3.3-nemotron-super-49b-v1.5 | 28 | 114 | 142 | 0.8028 |
| nvidia/nemotron-3-super-120b-a12b | 29 | 113 | 142 | 0.7958 |

*Note.* The second column from the left of the table presents the count of instances in which an LLM output a grade for a particular preventive care activity, for a given population, which did not match the grade that the USPSTF had issued for the same preventive care activity in said population. The third column from the left presents the tally of matches between LLM output and USPSTF grades in such instances. The second to rightmost column presents the total number of instances in which an LLM was prompted to grade a preventive care activity for a given population. The rightmost column presents the proportion of accurate tests.

# Supplementary Materials, Section 16

Joint Distribution of LLM-Generated and USPSTF Grades by Model, Wave 1

***Note.*** The figure visualizes the cross tabulation of LLM-generated grades and grades issued by the USPSTF for each preventive care activity using the same conventions as Figure 3, but splits the study data by model. Darker shading indicates higher cell counts.

Joint Distribution of LLM-Generated and USPSTF Grades by Model, Wave 2

***Note.*** The figure visualizes the cross tabulation of LLM-generated grades and grades issued by the USPSTF for each preventive care activity using the same conventions as Figure 4, but splits the study data by model. Darker shading indicates higher cell counts.

# Supplementary Materials References

[1]	A. Holtzman, J. Buys, L. Du, M. Forbes, and Y. Choi, “The curious case of neural text degeneration, v2,” *arXiv*, 2020.

[2]	Y. F. Qingyue Wang, Yanan Cao, Shuai Wang, Zhiliang Tian, Liang Ding, “Recursively Summarizing Enables Long-Term Dialogue Memory in Large Language Models,” *arXiv,* vol. 2308.15022 2023.
